# Target trial emulation shows that supported causal effects of religious attendance on well-being are selective

**DOI:** 10.1017/ehs.2026.10043

**Published:** 2026-03-25

**Authors:** Joseph A. Bulbulia, Don E. Davis, Crystal Park, Kenneth G. Rice, Geoffrey Troughton, Daryl R. Van Tongeren, Chris G. Sibley

**Affiliations:** 1 Psychology, Victoria University of Wellington, Wellington, New Zealand; 2 Matheny Center for the Study of Stress, Trauma, and Resilience, Georgia State University, Atlanta, GA, USA; 3 Department of Psychological Sciences, University of Connecticut, Storrs, CT, USA; 4 School of Social and Cultural Studies, Victoria University of Wellington, Wellington, New Zealand; 5 Psychology, Hope College Department of Psychology, Holland, MI, USA; 6 School of Psychology, University of Auckland - City Campus, Auckland, New Zealand

**Keywords:** longitudinal panel, New Zealand, outcome-wide, pair bonding, positivity assumption, ritual

## Abstract

Religious service attendance is associated with better well-being, but observational associations do not establish causation. We analyse six annual waves of the New Zealand Attitudes and Values Study (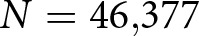) to estimate causal effects of monthly attendance on 24 well-being indicators using target trial emulation. Deterministic ‘make everyone attend’ contrasts fail positivity: only 2–3% of non-attenders initiate attendance per year. We therefore estimate supported stochastic interventions (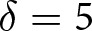) among baseline non-attenders (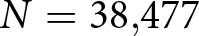) using a sequentially doubly robust estimator with cross-validated machine learning. Effects are selective: small gains appear in meaning and purpose, forgiveness, and sexual satisfaction, with little movement in somatic health, psychological distress, social belonging, or perceived social support. A comparison exposure (+1 hour per week socialising with others) does not reproduce the pattern. We interpret the selective pattern through a prominent cooperative account of religion: gains concentrate in coordination-relevant domains rather than in direct health pathways.

## Introduction

Religious beliefs and rituals are widespread across human societies, and throughout human prehistory, despite energetic, material, and opportunity costs (Sterelny, 2018, 2023). Why would selection retain such expensive behaviours? A prominent view in cultural evolution is that religion evolved to enhance cooperation (Sterelny, 2018): religious costs are signals that render cooperative exchange more reliable and predictable (Alcorta & Sosis, 2005; Bulbulia, 2004; Sosis & Bressler, 2003). More broadly, religious systems motivate norm enforcement, signal cooperative commitments, and make the outcomes of coordination problems more predictable (Bulbulia, 2012; Bulbulia et al., 2013; Johnson, 2009; Norenzayan, 2013; Norenzayan et al., 2016). Evidence supports the persistence of these cooperative functions. Religion is more prevalent where secular institutions are weaker (Valencia Caicedo et al., 2023); collective shocks increase religiosity, sometimes without matching gains in perceived health (Bentzen, 2019; Chen, 2010; Chvaja et al., 2024; Sibley & Bulbulia, 2012); experimentally induced insecurity raises willingness to join groups that enforce shared rules (Lang et al., 2024); moralising beliefs are more prevalent in harsher ecologies (Botero et al., 2014); and rising security predicts religious decline (Barro & McCleary, 2003; Norris & Inglehart, 2004).

A large literature links religious involvement with better mental and physical health. Meta-analyses report associations between religious service attendance and lower mortality, reduced depression, greater life satisfaction, and improved subjective health, with attendance emerging as a stronger predictor than private devotion or self-rated religiosity (Iyer & Rosso, 2022; Koenig et al., 2012; Li et al., 2016; VanderWeele, 2021).

Notably, however, the view that religion evolved to enhance cooperation, which makes sense of religion’s costs, does not obviously predict direct health effects: if religion were nature’s medicine, salubrious effects should persist without the costs that religious commitment entails. Indirect benefits such as longevity (Li et al., 2016) and higher fertility (Chvaja et al., 2026; Shaver et al., 2020) might arise from enhanced cooperation, or as signals that enhance cooperation (Alcorta & Sosis, 2005; Bulbulia, 2006), or both. If health benefits are by-products of cooperation-enhancing functions, that enhance social integration and coordinated commitment, then well-being effects should concentrate in coordination-relevant domains (relational repair, partnership stability, and as we argue, meaning and purpose) rather than spreading indiscriminately across all health indicators. Somatic health (sleep, body mass index, alcohol use) and psychological distress would not be expected to respond to a cooperation mechanism. A broad pattern of gains would count against this cooperative interpretation; a selective pattern concentrated in coordination-relevant domains would be consistent with it, though not confirmatory (we return to this distinction in the Discussion).

Whether the pattern is selective cannot be determined from associations alone. Most of the health evidence is cross-sectional, and longitudinal studies are scarce and typically adjust for a few confounders (Pawlikowski et al., 2019). The shortcomings run deeper still. The evidence in both the evolutionary and the religion-and-health literatures faces three further causal-identification constraints. First, even longitudinal studies rely on observational associations vulnerable to residual confounding by baseline health, prior religiosity, and other common causes (Beheim et al., 2021; Brown et al., 2023; Highland et al., 2019; Jokela & Laakasuo, 2024; Purzycki et al., 2018; Slingerland et al., 2020; Whitehouse et al., 2023). Second, even when investigators employ causal methods, the specific contrast being evaluated (the causal estimand: which intervention, on which population, compared with what alternative, measured when?) is often left implicit, obscuring practical interpretation. Where investigators make the contrast explicit, effect estimates shrink but do not vanish (Bulbulia et al., 2024; Van Tongeren et al., 2025). Third, whether the data support the estimated contrast (the positivity assumption: that every covariate-defined subgroup includes both attenders and non-attenders) is almost never checked, even though failures force extrapolation beyond the observed data. We use a target trial framework that specifies each causal contrast before estimation and checks whether the data can support it.

Here, we analyse six annual waves of the New Zealand Attitudes and Values Study (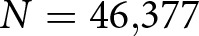) to estimate causal effects of religious attendance on 24 well-being indicators (Hernan & Robins, 2020; Hernán et al., 2016). We find that deterministic gain-of-attendance contrasts (everyone attends, compared with not attending) fail empirical support: among baseline non-attenders, only about 2–3% initiate monthly attendance per year (Supplement S4). We therefore focus on supported stochastic interventions among baseline non-attenders (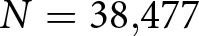), including a five-fold reduction in each person’s conditional probability of non-attendance (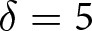), compared with the natural course (Williams & Díaz, 2023). Under the supported contrasts, effects are selective: gains concentrate in coordination-relevant domains, with minimal movement in somatic health or psychological distress.

## Methods

**Population.** (1) Baseline non-attenders (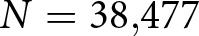) for the gain-of-attendance question. (2) Full baseline cohort (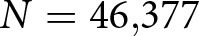) for the loss-of-attendance question. Both are drawn from the New Zealand Attitudes and Values Study and post-stratified (reweighted to match) the 2018 New Zealand Census.

**Design.** Six annual waves (2018–2023), with 2018 as the baseline. Attendance is intervened on in 2019 and 2022; outcomes are measured in 2023, one year after the final exposure.

**Exposure.** Monthly religious service attendance (at least once per month versus none), coded as a binary indicator at each intervention wave.

**Contrasts.** (1) *Gains:* What if each non-attender’s probability of non-attendance were divided by five (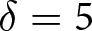), compared with the natural course? (2) *Losses:* What if all current attenders stopped, compared with the observed attendance rate?

**Outcomes.** 24 indicators across somatic health, psychological distress, meaning and purpose, gratitude and forgiveness, belonging and support, and sexual satisfaction (outcome-wide design).

**Confounding control.** 62 baseline covariates (demographics, personality, ideology, health behaviours, all 24 baseline outcomes) and 32 time-varying confounders (lagged outcomes at each exposure wave). Inverse-probability-of-censoring weights adjust for participant dropout.

**Estimation.** Sequentially doubly robust estimator with cross-validated machine-learning ensembles; produces valid estimates if either the outcome model or both the treatment and censoring models are correctly specified.

### Study design and sample

We used data from the New Zealand Attitudes and Values Study, an annual longitudinal national probability panel of New Zealand residents that assesses social attitudes, personality, ideology, health, and religion. The study started in 2009, is university-based and independently funded, and uses prize draws to incentivise participation. We draw from Time 10 (2018-2019) because this wave included a large probability sample refresh, recovering over 1.5% of the New Zealand adult resident population. Although the New Zealand Attitudes and Values Study is close to representative, it slightly under-samples men and people of Asian descent and over-samples women and Māori (the Indigenous people of New Zealand). To recover estimates from the sample to the target population, we post-stratify to the 2018 New Zealand Census margins for age, gender, and ethnicity (New Zealand European, Asian, Māori, Pacific) (Sibley, 2021). Additional study details are available at https://doi.org/10.17605/OSF.IO/75SNB (see Supplement S1).

**Figure 1.**
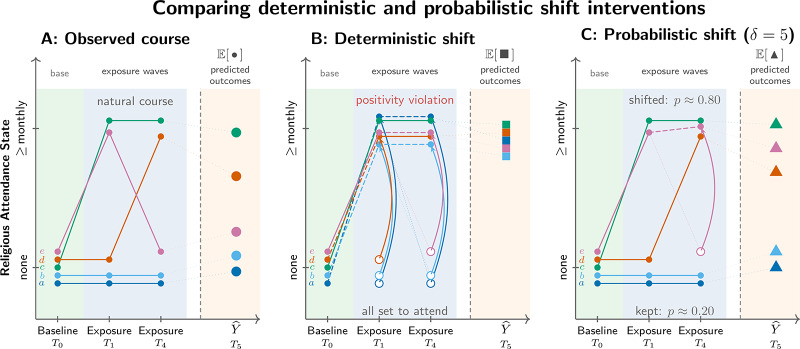
Comparing deterministic and probabilistic shift interventions applied to baseline non-attenders. Five illustrative persons (a--e), all non-attenders at baseline (T0, 2018), are tracked across two exposure waves (T1, 2019; T4, 2022) and a subsequent outcome measurement (T5, 2023). Attendance was not measured at T2 (2020) or T3 (2021), so the intervention operates at T1 and T4 only. Panel A shows the observed (natural) course. Panel B applies a deterministic intervention that sets all non-attenders to attend at each exposure wave; this regime violates positivity because virtually no one in the data follows this trajectory naturally. Panel C applies a probabilistic (incremental propensity score) intervention with delta = 5: at each exposure wave, every non-attender has probability ~0.80 of being shifted to attendance and probability ~0.20 of keeping their observed value. The causal estimand is the average difference in predicted outcomes between Panels C and A. Sample: baseline non-attenders (N = 38,477).

Each New Zealand Attitudes and Values Study wave spans 1 October of the stated year to 30 September of the next. Our analysis uses six waves (Time 10–Time 15, spanning 2018–2023). Because the attendance item was not fielded in 2020 and was incompletely fielded in 2021, the six-wave design effectively implements two intervention points on attendance (Year 1 and Year 4) and measures outcomes in the 2023 wave, one year later. Figure 1 summarises this structure; details on exposure missingness appear in Supplement S2.

### Exposure and outcomes

#### Exposure

The New Zealand Attitudes and Values Study measures religious service attendance with the item: *‘Do you identify with a religion and/or spiritual group? If yes, how many times did you attend a church or place of worship during the last month?’* Responses are open-ended counts per month; we rounded fractional responses to the nearest whole number. The incremental propensity score interventions required a binary indicator: any monthly attendance (1) versus no attendance (0). We coded those reporting no religious affiliation as zero and recorded missing data as NA. Participants with missing exposure data in the baseline wave were ineligible. We allow that the exposure may have affected attrition at the first wave by modelling censoring for this wave, as well as the others. The deterministic interventions described below use the same monthly-or-more threshold.

#### Outcomes

We use an outcome-wide design (VanderWeele et al., 2020), assessing 24 indicators across five domains: health (alcohol frequency, alcohol intensity, body mass index, exercise hours, sleep, short-form health); psychological well-being (anxiety, depression, fatigue, perfectionism, rumination); present-reflective states (body satisfaction, forgiveness, self-control, self-esteem, sexual satisfaction); life-reflective evaluations (gratitude, life satisfaction, meaning and purpose, meaning and sense, personal well-being index); and social connection (neighbourhood community, social belonging, social support). All outcomes are standardised to z-scores (mean 0, standard deviation 1); hence, results may be interpreted as effect sizes. Full definitions appear in Supplement S2.

### Target populations

We define two target populations, each anchored to the New Zealand adult resident population represented by the New Zealand Attitudes and Values Study baseline cohort in 2018 and calibrated to the 2018 New Zealand Census using post-stratification weights (Sibley, 2021).

The first is the **full baseline cohort** (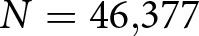), used for the deterministic loss-of-attendance analysis. Everyone who answered the questionnaire in 2018 and reported their attendance level was eligible. The second is the **baseline non-attender cohort** (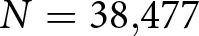), used for stochastic interventions on attendance probability. This restriction is necessary because these interventions target *initiation* of attendance, and we focus on the population for whom initiation is the relevant transition.

Subsequent non-response and loss to follow-up were treated as censoring events. We accounted for censoring using inverse-probability-of-censoring weights, so that all causal contrasts target the baseline cohort as if no one had dropped out (Laan & Robins, 2003).

### Causal framework

A causal effect compares two projected states of a population: how would outcomes differ if everyone experienced one condition versus another? In a randomised experiment, investigators create the contrast by assigning conditions. In observational data, investigators must construct it from assumptions. We use the target trial framework to discipline our analysis (Hernan & Robins, 2020; Hernán et al., 2016). A target trial is the randomised experiment we would ideally conduct: it specifies who is eligible, which interventions are compared, when outcomes are measured, and how participants are followed (Bulbulia, 2023). Randomly assigning people to attend religious services is neither feasible nor ethical, so we cannot run this experiment. We use the target trial specification to structure an observational analysis that answers the same question the trial would answer, subject to assumptions we state below. Because these assumptions cannot be guaranteed, we conduct sensitivity analyses.

#### Two kinds of intervention

As mentioned, a causal effect is a compariso of outcomes under one exposure level against outcomes under another, for a pre-defined target population. Both sides of this comparison must be specified. Much of the social-science literature on religion estimates single regression coefficients without specifying which two states are being compared. This practice invites confusion. A deterministic intervention asks: what if everyone in the population attended, compared with not attending (or vice versa)? A probabilistic intervention asks: what if attendance became more probable, compared with the observed rate (Kennedy, 2019)? These are different causal questions, Not only do they lead to different answers, they have different identification conditions (Hoffman et al., 2022). We initially specified contrasts of deterministic interventions, but in secular New Zealand fewer than 3% of non-attenders begin attending in any year. We learned the data cannot support the deterministic shift-up contrast: there is too little observed change (Supplement S4; Supplement S5). We therefore turned to stochastic interventions (technically, incremental propensity score interventions), which divide each person’s conditional probability of *not* initiating attendance by a factor of 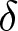 (Díaz et al., 2021). Intuitively, this is analogous to a setting in which attendance becomes substantially more accessible. Moderate (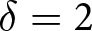) and stronger (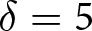) shifts are supported; a ten-fold shift (
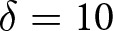
) is not. The reverse deterministic question (what if all attenders stopped, compared with the observed attendance rate?) is supported because transitions out of attendance occur more frequently.

#### Primary causal question

What would happen if, at each intervention wave, each baseline non-attender’s conditional probability of non-attendance were divided by five (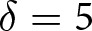), compared with the natural course? We also report a more conservative two-fold shift (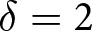) in the supplement. Outcomes are measured one year after the final exposure wave to reduce the risk that outcomes influenced the exposure rather than the other way around.

### Notation

We denote a policy as a sequence 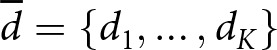 applied at 
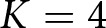
 post-baseline waves, of which waves 2 and 3 lack exposure measurement (attendance was not asked), so the intervention operates at waves 1 and 4 only. For any person, 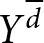 is the potential outcome under policy 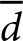. The corresponding population mean under that policy is 
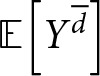
 (with a hypothetical no-censoring intervention). Our causal estimands are contrasts of these policy-specific means:

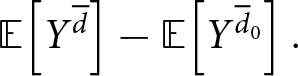


For the probabilistic attendance initiation analyses, the primary contrast is 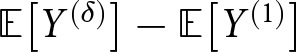, where 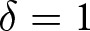 is the identity (natural-course) policy. A stochastic intervention with intensity 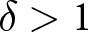 divides each person’s conditional probability of *not* initiating attendance by 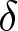. If 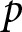 is the observed conditional probability of initiating attendance, the counterfactual initiation probability is:

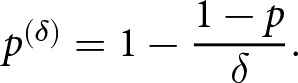


In words, 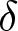 compresses the probability of remaining a non-attender. When baseline initiation is rare (
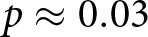
), this compression is large: 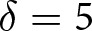 reduces the probability of not initiating from 0.97 to 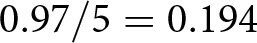, raising per-wave initiation probability from 0.03 to approximately 0.81. The intervention remains probabilistic, because no individual is deterministically forced to attend. However, the probability shift is substantial. Readers should bear this in mind when interpreting effect sizes: 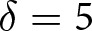 describes a world in which most non-attenders would begin attending at each intervention wave, not a marginal nudge.

#### Three assumptions

Causal identification rests on three assumptions: consistency, exchangeability, and positivity (Bulbulia, 2024b; Bulbulia et al., 2024). Consistency means that what we observe is what would have happened under the exposure actually received. Consistency also implies that treatments are comparable across individuals. Averaging over many forms of religious attendance (different traditions, congregational sizes, ritual styles) strains this assumption: even if estimates are unbiased, heterogeneous treatments make interpretation difficult. Exchangeability means that, conditional on measured covariates and prior history, exposure assignment is as if random, so no unmeasured confounding remains. Positivity means that each exposure level has non-zero probability within every covariate stratum used for adjustment. In plain terms, among people with similar measured histories, we must observe some who attend and some who do not. If not, the estimate must extrapolate beyond the data, which can produce unstable or misleading effects. Only positivity can be directly checked. Against the observed data, Below, we clarify how the positivity assumption fails for deterministic interventions that shift everyone to attendance. Detailed discussions of the three fundamental assumptions of causal inference appear in Supplement S3.

### Confounding control

We adjust for 62 baseline covariates (after dichotomisation of ordinal indicators) and 32 time-varying confounders at each exposure wave. Baseline adjustment includes demographics, personality, ideology, health behaviours, baseline exposure, and all 24 baseline outcomes, following outcome-wide guidance (VanderWeele et al., 2020). Baseline exposure and outcome variables are typically the strongest confounders in observational studies; any remaining bias would therefore have to arise from factors unrelated to these measured variables (VanderWeele et al., 2020). Time-varying adjustment includes lagged outcomes to preserve temporal ordering. This is a distinctive feature of the sequential design: because confounders such as health, distress, and social connection can change between exposure waves, and can themselves be affected by prior attendance, adjusting only at baseline would leave open confounding paths that update over time. Including lagged outcomes at each exposure wave blocks these paths. The full covariate inventory, wording, and coding appear in Supplement S2 and Supplement S3; the data structure for the six-wave study appears in Supplement S3. Table 1 summarises the baseline covariates.

**Table 1.** Baseline covariates used for confounding control. Religious identification (the baseline exposure) is included. The variables listed from General health onward, together with exercise hours, describe the outcome variables (24 total). Baseline masures of all such outcomes are also included for confounding control. In the six-wave analysis, lagged values of all outcomes at each exposure wave are included as time-varying confounders. Full covariate details in Supplement S2

Domain	Measure	Brief description	Coding
Demographics	Age	Years of age.	Continuous
	Gender	Self-described; indicator for male.	Binary (0/1)
	Born in New Zealand	Place of birth.	Binary (0/1)
	Ethnicity	Major ethnic groups (New Zealand European, Māori, Pacific,Asian).	Categorical
	Sexual orientation	Recoded to ‘not heterosexual’.	Binary (0/1)
	Parent status	Has children.	Binary (0/1)
	Partnered	In a romantic partnership.	Binary (0/1)
Sampling/design	Sample frame opt-in	Not sampled from electoral roll(opt-in).	Binary (0/1)
Socio-economic	Education level	Highest qualification (New ZealandQualifications Authority categories).	Ordinal
	Employment	Currently employed (incl. casual/self-employed).	Binary(0/1)
	Household income	Annual before-tax household income.	Log(income)
	Occupational prestige (New Zealand Socioeconomic Index)	Socioeconomic index of occupation, education, earnings.	Continuous(10-90)
	Area deprivation (New Zealand Deprivation Index 2018)	Census-linkeddeprivation decile.	Ordinal (1-10)
Geography	Urban-rural (Geographic Classification for Health 2018)	Accessibility/remoteness (5 levels).	Ordinal (1-5)
Personality (Mini International Personality Item Pool-6)	Agreeableness	Compassion/consideration (4 items).	1-7 Likert
	Conscientiousness	Dutifulness/orderliness (4 items).	1-7 Likert
	Extraversion	Sociability/talkativeness (4 items).	1-7 Likert
	Honesty-Humility	Modesty/lack of entitlement (4 items).	1-7Likert
	Neuroticism	Emotional instability (4 items).	1-7 Likert
	Openness	Imagination, interest in ideas (4 items).	1-7 Likert
Ideology	Political conservatism	Self-placement liberal-conservative.	1-7 scale
	Right-Wing Authoritarianism	Authority, conventionalism (shortRight-Wing Authoritarianism).	1-7 Likert
	Social Dominance Orientation	Preference for hierarchy (short SocialDominance Orientation).	1-7 Likert
Health behaviours	Smoking	Current tobacco use.	Binary (0/1)
Time use (weekly)	Hours looking after children	Weekly childcarehours.	Log(hours+1)
	Hours community activities	Weekly community/volunteering.	Log(hours+1)
	Hours commuting	Weekly commuting time.	Log(hours+1)
	Hours of housework	Weekly domestic work.	Log(hours+1)
	Hours of exercise	Weekly exercise; also analysed as outcome.	Log(hours+1)
Religion	Religious identification	Identifies with religion/spiritualgroup.	Binary (0/1)
General health	Short-Form Health	Self-rated health index.	1-7 scale
	Disability (6+ months)	Limiting condition 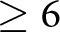 months.	Binary (0/1)
Somatic health	Fatigue	Frequency/level of tiredness.	Scale
	Body mass index	Calculated from self-reported height and weight.	Continuous (kg/m^2)
	Sleep hours	Average nightly sleep.	Continuous (hrs)
Alcohol use	Alcohol frequency	Weekly frequency (New Zealand HealthSurvey item).	Ordinal 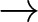 numeric
	Alcohol intensity	Typical drinks per occasion.	Continuous
Psych. distress	Anxiety (Kessler-6)	Latent factor from Kessler-6distress items.	Std. index
	Depression (Kessler-6)	Latent factor from Kessler-6 distress items.	Std. index
Psychological well-being	Rumination	Repetitive negative thinking.	Scale score
	Perfectionism	Standards/concerns (short scale).	Scale score
	Self-control	Capacity to regulate impulses.	Scale score
	Self-esteem	Global self-worth.	Scale score
Present-reflective	Body satisfaction	Satisfaction with appearance.	Scale score
	Sexual satisfaction	Satisfaction with sexual life.	Scale score
	Forgiveness	Dispositional/enacted forgiveness.	Scale score
Life-reflective	Gratitude	Dispositional gratitude.	Scale score
	Life satisfaction	Global life satisfaction.	Scale score
	Meaning and purpose	Life direction/goal structure.	Scale score
	Meaning and sense	Life coherence/meaningfulness.	Scale score
	Personal well-being index	Well-being across life domains.	Indexscore
Social	Social belonging	Acceptance/outsider (rev.)/shared attitudes.	1-7 Likert
	Perceived social support	Availability of supportive others.	Scalescore
	Neighbourhood community	Perceived cohesion/engagement.	Scalescore

### Estimation and sensitivity analysis

We estimate causal effects using the sequentially doubly robust estimator in the lmtp package (Díaz et al., 2021; Hoffman et al., 2024; Williams & Díaz, 2021), which combines non-parametric outcome models with non-parametric treatment and censoring models: estimates are valid if either the outcome model or both the treatment and censoring models are correctly specified. Machine-learning ensembles (SuperLearner) estimate the component models with five-fold cross-validation (Chernozhukov et al., 2018, Polley et al., 2023). Inverse-probability-of-censoring weights address attrition (Robins et al., 1999). We apply Bonferroni correction across the 24 outcomes (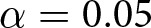) and report E-values to assess robustness to unmeasured confounding: E-values below 1.10 are treated as insufficiently robust even when confidence intervals exclude zero (VanderWeele & Ding, 2017). Missing baseline covariates were imputed using predictive mean matching (baseline missingness: 1.36 %); time-varying covariates were carried forward with missingness indicators; exposure missingness was handled via censoring weights. Technical details, formal notation, policy definitions, and learner diagnostics appear in Supplement S3 through Supplement S8; analysis code is available at the Open Science Framework (https://osf.io/75snb/, project: 2025-religious-attendance-causal-effects-on-multi-dimensional-well-being). We used the margot package for batch estimation, positivity assessment, and graphing (Bulbulia, 2024a). As a negative control, we estimate the effect of a deterministic increase in non-religious socialising (+1 hour per week, compared with the observed level) to test whether the attendance pattern is reducible to generic social contact.

## Results

### Cross-sectional associations

**Figure 2.**
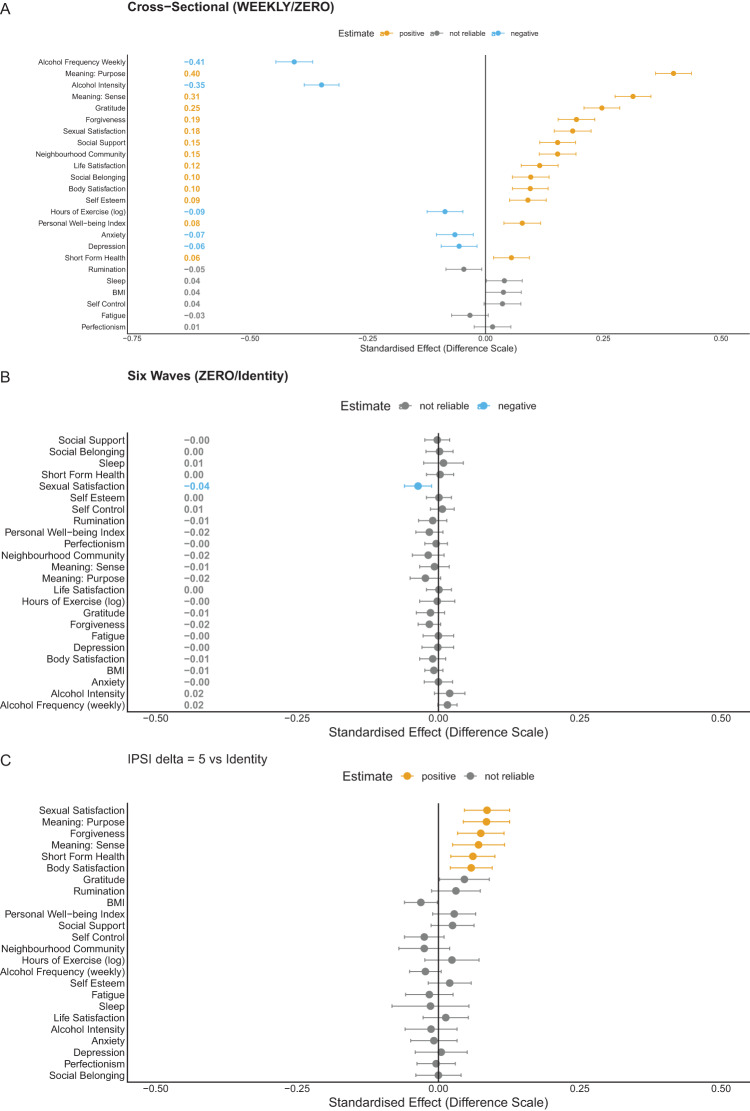
From associations to supported causal estimates. Each row is one outcome, grouped by domain; points show estimated effects in standardised units. Panel A shows descriptive cross-sectional associations. Panel B shows deterministic loss-of-attendance estimates (full cohort), where sexual satisfaction is the sole reliable effect. Panel C shows the primary stochastic intervention (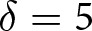) among baseline non-attenders; orange points survive Bonferroni correction and the E-value reliability threshold.

Cross-sectional associations between attendance and well-being are broadly favourable, replicating the meta-analytic pattern described in the Introduction. Panel A of Figure 2 shows positive associations across most outcomes, with the largest coefficients for meaning and purpose, gratitude, and life satisfaction. These coefficients are descriptive and should not be interpreted as causal effects. Full association estimates appear in Supplement S6 Part A.

### Positivity: what the data can and cannot answer

**Figure 3.**
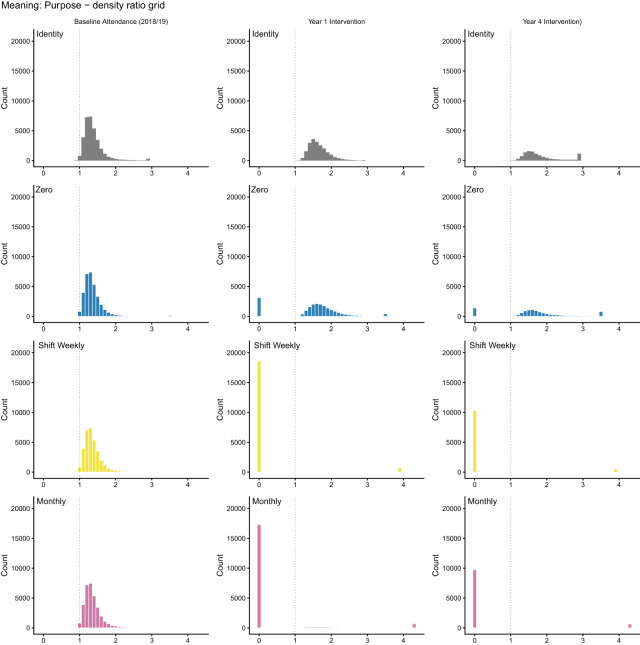
Positivity diagnostics for deterministic interventions in the full baseline cohort. Each panel shows the distribution of density ratios for a given policy. Ratios near 1.0 indicate good empirical support; ratios near 0 indicate the intervention requires extrapolation. Shift-up policies collapse towards 0; the identity and zero policies cluster near 1.

We initially specified the most natural deterministic question: what would happen if all non-attenders began attending? In secular New Zealand, upward transitions in attendance are rare (about 2–3% of baseline non-attenders initiate monthly attendance in any given year; see Supplement S4). The data cannot support a deterministic shift-up contrast without substantial extrapolation. Figure 3 and Table 2 confirm this: shift-up policies fail practical positivity, whereas the identity policy (no change) and the zero policy (stop attending) are supported. Deterministic shift-up results appear in Supplement S6 Part B for completeness; they should not be interpreted as causal estimates because they fail positivity.

**Table 2.** Summary of positivity diagnostics for deterministic interventions: shift-up interventions fail

Shift	Support	Zero %	Outside [0.1000, 10.0000]	Prod < 0.1000	Prod > 10.0000	Cum ESS
Identity	Adequate	75.779	0.724	0.000	0.724	8739.174
Shift Zero	Limited	75.779	11.645	9.966	1.679	6425.398
Shift Weekly	Limited	75.779	57.100	56.026	1.074	460.029
Shift Monthly	Limited	75.779	53.415	52.201	1.213	754.872

This positivity failure shows that sample size alone does not guarantee empirical support for a causal contrast. Despite 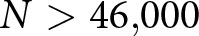, six waves, 62 baseline covariates, and machine-learning estimation, the deterministic initiation question lacks empirical support: the data contain too few upward transitions to sustain the attended-versus-not contrast on the initiation side. If a dataset of this quality cannot sustain the contrast, smaller studies making implicit causal claims about religion and health rely on still stronger extrapolation and should be interpreted with greater caution.

**Figure 4.**
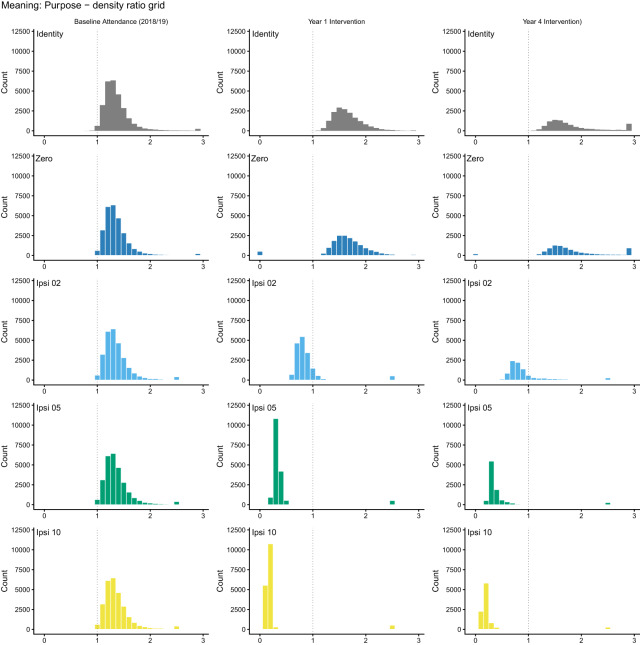
Positivity diagnostics for stochastic interventions among baseline non-attenders. As in the previous figure, density ratios near 1 indicate good support. The five-fold intervention satisfies positivity; the ten-fold intervention does not.

Figure 4 displays diagnostics for stochastic interventions among baseline non-attenders. Moderate (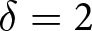) and stronger (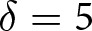) shifts satisfy positivity, but a ten-fold shift (
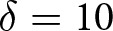
) fails (Table 3). This boundary demonstrates that labelling an intervention probabilistic does not guarantee the data can support it: even probability shifts can outrun observed variation.

**Table 3.** Summary of positivity diagnostics for stochastic interventions among baseline non-attenders

Shift	Support	Zero %	Outside [0.1000, 10.0000]	Prod < 0.1000	Prod > 10.0000	Cum ESS
Identity	Adequate	75.344	0.583	0.000	0.583	7539.602
Shift Zero	Adequate	75.344	2.968	2.221	0.746	7103.605
Ipsi 02	Adequate	75.344	0.050	0.000	0.050	5178.627
Ipsi 05	Caution	75.344	6.514	6.479	0.036	821.197
Ipsi 10	Limited	75.344	31.392	31.367	0.025	248.619

Making attendance more probable is not the same as making everyone attend. The causal effects we report for probabilistic iterventions define different causal questions from deterministic interventions.

### Deterministic loss of attendance

The deterministic loss-of-attendance question (what if all current attenders stopped, compared with the observed attendance rate?) satisfies positivity and provides a first causal result (Panel B of Figure 2). The only outcome meeting our reliability criterion is sexual satisfaction, which declines by 0.036 standard deviations (E-value bound 1.12; we define the reliability threshold below). Point estimates for meaning and purpose, neighbourhood community, forgiveness, and gratitude trend negatively but do not meet this threshold. Somatic indicators and psychological distress remain near zero. The difference from the cross-sectional associations is striking, though the comparison is not direct: the loss analysis answers a different question (what if attenders stopped, compared with continuing?) on a different population (the full cohort). The narrowing reflects both confounding adjustment and the change in causal question. Full deterministic loss-of-attendance results appear in Supplement S6 Part B.

### Causal effects of initiating attendance (primary analysis)

Given the positivity diagnostics above, we treat the five-fold stochastic intervention (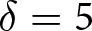) among baseline non-attenders as the primary supported contrast. Panel C of Figure 2 presents these results. Confidence intervals are Bonferroni-adjusted across the 24 outcomes (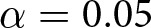), and we compute E-values from the adjusted intervals. We treat an E-value lower bound greater than 1.10 as our threshold for reliability (that is, unmeasured confounding would need to increase the risk by at least 10% to explain the result away).

The largest gains are in meaning and purpose (0.085 standard deviations; E-value bound 1.25), sexual satisfaction (0.086 standard deviations; E-value bound 1.25), and forgiveness (0.075 standard deviations; E-value bound 1.21), with additional reliable effects for meaning and sense (0.071; E-value bound 1.18), short-form health (0.061; E-value bound 1.16), and body satisfaction (0.058; E-value bound 1.16). Somatic indicators (body mass index, sleep, alcohol) and psychological distress indicators (anxiety, depression, fatigue, rumination) show point estimates near zero with confidence intervals spanning the null. Social belonging and perceived social support do not meet the E-value reliability threshold. Gains concentrate in a subset of domains, not across all aspects of well-being.

Sexual satisfaction converges across the loss and initiation analyses, which differ in target population, intervention direction, and sources of potential bias, strengthening confidence in this result.

The selective profile is not an artefact of the intervention’s intensity. A more conservative two-fold stochastic intervention (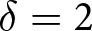) yields the same directional pattern with smaller magnitudes: sexual satisfaction (0.054; E-value bound 1.17), meaning and purpose (0.050; E-value bound 1.15), and forgiveness (0.046; E-value bound 1.13) remain reliable. The consistency across both supported contrasts strengthens the selectivity finding. Full results appear in Supplement S6 Part C.

### Negative control: socialising

A negative-control analysis examined whether an increase in non-religious socialising (+1 hour per week) could produce comparable gains. It did not: no reliable effects emerged. This comparison is not a clean negative control in the strict sense, because non-religious socialising can plausibly affect well-being through pathways unrelated to religious attendance. The comparison is nevertheless informative: because attending religious services may be considered a form of socialising, any shared social-contact mechanism would bias the comparison towards producing an effect, which makes the absence of an effect more informative. The socialising intervention itself strains positivity and should be interpreted cautiously. Results appear in Supplement S6 Part B and Supplement S8.

**Reliable causal effects.** Under the five-fold stochastic attendance intervention (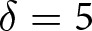), compared with the natural (observed) course, meaning and purpose, sexual satisfaction, and forgiveness show gains that survive Bonferroni correction and have 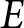-value lower bounds exceeding 1.10 (Figure 2, Panel C). Meaning and sense, short-form health, and body satisfaction show smaller effects that pass the same threshold. The two-fold intervention (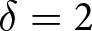) yields the same directional profile with smaller magnitudes.

**Selectivity.** Somatic health indicators (body mass index, sleep, alcohol) and psychological distress (anxiety, depression, fatigue, rumination) show no reliable movement. Social belonging and perceived social support show no reliable change, which argues against a generic social-contact mechanism.

**Convergence.** The deterministic loss-of-attendance analysis (full cohort, compared with continued attendance, passes positivity) provides converging evidence: sexual satisfaction is the sole outcome that reliably declines (Figure 2, Panel B). Agreement across designs with different target populations, intervention directions, and bias structures is stronger evidence than replication within one design.

**Negative control.** A deterministic +1 hour/week socialising intervention, compared with the observed level, does not replicate the attendance pattern, although this comparison is not a clean negative control and should be interpreted cautiously. This intervention approaches the boundary of what the data can support; interpret cautiously.

**Positivity as a finding.** The most natural causal question (‘make everyone attend, compared with not attending’) fails positivity despite 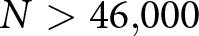, six waves, and 62 baseline covariates. Even a ten-fold probability shift (
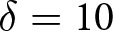
), which is formally probabilistic, fails the positivity criterion.

**Interpretation.** Effects concentrate in meaning, forgiveness, and sexual satisfaction. On coordination-based accounts emphasising shared ends, predictability, and relational repair.

## Discussion

These findings are exploratory. We interpret them through a prominent view in cultural evolution that religion evolved as a set of adaptations for cooperation (Sterelny, 2018). We do not test this account directly; we examine whether the observed pattern is consistent with it. Under the five-fold stochastic attendance intervention (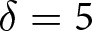), compared with the natural course, attendance produces small but reliable causal gains in meaning and purpose, forgiveness, and sexual satisfaction, with additional effects for meaning and sense, short-form health, and body satisfaction. Somatic indicators, psychological distress, social belonging, and perceived social support do not show clear effects under the supported contrasts.

### Meaning and purpose as a coordination-relevant domain

The meaning and purpose finding deserves close attention. Cooperation is often framed as a prisoner’s dilemma threatened by free-riding, but it has been argued that many real-world problems are better modelled as stag hunts: coordination dilemmas in which mutual gain is possible but depends on partner compliance, and lesser pay-offs are available from independent action (Alvard & Nolin, 2002; Binmore, 2008; Bulbulia, 2012; Skyrms, 2004). In a stag hunt, partners prefer to cooperate but each must trust others will reciprocate. The obstacle is uncertainty about partners’ choices, not temptation to cheat. Because the obstacle is uncertainty rather than temptation, two ingredients matter for success: shared ends and predictable partners.

Meaning and purpose may supply the first ingredient. Punishment-based theories of religion emphasise extrinsic enforcement: cooperation is sustained because defectors are punished (Johnson, 2009; Norenzayan, 2013). Intrinsic commitment works differently. A sense of purpose generates motivation that does not shift with the strategic environment: action is performed because it is right, not because the pay-off in a particular setting makes it worthwhile. Intrinsically motivated partners are more predictable because their behaviour abides in the face of shifting extrinsic interests, temporal discounting, and uncertainty about reciprocity (Gokhale et al., 2022). It has been noted that religious communities cultivate this commitment through shared narrative, public ritual, and moral frameworks that orient behaviour towards common ends, not merely through threats of supernatural punishment (Alcorta & Sosis, 2005; Norenzayan et al., 2016; Watts et al., 2015a). A shared sense of purpose functions as a guiding reference point that discourages second-guessing others’ intentions, rendering coordination more predictable. The gains we observe in meaning and purpose suggest a channel through which attendance could reduce strategic uncertainty in settings where optimally beneficial coordination is threatened by risk (stag-hunt dilemmas).

Forgiveness provides a complementary mechanism in support of cooperation. Sustained coordination inevitably involves conflict and defection. Repair after shocks is essential for long-term cooperation because unresolved conflict erodes trust and destabilises future coordination (Wilson, 2008). Gains in forgiveness (0.075 standard deviations under the five-fold intervention) suggest that attendance may strengthen the capacity for relational repair, restoring cooperation after conflict.

We do not find reliable increases in perceived social support or social belonging. Several interpretations are compatible with this absence. First, if cooperation under risk depends on intrinsic motivation rather than extrinsic social perceptions, then cooperative systems should not optimise perceived support or belonging; those perceptions leave cooperation subject to the vagaries of social judgement about whether extrinsic motivations are satisfied. Second, non-differential measurement error may attenuate effects selectively: if the social-support and belonging items are noisier than the meaning and forgiveness items, genuine effects would be harder to detect. Third, in secular, safe New Zealand, cooperative functions that evolved in small-scale societies may be somewhat atrophied in domains that depend on dense local networks of co-religionists. Our results do not distinguish these accounts, and all three are compatible with the observed pattern.

A negative-control analysis found that increasing non-religious socialising (+1 hour per week) does not produce comparable effects. The socialising intervention itself approaches the boundary of what the data can support. The absence persists despite this conservative test strengthens the inference that the attendance pattern is not reducible to generic social contact.

### Sexual satisfaction and pair-bond coordination

Sexual satisfaction is the most reliable finding in this study. It is the only outcome to survive the deterministic loss-of-attendance analysis (
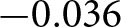
 standard deviations; E-value bound 1.12), and it shows the largest and most consistent gains across the probabilistic designs (0.054 to 0.086 standard deviations). Earlier cross-sectional studies report correlations between religiosity and marital sexual satisfaction (Dew et al., 2020), but cross-sectional designs cannot isolate causal effects because attenders differ from non-attenders in ways that independently affect relationship quality. Our results, which adjust for 62 baseline covariates including baseline sexual satisfaction, are consistent with a modest causal effect.

Sexual reproduction links genetic fates, making the pair bond a high-stakes cooperation problem (Darwin, 1872). Each partner’s reproductive success depends on the other’s behaviour, and both depend on the capacity to raise offspring together. The cost of relationship failure is large, and sustained cooperation requires aligning ends, maintaining predictability, managing conflict, sustaining partner trust, and coordinating child-rearing. Religious communities may stabilise pair-bond cooperation by reinforcing credible commitments, rendering intentions public, formalising norms of repair, and embedding partners within predictable local networks (Bulbulia et al., 2015). On cooperative accounts, small but reliable causal gains in sexual satisfaction suggest improved dyadic cooperation and partner trust. Body satisfaction co-occurs with sexual satisfaction under 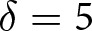. Much of this reasoning is speculative. Whether gains in sexual satisfaction and body satisfaction reflect security within committed partnerships, or instead cause such security, is ambiguous in our data. Both may function as mechanisms preserving the pair bond, an intriguing possibility for future research in the emerging literature on religion and cooperative breeding, to which Shaver et al. (Shaver et al., 2019) and Shaver et al. (Shaver et al., 2024) contribute.

A cooperation perspective also illuminates potentially darker dimensions of the religion–sexuality relationship. The same norms that stabilise pair-bond commitments can entrench sexual inequality, constrain bargaining power within relationships, and enforce conformity to gendered expectations (Deak et al., 2021). These costs may fall unevenly: effects could vary by gender, sexual orientation, religious tradition, and political economy (Price & Johnson, 2024; Shaver, 2015; Watts et al., 2015b, 2018). Our estimates are population averages in a contemporary secular setting where religious communities are small and diverse. They do not speak to the experience of specific groups. What they do suggest is that, on average, the cooperation-enhancing features of religious communities have a small but detectable effect on pair-bond satisfaction, consistent with the broader pattern of selective gains in coordination-relevant domains.

### Cooperative accounts as a framework for future work

As noted in the Introduction, a prominent view is that religion evolved to enhance cooperation (Sosis & Bressler, 2003; Sterelny, 2018). The selective pattern we observe is largely what cooperative accounts would lead us to expect, and interpreting results through this lens, we have argued, deepens explanation. It is important to understand, however, that contemporary causal effects and evolutionary origins are different questions that operate at different scales. Whether religion evolved for cooperation requires phylogenetic, archaeological, and cross-cultural evidence about selection pressures over deep time. Our study answers a proximate question: what does attendance do to well-being in a contemporary secular society? The two questions are connected but not interchangeable. Evolved functions may atrophy in modern settings, and modern effects may arise from institutional features (shared ritual, moral narrative, public commitment) that serve coordination regardless of their evolutionary origin. We cannot move from ‘attendance affects meaning and sexual satisfaction in New Zealand’ to ‘religion evolved because it affects meaning and sexual satisfaction’. The domain-specific pattern is coherent on cooperative accounts and incoherent on accounts that treat religion as a generic adaptation for health. Furthermore, evolutionary explanation can enrich understanding and stimulate further lines of research: does shared purpose render cooperation under risk less fragile? Does sexual satisfaction increase trust among couples? Does it increase body satisfaction? Is cooperation less sensitive to social belonging and support than to shared purpose? Does increasing risk amplify perceived social support and belonging? Could couple-level analyses reveal whether co-attendance moderates effects on sexual satisfaction and relationship conflict? These are lines for future work, not claims established here.

### Positivity failure as a finding about the field

The positivity failure we document has implications beyond this study. The most natural causal question about religion and health (‘what if non-attenders began attending, compared with not attending?’) lacks empirical support in a dataset with 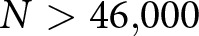, six annual waves, 62 baseline covariates, and machine-learning estimation. Studies in secular populations that make causal claims about religion and health are, in many cases, not even asking a question that can be answered: without specifying two states to be compared, there is no causal contrast to estimate. And where a contrast is specified, positivity may fail. The incremental intervention is not a methodological workaround; it is a better-supported question, comparing a world in which non-attendance becomes less probable with the observed natural course. The selective-not-broad result under supported interventions suggests that making attendance more accessible would not produce sweeping health benefits but would modestly strengthen the psychological capacities that support coordination: meaning, relational repair, and dyadic stability.

## Limitations

First, our analyses estimate total effects of specified changes in attendance, not mediated pathways. If attendance improves meaning, and meaning improves sexual satisfaction, both appear as effects of attendance. Decomposing the causal chain would require additional assumptions and a different estimator. The gains we observe could arise from institutional affordances, residual confounding by unmeasured selection, signalling, or material resources; we cannot distinguish these explanations.

Second, causal identification assumes consistency conditional on the 62 baseline covariates and 32 time-varying confounders we measure. If unmeasured common causes of attendance and well-being remain after this adjustment, estimates are biased; E-values quantify how strong such confounding would need to be.

Third, measurement error in self-reported attendance can pull estimates towards zero, meaning actual effects may be larger than we report (Bulbulia, 2024d). Conversely, error in measured confounders could inflate estimates.

Fourth, effects probably vary by religious tradition, belief strength, upbringing, and relationship status, yet statistical power constrains us to population averages. Our time horizon (one year after the final exposure wave) may miss effects that unfold over longer periods or dissipate quickly.

Fifth, generalisability is bounded: childhood religious socialisation lies outside what adult panel data can identify, however large. Causal inference from observational data is constrained by where the data provide support; like an astronomer who can only study the sky through gaps in the cloud cover, we can only answer questions about interventions that the data allow us to evaluate. Replication across traditions, life stages, and socio-political contexts is essential because our estimates are specific to a single secular setting.

## Conclusion

In secular New Zealand, substantially increasing the probability of initiating religious attendance among non-attenders (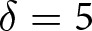), compared with the natural course, produces small but reliable causal gains in meaning and purpose, forgiveness, and sexual satisfaction. These gains are selective: somatic health, psychological distress, social belonging, and perceived social support show no reliable movement. Sexual satisfaction converges across two designs that differ in target population, intervention direction, and sources of potential bias. A negative control (non-religious socialising) does not replicate the pattern.

A recurring point in this study is that *there is no single quantity that can be called ‘the causal effect of religious attendance*’. Different causal contrasts yield different answers, and several natural questions turned out to be unanswerable because the data could not support them. For evolutionary human science, the selective pattern is coherent on cooperative accounts and incoherent on accounts that treat religion as a generic adaptation for health: the domains that respond are those tied to coordination (meaning, forgiveness, pair-bond satisfaction), not those tied to generic health.

The broader lesson is methodological. In causal inference, as in the tango, it takes two: two states to compare, a precisely stated target population, and evidence that the data can support each side of the comparison. Without clearly stated causal contrasts and satisfactory evidence, even an accurately estimated coefficient is uninterpretable.

## Supporting information

10.1017/ehs.2026.10043.sm001 Bulbulia et al. supplementary material Bulbulia et al. supplementary material

## Data Availability

The data described in the paper are part of the New Zealand Attitudes and Values Study. Members of the New Zealand Attitudes and Values Study management team and research group hold full copies of the study’s data. A de-identified dataset containing only the variables analysed in this manuscript is available upon request from the corresponding author or any member of the New Zealand Attitudes and Values Study advisory board for replication or checking of any published study using New Zealand Attitudes and Values Study data. Analysis code is available at https://osf.io/75snb/files/osfstorage.

## Supplementary material

The supplementary material for this article can be found at https://doi.org/10.1017/ehs.2026.10043.

## Author statement

All authors contributed to the manuscript. JAB did the analysis, developed the approach, and wrote the manuscript, with help from all authors. CGS led data collection. JAB, DED, CGS, KGR, and GT obtained funding.
