# Supplementary material for: Target trial emulation shows that supported causal effects of religious attendance on well-being are selective
Source: Evol Hum Sci. 2026 Mar 25;8:e16. doi: 10.1017/ehs.2026.10043 (PMC13122395; doi:10.1017/ehs.2026.10043)
Supplement: Bulbulia et al. supplementary material [file S2513843X26100437sup001.pdf]

Supplementary Materials

For: Target Trial Emulation Shows That Supported Causal Effects of Religious Attendance on Well-Being Are Selective (Bulbulia *et al.* 2026)

Joseph A. Bulbulia* 0000-0002-5861-2056[1], Don E. Davis 0000-0003-3169-6576[2], Crystal Park 0000-0001-6572-7321[3], Kenneth G. Rice 0000-0002-0558-2818[4], Geoffrey Troughton 0000-0001-7423-0640[5], Daryl R. Van Tongeren 0000-0002-1810-9448[6], Chris G. Sibley 0000-0002-4064-8800[7]

[1]Victoria University of Wellington, New Zealand
[2]Georgia State University, Matheny Center for the Study of Stress, Trauma, and Resilience
[3]University of Connecticut, Department of Psychological Sciences
[4]Georgia State University, Matheny Center for the Study of Stress, Trauma, and Resilience
[5]School of Social and Cultural Studies, Victoria University of Wellington
[6]Hope College
[7]School of Psychology, University of Auckland

2026-04-11

*Correspondence: joseph.bulbulia@vuw.ac.nz

Supplement S1: Daily Data Collection

Figure 1 presents the New Zealand Attitudes and Values Study Data Collection (2018 retained cohort) from 2018-2024 (New Zealand Attitudes and Values Study time 10–time 15).

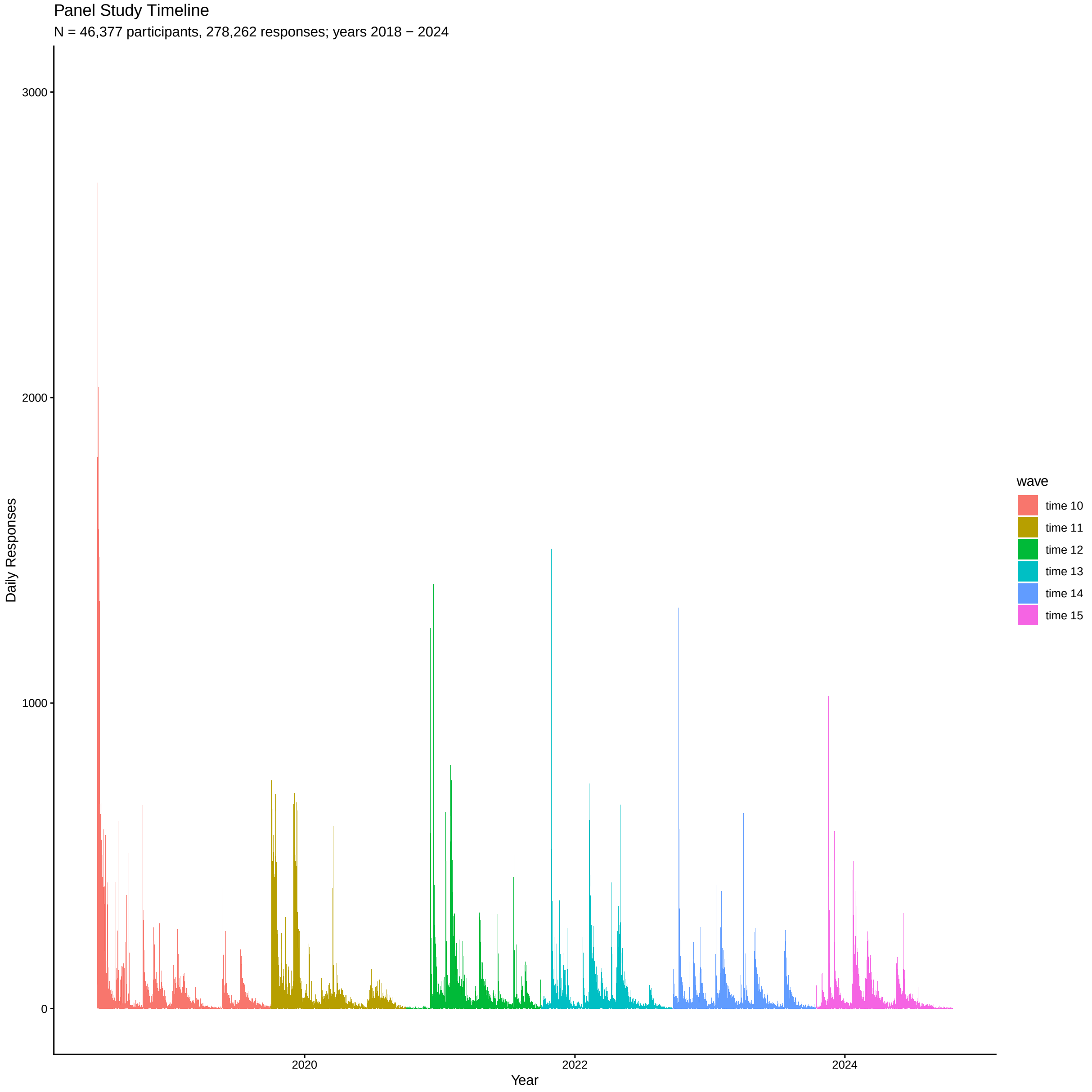

Figure 1: Histogram of New Zealand Attitudes and Values Study Daily Data Collection for Time 10 cohort: years 2018-2024.

Supplement S2: Measures and Demographic Statistics

Baseline Measures

Baseline Covariates

Age

We asked participants' ages in an open-ended question ("What is your age?" or "What is your date of birth") (Sibley 2021).

Items:

- What is your date of birth?

Agreeableness

Mini-IPIP6 Agreeableness dimension: (i) I sympathize with others' feelings. (ii) I am not interested in other people's problems. (r) (iii) I feel others' emotions. (iv) I am not really interested in others. (r) (Sibley *et al.* 2011).

Items:

- I sympathize with others' feelings.
- I am not interested in other people's problems.
- I feel others' emotions.
- I am not really interested in others (reversed).

Alcohol Frequency

Participants could chose between the following responses: '(1 = Never - I don't drink, 2 = Monthly or less, 3 = Up to 4 times a month, 4 = Up to 3 times a week, 5 = 4 or more times a week, 6 = Don't know)' (Health 2013).

Items:

- "How often do you have a drink containing alcohol?"

Alcohol Intensity

Participants responded using an open-ended box (Health 2013).

Items:

- "How many drinks containing alcohol do you have on a typical day when drinking alcohol? (number of drinks on a typical day when drinking)"

Social Belonging

We assessed felt belongingness with three items adapted from the Sense of Belonging Instrument (Hagerty & Patusky, 1995): (1) "Know that people in my life accept and value me"; (2) "Feel like an outsider"; (3) "Know that people around me share my attitudes and beliefs". Participants responded on a scale from 1 (Very Inaccurate) to 7 (Very Accurate). The second item was reversely coded (Hagerty and Patusky 1995).

Items:

- Know that people in my life accept and value me.
- Feel like an outsider (reversed).
- Know that people around me share my attitudes and beliefs.

Born in NZ

Coded binary (1 = New Zealand; 0 = elsewhere.) (Sibley 2021).

Items:

- Where were you born? (please be specific, e.g., which town/city?)

Conscientiousness

Mini-IPIP6 Conscientiousness dimension: (i) I get chores done right away. (ii) I like order. (iii) I make a mess of things. (r) (iv) I often forget to put things back in their proper place. (r) (Sibley *et al.* 2011).

Items:

- I get chores done right away.
- I like order.
- I make a mess of things.
- I often forget to put things back in their proper place.

Education Level

We asked participants, 'What is your highest level of qualification?'. We coded participants' highest finished degree according to the New Zealand Qualifications and Credentials Framework (NZQCF), which comprises 10 levels (Levels 1-10) of increasing complexity and depth of knowledge. We added a Level 0 category to represent no formal qualification, resulting in an 11-category ordinal measure (0-10). The levels are defined as follows: Level 0 represents no formal qualification. Levels 1-3 cover basic to operational knowledge. Level 4 covers broader operational and theoretical knowledge. Level 5 covers broad operational or technical knowledge within a specific field. Level 6 covers specialised technical or theoretical knowledge with depth. Level 7 includes bachelor's degrees, graduate certificates, and graduate diplomas. Level 8 includes bachelor honours degrees, postgraduate certificates, and postgraduate diplomas. Level 9 represents master's degrees. Level 10 represents doctoral degrees. To ensure adequate cell counts and satisfy the positivity assumption required for causal inference, we coarsened the 11-category measure (0-10) into seven ordinal categories: no qualification (Level 0), certificates Levels 1-4 (foundation through broader vocational qualifications), certificates Levels 5-6 (advanced certificates and diplomas), bachelor's degree (Level 7), postgraduate qualifications (Level 8), master's degree (Level 9), and doctorate (Level 10). This coarsening preserves meaningful educational gradients while ensuring sufficient sample sizes across all covariate strata. In our statistical models, these ordinal categories are represented as binary indicators (Sibley 2021).

Items:

- What is your highest level of qualification?

Employed (Binary)

Binary response: (0 = No, 1 = Yes) (Statistics New Zealand 2017).

Items:

- Are you currently employed (This includes self-employed of casual work)?

Ethnicity

Coded string: (1 = New Zealand European; 2 = Māori; 3 = Pacific; 4 = Asian) (Statistics New Zealand 2017).

Items:

- Which ethnic group(s) do you belong to?

Extraversion

Mini-IPIP6 Extraversion dimension: (i) I am the life of the party. (ii) I don't talk a lot. (r) (iii) I keep in the background. (r) (iv) I talk to a lot of different people at parties (Sibley *et al.* 2011).

Items:

- I am the life of the party.
- I don't talk a lot (reversed).
- I keep in the background (reversed).
- I talk to a lot of different people at parties.

Hlth Disability Binary

We assessed disability with a one-item indicator adapted from Verbrugge (1997). It asks, "Do you have a health condition or disability that limits you and that has lasted for 6+ months?" (1 = Yes, 0 = No) (Verbrugge 1997).

Items:

- Do you have a health condition or disability that limits you and that has lasted for 6+ months?

Honesty Humility

Mini-IPIP6 Honesty-Humility dimension: (i) I feel entitled to more of everything. (r) (ii) I deserve more things in life. (r) (iii) I would like to be seen driving around in a very expensive car. (r) (iv) I would get a lot of pleasure from owning expensive luxury goods. (r) (Sibley *et al.* 2011).

Items:

- I feel entitled to more of everything (reversed).
- I deserve more things in life (reversed).
- I would like to be seen driving around in a very expensive car (reversed).
- I would get a lot of pleasure from owning expensive luxury goods (reversed).

log Hours Children

We took the natural log of the response + 1 (Sibley *et al.* 2011).

Items:

- Hours spent…looking after children.

log Hours Community

No information available for this variable.

log Hours Commute

We took the natural log of the response + 1 (Sibley 2021).

Items:

- Hours spent…travelling/commuting.

log Hours of Exercise

We took the natural log of the response + 1 (Sibley *et al.* 2011).

Items:

- Hours spent…exercising/physical activity.

log Hours Housework

We took the natural log of the response + 1 (Sibley *et al.* 2011).

Items:

- Hours spent…housework/cooking.

log Household Income

We took the natural log of the response + 1 (Sibley 2021).

Items:

- Please estimate your total household income (before tax) for the year XXXX.

Male (Binary)

Here, we coded all those who responded as Male as 1, and those who did not as 0 (Fraser *et al.* 2020).

Items:

- We asked participants' gender in an open-ended question: "what is your gender?"

Neuroticism

Mini-IPIP6 Neuroticism dimension: (i) I have frequent mood swings. (ii) I am relaxed most of the time. (r) (iii) I get upset easily. (iv) I seldom feel blue. (r) (Sibley *et al.* 2011).

Items:

- I have frequent mood swings.
- I am relaxed most of the time (reversed).
- I get upset easily.
- I seldom feel blue (reversed).

Not Heterosexual Binary

Open-ended question, coded as binary (not heterosexual = 1) (Greaves *et al.* 2017).

Items:

- How would you describe your sexual orientation? (e.g., heterosexual, homosexual, straight, gay, lesbian, bisexual, etc.)

NZ Deprevation Index 2018

Numerical: (1-10) (Atkinson *et al.* 2019).

Items:

- New Zealand Deprivation - Decile Index - Using 2018 Census Data

NZSEI (Occupational Prestige Index)

This index uses the income, age, and education of a reference group, in this case, the 2013 New Zealand census, to calculate a score for each occupational group. Scores range from 10 (Lowest) to 90 (Highest). This list of index scores for occupational groups was used to assign each participant a NZSEI-13 score based on their occupation (Fahy *et al.* 2017).

Items:

- We assessed occupational prestige and status using the New Zealand Socio-economic Index 13 (NZSEI-13).

Openness

Mini-IPIP6 Openness to Experience dimension: (i) I have a vivid imagination. (ii) I have difficulty understanding abstract ideas. (r) (iii) I do not have a good imagination. (r) (iv) I am not interested in abstract ideas. (r) (Sibley *et al.* 2011).

Items:

- I have a vivid imagination.
- I have difficulty understanding abstract ideas (reversed).
- I do not have a good imagination (reversed).
- I am not interested in abstract ideas (reversed).

Parent (Binary)

Parents were coded as 1, while the others were coded as 0 (Sibley 2021).

Items:

- If you are a parent, in which year was your eldest child born?

Partner Binary

Coded as binary (has partner = 1) (Sibley 2021).

Items:

- What is your relationship status? (e.g., single, married, de-facto, civil union, widowed, living together, etc.)

Political Conservative

- Please rate how politically liberal versus conservative you see yourself as being.

Rural Gch 2018 Levels

"Participants residence locations were coded according to a five-level ordinal categorisation ranging from Urban to Rural." (Whitehead *et al.* 2023).

Items:

- High Urban Accessibility = 1, Medium Urban Accessibility = 2, Low Urban Accessibility = 3, Remote = 4, Very Remote = 5.

Right Wing Authoritarianism

Right Wing Authoritarianism was measured using the following items (Altemeyer 1996).

Items:

- It is always better to trust the judgment of the proper authorities in government and religion than to listen to the noisy rabble-rousers in our society who are trying to create doubt in people's minds.
- It would be best for everyone if the proper authorities censored magazines so that people could not get their hands on trashy and disgusting material.
- Our country will be destroyed some day if we do not smash the perversions eating away at our moral fibre and traditional beliefs.
- People should pay less attention to The Bible and other old traditional forms of religious guidance, and instead develop their own personal standards of what is moral and immoral.
- Atheists and others who have rebelled against established religions are no doubt every bit as good and virtuous as those who attend church regularly.
- Some of the best people in our country are those who are challenging our government, criticizing religion, and ignoring the "normal way" things are supposed to be done (reversed).

Sample Frame Opt-in (Binary)

Code string (Binary): (0 = No, 1 = Yes) (Sibley 2021).

Items:

- Participant was not randomly sampled from the New Zealand Electoral Roll.

Social Dominance Orientation

Social Dominance Orientation was measured using the following items (Sidanius and Pratto 1999).

Items:

- It is OK if some groups have more of a chance in life than others.
- Inferior groups should stay in their place.
- To get ahead in life, it is sometimes okay to step on other groups.
- We should have increased social equality (reversed).
- It would be good if groups could be equal (reversed).
- We should do what we can to equalise conditions for different groups (reversed).

Smoker (Binary)

Binary smoking indicator (0 = No, 1 = Yes) (Sibley 2021).

Items:

- Do you currently smoke tobacco cigarettes?

Outcome Variables

Alcohol Frequency

Participants could chose between the following responses: '(1 = Never - I don't drink, 2 = Monthly or less, 3 = Up to 4 times a month, 4 = Up to 3 times a week, 5 = 4 or more times a week, 6 = Don't know)' (Health 2013).

Items:

- "How often do you have a drink containing alcohol?"

Alcohol Intensity

Participants responded using an open-ended box (Health 2013).

Items:

- "How many drinks containing alcohol do you have on a typical day when drinking alcohol? (number of drinks on a typical day when drinking)"

Social Belonging

We assessed felt belongingness with three items adapted from the Sense of Belonging Instrument (Hagerty & Patusky, 1995): (1) "Know that people in my life accept and value me"; (2) "Feel like an outsider"; (3) "Know that people around me share my attitudes and beliefs". Participants responded on a scale from 1 (Very Inaccurate) to 7 (Very Accurate). The second item was reversely coded (Hagerty and Patusky 1995).

Items:

- Know that people in my life accept and value me.
- Feel like an outsider (reversed).
- Know that people around me share my attitudes and beliefs.

Body Satisfaction

Ordinal response (Stronge *et al.* 2015).

Items:

- I am satisfied with the appearance, size and shape of my body.

Forgiveness

We assessed participants' forgiveness using reversed scores of a the NZAVS "vengeful rumination scale." This scale contains three items, adapted from Caprara (1986) and Berry *et al.* (2005), and developed for NZAVS, ordinal response scale 1-7 (1 = Strongly Disagree to 7 = Strongly Agree) Caprara (1986).

Items:

- Sometimes I can't sleep because of thinking about past wrongs I have suffered.
- I can usually forgive and forget when someone does me wrong.
- I find myself regularly thinking about past times that I have been wronged.

Gratitude

Ordinal response scale 1 = Strongly Disagree to 7 = Strongly Agree (McCullough *et al.* 2002).

Items:

- I have much in my life to be thankful for.
- When I look at the world, I don't see much to be grateful for (reversed).
- I am grateful to a wide variety of people.

Body Mass Index

Based on participants indication of their height and weight we calculated the BMI by dividing the weight in kilograms by the square of the height in meters (Sibley 2021).

Items:

- What is your height? (metres)" and "What is your weight? (kg).

Fatigue

A single item assessing subjective fatigue over the past 30 days, scored on the same five-point frequency scale as the Kessler-6 items (0 = None of the Time to 4 = All of the Time). Introduced in the NZAVS at Time 5 (Sibley *et al.* 2020).

Items:

- During the last 30 days, how often did … you feel exhausted?

Sleep

Open ended response (Buysse *et al.* 1989).

Items:

- During the past month, on average, how many hours of actual sleep did you get per night?

Anxiety

The anxiety subscale of the Kessler-6 (K6) psychological distress screening scale, comprising three items that capture physiological arousal, cognitive load, and restlessness (alpha = 0.72) (Kessler *et al.* 2002).

Items:

- During the past 30 days, how often did…you feel nervous?
- During the past 30 days, how often did…you feel restless or fidgety?
- During the past 30 days, how often did…you feel that everything was an effort?

Depression

The depression subscale of the Kessler-6 (K6) psychological distress screening scale, comprising three items that assess depressed mood, hopelessness, and worthlessness (alpha = 0.86) (Kessler *et al.* 2002).

Items:

- During the past 30 days, how often did…you feel hopeless?
- During the past 30 days, how often did…you feel so depressed nothing could cheer you up?
- During the past 30 days, how often did…you feel worthless?

Life Satisfaction

(Diener *et al.* 1985).

Items:

- I am satisfied with my life.
- In most ways my life is close to ideal.

log Hours of Exercise

We took the natural log of the response + 1 (Sibley *et al.* 2011).

Items:

- Hours spent…exercising/physical activity.

Meaning: Purpose

A single item from the Meaning in Life Questionnaire (MLQ) assessing presence of purpose, scored on a seven-point (Steger *et al.* 2006).

Response scale: 1 = Strongly Disagree to 7 = Strongly Agree

Items:

- My life has a clear sense of purpose

Meaning: Sense

A single item from the Meaning in Life Questionnaire (MLQ) assessing presence of meaning, scored on a seven-point (Steger *et al.* 2006).

Response scale: 1 = Strongly Disagree to 7 = Strongly Agree

Items:

- I have a good sense of what makes my life meaningful.

Neighbourhood Belonging

(Sengupta *et al.* 2013).

Items:

- I feel a sense of community with others in my local neighbourhood.

Perfectionism

(Rice *et al.* 2014).

Items:

- Doing my best never seems to be enough.
- My performance rarely measures up to my standards.
- I am hardly ever satisfied with my performance.

Personal Well-Being Index

No information available for this variable.

Rumination

Ordinal responses: 0 = None of The Time, 1 = A little of The Time, 2 = Some of The Time, 3 = Most of The Time, 4 = All of The Time (Nolen-hoeksema and Morrow 1993).

Items:

- During the last 30 days, how often did…you have negative thoughts that repeated over and over?

Self Control

- In general, I have a lot of self-control
- I wish I had more self-discipline (reversed)

Self Esteem

(Rosenberg 1965).

Items:

- On the whole am satisfied with myself.
- Take a positive attitude toward myself.
- Am inclined to feel that I am a failure (reversed).

Sexual Satisfaction

Participants were asked to report their sexual orientation; ordinal response: 1 = Not satisfied to 7 = Very satisfied (Sibley 2021).

Items:

- How satisfied are you with your sex life?

Short Form Health

- In general, would you say your health is…

Social Support (Perceived)

- There are people I can depend on to help me if I really need it.
- There is no one I can turn to for guidance in times of stress (reversed).
- I know there are people I can turn to when I need help.

Sample Demographic Statistics

Table 1 presents sample demographic statistics.

Table 1: Demographic statistics for the New Zealand Attitudes and Values Study Cohort in the baseline wave (New Zealand Attitudes and Values Study time 10, spanning years 2018-2019).

	2018
	(N=46377)
**Age**	
Mean (SD)	48.6 (13.9)
Median [Min, Max]	51.0 [18.0, 99.0]
**Agreeableness**	
Mean (SD)	5.35 (0.988)
Median [Min, Max]	5.50 [1.00, 7.00]
Missing	400 (0.9%)
**Alcohol Frequency Weekly**	
Mean (SD)	1.76 (1.65)
Median [Min, Max]	1.00 [0, 4.50]
Missing	1747 (3.8%)
**Alcohol Intensity**	
Mean (SD)	2.15 (2.09)
Median [Min, Max]	2.00 [0, 15.0]
Missing	2751 (5.9%)
**Social Belonging**	
Mean (SD)	5.14 (1.08)
Median [Min, Max]	5.33 [1.00, 7.00]
Missing	397 (0.9%)
**Born in NZ**	
0	10041 (21.7%)
1	36184 (78.0%)
Missing	152 (0.3%)
**Conscientiousness**	
Mean (SD)	5.11 (1.06)
Median [Min, Max]	5.25 [1.00, 7.00]
Missing	392 (0.8%)
**Education Level**	
no_qualification	1177 (2.5%)
cert_1_to_4	16277 (35.1%)
cert_5_to_6	5821 (12.6%)
university	12311 (26.5%)
post_grad	5034 (10.9%)
masters	3857 (8.3%)
doctorate	1111 (2.4%)
Missing	789 (1.7%)
**Employed (binary)**	
0	9501 (20.5%)
1	36865 (79.5%)
Missing	11 (0.0%)

	2018
**Ethnicity**	
euro	36915 (79.6%)
maori	5311 (11.5%)
pacific	1109 (2.4%)
asian	2453 (5.3%)
Missing	589 (1.3%)
**Extraversion**	
Mean (SD)	3.91 (1.20)
Median [Min, Max]	4.00 [1.00, 7.00]
Missing	392 (0.8%)
**Hlth Disability Binary**	
Mean (SD)	0.225 (0.418)
Median [Min, Max]	0 [0, 1.00]
Missing	893 (1.9%)
**Hlth Fatigue**	
Mean (SD)	1.64 (1.09)
Median [Min, Max]	2.00 [0, 4.00]
Missing	500 (1.1%)
**Honesty Humility**	
Mean (SD)	5.41 (1.18)
Median [Min, Max]	5.50 [1.00, 7.00]
Missing	396 (0.9%)
**Kessler Latent Anxiety**	
Mean (SD)	1.21 (0.773)
Median [Min, Max]	1.00 [0, 4.00]
Missing	437 (0.9%)
**Kessler Latent Depression**	
Mean (SD)	0.585 (0.753)
Median [Min, Max]	0.333 [0, 4.00]
Missing	440 (0.9%)
**Log Hours Children**	
Mean (SD)	1.16 (1.61)
Median [Min, Max]	0 [0, 5.13]
Missing	1442 (3.1%)
**Log Hours Commute**	
Mean (SD)	1.50 (0.834)
Median [Min, Max]	1.61 [0, 4.39]
Missing	1442 (3.1%)
**Log Hours Exercise**	
Mean (SD)	1.54 (0.848)
Median [Min, Max]	1.61 [0, 4.39]
Missing	1442 (3.1%)
**Log Hours Housework**	
Mean (SD)	2.14 (0.781)
Median [Min, Max]	2.20 [0, 5.13]
Missing	1442 (3.1%)
**Log Household Income**	
Mean (SD)	11.4 (0.768)
Median [Min, Max]	11.5 [0.693, 14.9]
Missing	2940 (6.3%)
**Log Hours Community**	
Mean (SD)	0.343 (0.648)
Median [Min, Max]	0 [0, 4.80]

	2018
Missing	1442 (3.1%)
**Male (binary)**	
0	29121 (62.8%)
1	17148 (37.0%)
Missing	108 (0.2%)
**Neuroticism**	
Mean (SD)	3.49 (1.15)
Median [Min, Max]	3.50 [1.00, 7.00]
Missing	402 (0.9%)
**Not Heterosexual Binary**	
0	42163 (90.9%)
1	3039 (6.6%)
Missing	1175 (2.5%)
**NZ Deprevation Index 2018**	
Mean (SD)	4.77 (2.73)
Median [Min, Max]	4.00 [1.00, 10.0]
Missing	308 (0.7%)
**NZSEI (Occupational Prestige Index)**	
Mean (SD)	54.1 (16.5)
Median [Min, Max]	54.0 [10.0, 90.0]
Missing	346 (0.7%)
**Openness**	
Mean (SD)	4.96 (1.12)
Median [Min, Max]	5.00 [1.00, 7.00]
Missing	394 (0.8%)
**Parent (binary)**	
0	13541 (29.2%)
1	32836 (70.8%)
**Partner Binary**	
Mean (SD)	0.750 (0.433)
Median [Min, Max]	1.00 [0, 1.00]
Missing	535 (1.2%)
**Political Conservative**	
Mean (SD)	3.59 (1.38)
Median [Min, Max]	4.00 [1.00, 7.00]
Missing	1902 (4.1%)
**Rural Gch 2018 Levels**	
High Urban Accessibility	28607 (61.7%)
Medium Urban Accessibility	8695 (18.7%)
Low Urban Accessibility	5639 (12.2%)
Remote	2581 (5.6%)
Very Remote	549 (1.2%)
Missing	306 (0.7%)
**Right Wing Authoritarianism**	
Mean (SD)	3.28 (1.15)
Median [Min, Max]	3.20 [1.00, 7.00]
Missing	7 (0.0%)
**Sample Frame Opt - In (binary)**	
0	45001 (97.0%)
1	1376 (3.0%)
**Social Dominance Orientation**	
Mean (SD)	2.32 (0.961)
Median [Min, Max]	2.17 [1.00, 7.00]

	2018
Missing	1 (0.0%)
**Short Form Health**	
Mean (SD)	5.04 (1.17)
Median [Min, Max]	5.00 [1.00, 7.00]
Missing	9 (0.0%)
**Smoker (binary)**	
0	41895 (90.3%)
1	3297 (7.1%)
Missing	1185 (2.6%)
**Hours Children**	
Mean (SD)	14.0 (32.3)
Median [Min, Max]	0 [0, 168]
Missing	1442 (3.1%)
**Hours Commute**	
Mean (SD)	5.29 (6.40)
Median [Min, Max]	4.00 [0, 80.0]
Missing	1442 (3.1%)
**Hours Exercise**	
Mean (SD)	5.78 (7.70)
Median [Min, Max]	4.00 [0, 80.0]
Missing	1442 (3.1%)
**Hours Housework**	
Mean (SD)	10.3 (10.1)
Median [Min, Max]	8.00 [0, 168]
Missing	1442 (3.1%)
**Hours Community**	
Mean (SD)	0.937 (2.87)
Median [Min, Max]	0 [0, 120]
Missing	1442 (3.1%)
**Household Income**	
Mean (SD)	115000 (92100)
Median [Min, Max]	100000 [1.00, 3010000]
Missing	2940 (6.3%)

Exposure Variable: Religious Service Attendance

Table 2 describes religious service attendance at baseline and during the exposure waves. Attendance was not collected in parts of wave 12 (2020-2021) and wave 13 (2021-2022). Because this exposure is central to our research question, we retained it and managed the missingness as follows: When a later wave recorded attendance, we filled the earlier gap with the most recent observed value and added an imputed flag (carry-forward imputation). If no later attendance data were available, we treated the participant as censored from that point onward and applied inverse-probability-of-censoring weights, as outlined in *Method: Handling of missing data*.

Including the exposure despite missingness preserves sample size, maintains comparability with previous New Zealand Attitudes and Values Study analyses, and avoids discarding information that could clarify causal pathways. The carry-forward step may attenuate effects by introducing random measurement error, but it is improbable that it would inflate them. Thus, any detected causal effects should be viewed as conservative estimates.

Table 2: Sample statistics for the exposure variable (religious service attendance) across New Zealand Attitudes and Values Study time-10 through time-14 cohort waves (2018–2022). Attendance was not recorded in 2020 and was only partially recorded in 2021, resulting in high levels of missingness. For this reason, we cannot estimate the effects of interventions on religious attendance in those two waves.

	2018	2019	2020	2021	2022	2023
	(N=46377)	(N=46377)	(N=46377)	(N=46377)	(N=46377)	(N=46377)
**Religious Attendance**						
Mean (SD)	0.756 (2.71)	0.686 (2.55)	NA (NA)	0.115 (1.06)	0.598 (2.16)	0.653 (2.40)
Median [Min, Max]	0 [0, 60.0]	0 [0, 60.0]	NA [NA, NA]	0 [0, 30.0]	0 [0, 30.0]	0 [0, 30.0]
Missing	0 (0%)	12671 (27.3%)	46377 (100%)	26976 (58.2%)	23240 (50.1%)	25369 (54.7%)

Outcome Variables

Table 3: Outcome variables measured at baseline (New Zealand Attitudes and Values Study time 10, years 2018-2019, and time 15, years 2023-2024).

	2018	2023
	(N=46377)	(N=46377)
**Alcohol Frequency (weekly)**		
Mean (SD)	1.76 (1.65)	1.64 (1.63)
Median [Min, Max]	1.00 [0, 4.50]	1.00 [0, 4.50]
Missing	1747 (3.8%)	25576 (55.1%)
**Alcohol Intensity**		
Mean (SD)	2.15 (2.09)	1.99 (1.75)
Median [Min, Max]	2.00 [0, 15.0]	2.00 [0, 15.0]
Missing	2751 (5.9%)	27939 (60.2%)
**Body Mass Index**		
Mean (SD)	27.2 (5.87)	27.8 (6.07)
Median [Min, Max]	26.2 [12.3, 73.6]	26.7 [13.2, 87.4]
Missing	1171 (2.5%)	25123 (54.2%)
**Sleep**		
Mean (SD)	6.94 (1.13)	6.93 (1.12)
Median [Min, Max]	7.00 [2.50, 16.0]	7.00 [2.00, 16.0]
Missing	2365 (5.1%)	26025 (56.1%)
**Hours of Exercise**		
Mean (SD)	5.78 (7.70)	6.42 (7.29)
Median [Min, Max]	4.00 [0, 80.0]	5.00 [0, 80.0]
Missing	1442 (3.1%)	25897 (55.8%)
**Log Hours Exercise**		
Mean (SD)	1.54 (0.848)	1.67 (0.830)
Median [Min, Max]	1.61 [0, 4.39]	1.79 [0, 4.39]
Missing	1442 (3.1%)	25897 (55.8%)
**Short Form Health**		
Mean (SD)	5.04 (1.17)	4.84 (1.17)
Median [Min, Max]	5.00 [1.00, 7.00]	5.00 [1.00, 7.00]
Missing	9 (0.0%)	25080 (54.1%)

Table 4: Table 4 presents single-world intervention graphs showing time-fixed and time-varying sources of bias in our six waves (baseline, four exposure waves, followed by the outcome wave). Time-fixed confounders are included in the baseline wave. Time-varying confounders are included in each of the four treatment waves (abbreviated here by '...' to declutter the graph). When there are multiple exposure waves, identifying causal effects requires adjustment for time-varying confounders (Bulbulia 2024c; Richardson and Robins 2013; Robins and Hernan 2008).

Single World Identification Graphs

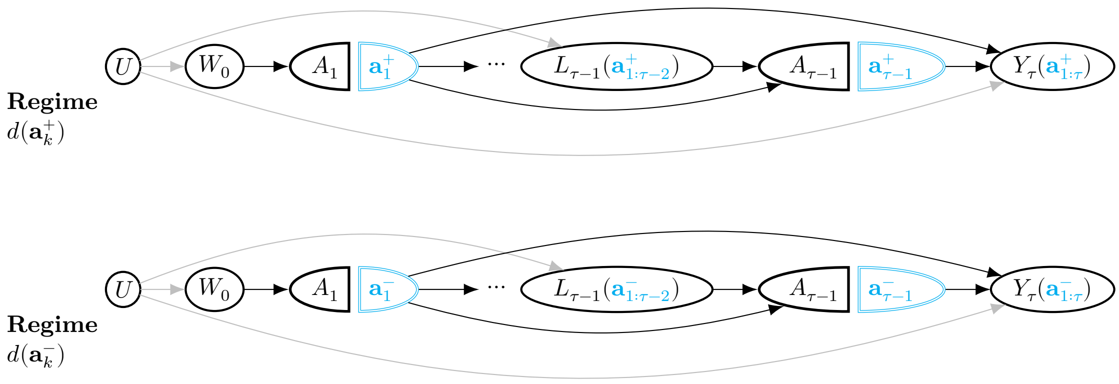

Key: Y_τ represents the outcome at the end of study, A_k the treatment at time $K = k$, U unmeasured confounders, W_0, confounders measured at baseline, and L_k time-varying confounder. We define $H_k = (\bar{A}_{k-1}, \bar{L}_k, W_0)$ We assume $A_k \coprod Y_\tau \mid H_k$. Investigators aim to determine if $Y(\bar{a}) \coprod \bar{A}_k \mid H_k$, with A_k as a sequence of treatments and indicating past treatment and confounder histories. Counterfactual contrasts are predefined for static and dynamic treatments under a regime specified by $d(A_k)$. $\longrightarrow$ indicates assumed causal paths; $\longrightarrow$ denotes closed confounding paths after conditioning on measured covariates and fixing interventions. Each Single World Intervention Graph evaluates d-separation separately for each intervention to be contrasted. We split each node into random and deterministic components, with descendants labelled as potential outcomes under treatment. Here, we contrast the regime $d(\mathbf{a}_k^+)$ with the regime $d(\mathbf{a}_k^-)$.

Supplement S3: Confounding Control

For confounding control at baseline, we employ a modified disjunctive cause criterion (VanderWeele 2019), a strategy for selecting confounders that includes all common causes of treatment and outcome while excluding variables that affect only treatment, which involves:

1. Identifying all common causes of both the treatment and outcomes.
2. Excluding instrumental variables that affect the exposure but not the outcome.
3. Including proxies for unmeasured confounders affecting both exposure and outcome.
4. Controlling for baseline exposure and baseline outcome, serving as proxies for unmeasured common causes (VanderWeele *et al.* 2020).

Additionally, we control for time-varying confounders at each exposure wave (Bulbulia 2024c; Richardson and Robins 2013; Robins and Hernan 2008).

The covariates included for confounding control are described in Rosa *et al.* (2024).

Time-varying confounders

Table 5 presents the subset of 62 baseline confounders included as time-varying confounders ($n = 32$, including disability, which is entered contemporaneously rather than lagged; see table note). Time-varying confounders were entered at each exposure wave as lagged values (measured at wave $t-1$, used to adjust the exposure at t), ensuring temporal priority and avoiding blocking post-exposure pathways (Bulbulia 2024b; Lu *et al.* 2022; McElreath 2020; Montgomery *et al.* 2018). The sole exception is disability, which we include contemporaneously with exposure at t on the theory that disability is not a plausible effect of religious attendance in any given wave yet may confound both attendance and multidimensional well-being. Measure wording and coding details appear in Supplement S2.

Table 5: ♠: *Concurrent (t) and lagged ($t-1$). All other variables are lagged one wave.*

Measure	Brief description
Alcohol frequency	Self-reported frequency (categorical, transformed to weekly).
Alcohol intensity	Typical number of drinks per drinking day (open response).
Employment status	Currently employed (yes/no).
Household income (log)	Natural log of annual before-tax household income.
New Zealand Deprivation Index (2018) decile	Census-linked area-level deprivation decile (1–10).
New Zealand Socioeconomic Index (2013) occupational prestige	Socioeconomic index for occupational status (continuous).
Parent status	Has children (yes/no).
Partner status	In a romantic partnership (yes/no).
Rurality	Urban-rural accessibility classification (5 levels, Geographic Classification for Health).
Short-Form health	General self-rated health (short-form scale).
Fatigue	Frequency/level of fatigue (short health scale).
Body mass index	Calculated from self-reported height and weight.
Sleep hours	Average nightly sleep hours (self-report).
Hours of exercise (log)	Natural log of weekly exercise hours +1.
Anxiety	Latent factor from Kessler distress items indexing anxiety.
Depression	Latent factor from Kessler distress items indexing depression.
Life satisfaction	Global life satisfaction rating.
Social belonging	Three-item scale: acceptance, outsider (rev.), shared attitudes.
Neighbourhood community	Perceived neighbourhood engagement/cohesion.
Perceived social support	Availability of supportive others.
Body satisfaction	Satisfaction with body/appearance.
Forgiveness	Dispositional/enacted forgiveness.
Gratitude	Dispositional gratitude.
Meaning: purpose	Perceived life purpose/goal direction.
Meaning: sense	Sense of meaning/coherence in life.
Perfectionism	Tendency toward perfectionistic standards/concerns.
Rumination	Repetitive negative thinking/ruminative tendency.
Self-control	Perceived ability to regulate impulses/behaviour.
Self-esteem	Global self-worth/self-evaluation.
Sexual satisfaction	Satisfaction with sexual life.
Personal well-being index	Multi-item subjective well-being index across domains.
Disability (6+ months)♠	Limiting condition ≥ 6 months (yes/no).

Notation and data structure

We now formalise the data structure described above. Consider n individuals indexed by $i = 1, \dots n$. Let $\{O_i\}_{i=1}^{n}$ be i.i.d. copies of a generic observed data vector O. We index baseline observations at $t = 0$ and subsequent study

waves at $t = 1, \ldots, 5$. Let W_0 denote baseline covariates measured at wave 0 (including baseline exposure and all baseline outcomes). For $t = 1, \ldots, 4$, let L_t denote time-varying covariates (including lagged outcomes) measured before the exposure measurement at wave t, and let A_t denote religious service attendance (monthly frequency) at wave t. Let R_t indicate remaining under observation through wave $t + 1$; specifically, R_0 indicates retention from baseline to wave 1. Variables at time t are observed only among those with $\bar{R}_{t-1} = 1$ (where bars denote cumulative histories). The temporal ordering is $L_t \to A_t \to R_t \to L_{t+1}$.

We assume observed data arise from a data-generating process:

$$O = (W_0, R_0, L_1, A_1, R_1, L_2, A_2, R_2, L_3, A_3, R_3, L_4, A_4, R_4, Y_5),$$

with the understanding that post-censoring variables are undefined (set to missing/NA). We define the history available to treatment at time t by $\mathcal{H}_t = (W_0, \bar{L}_t, \bar{A}_{t-1}, \bar{R}_{t-1})$ for $t = 1, \ldots, 4$. Let $g_t(a \mid \mathcal{H}_t)$ denote the conditional mass/density of A_t given $\mathcal{H}_t$.

Causal assumptions

Causal inference rests on three identification assumptions (Bulbulia 2024b; Morgan and Winship 2014; VanderWeele 2015).

Consistency and no interference. Consistency requires that if an individual's exposure path equals the policy-modified path, then their observed outcome equals the corresponding potential outcome. If an individual's observed path satisfies $A_t = d_t(A_t, \mathcal{H}_t)$ for all t and $\bar{R}_4 = 1$, then the observed outcome equals the potential outcome under policy $\bar{d}$, $Y_5 = Y^{\bar{d}}$. Our longitudinal modified treatment policies modify only the current month's attendance and leave all else unchanged. No interference assumes one person's attendance does not affect another's outcome except through measured variables (VanderWeele 2009). Congregation-level or network spillovers would violate this; we treat such effects as negligible for identification but note that they are possible in social settings. There is inherent ambiguity in the 'attendance' intervention because ritual attendance takes multiple forms; our results average across these forms in the target population. Although we may consistently estimate causal effects, ambiguity in attendance may make it unclear what intervention is proposed (Hernan and Robins 2020; VanderWeele 2012).

Sequential exchangeability (including censoring). Conditional on the measured history $\mathcal{H}_t = (W_0, \bar{L}_t, \bar{A}_{t-1}, \bar{R}_{t-1})$, treatment and censoring at time t are assumed independent of the future potential outcome under the policy. Formally, with $\mathcal{H}_0 = (W_0)$,

$$Y^{\bar{d}} \perp\!\!\!\perp R_0 \mid \mathcal{H}_0,$$

and for each $t = 1, \ldots, 4$,

$$Y^{\bar{d}} \perp\!\!\!\perp A_t \mid \mathcal{H}_t,\ \bar{R}_{t-1} = 1, \qquad Y^{\bar{d}} \perp\!\!\!\perp R_t \mid \mathcal{H}_t, A_t,\ \bar{R}_{t-1} = 1.$$

We make this assumption more plausible by adjusting for (i) rich baseline covariates, (ii) all baseline outcomes and baseline exposure, and (iii) time-varying confounders (including lagged outcomes) at each wave. Residual bias may arise from unmeasured or mismeasured time-varying confounders; we report E-values as sensitivity analysis.

Policy-support positivity (including censoring). The positivity requirements differ between our deterministic and probabilistic interventions.

For **deterministic policies** (weekly, monthly, zero, identity), we require policy-support positivity: for any covariate history with positive probability, every exposure value that a policy maps into must occur with positive probability. Let $g_t(a \mid \mathcal{H}_t)$ denote the observational density of treatment assignment at time t given history $\mathcal{H}_t$. For each t and every $\mathcal{H}_t$ with $\bar{R}_{t-1} = 1$, whenever $g_t(a \mid \mathcal{H}_t) > 0$ it also holds that $g_t(d_t(a, \mathcal{H}_t) \mid \mathcal{H}_t) > 0$. We assess evidence for practical positivity by inspecting density-ratio distributions at each wave (Supplement S5).

For **incremental propensity score interventions**, positivity is satisfied by construction because the stochastic assignment mechanism ensures that both exposure values (0 and 1) occur with positive probability for all

individuals at each time point, regardless of their covariate history. Under the marginal incremental propensity score intervention with parameter δ, every individual has a positive probability of receiving either exposure value through the uniform random draw ϵ_t, eliminating covariate-specific support violations that affect deterministic policies. Nevertheless, very strong shifts can strain *practical* positivity, as documented in the main text and Supplement S5.

For **censoring**, positivity requires that remaining under observation has positive probability conditional on the past:

$$\Pr(R_0 = 1 \mid W_0) > 0 \quad \text{and} \quad \Pr(R_t = 1 \mid \mathcal{H}_t, A_t, \bar{R}_{t-1} = 1) > 0 \text{ for } t = 1, \dots, 4.$$

Deterministic policies (longitudinal modified treatment policies)

We specify causal questions using longitudinal modified treatment policies that deterministically modify observed attendance while preserving observed support (Díaz *et al.* 2021). A policy is a sequence $\overline{d} = \{d_1, \dots, d_4\}$ of maps that set the exposure under the policy. Policies may depend on measured history. We write the policy-induced history as

$$\mathcal{H}_t^{\overline{d}} \equiv (W_0,\, \bar{L}_t^{\overline{d}},\, \bar{A}_{t-1}^{\overline{d}},\, \bar{R}_{t-1}^{\overline{d}}),$$

where superscripts denote potential values under the policy-induced exposure history. The counterfactual exposure under the policy at time t is

$$A_t^{\overline{d}} \;:=\; d_t(A_t(\overline{d}),\, \mathcal{H}_t^{\overline{d}}).$$

In our analyses, the policy is history-independent and modifies only the current exposure, so $A_t^{\overline{d}} = d_t(A_t(\overline{d}))$. We consider four deterministic policies:

At least weekly attendance sets attendance to four or more times per month:

$$d_t^{\text{weekly}}(a) = \begin{cases} 4, & \text{if } a < 4, \\ a, & \text{otherwise.} \end{cases}$$

At least monthly attendance sets attendance to one or more times per month:

$$d_t^{\text{monthly}}(a) = \begin{cases} 1, & \text{if } a < 1, \\ a, & \text{otherwise.} \end{cases}$$

No attendance sets attendance to zero at every exposure time:

$$d_t^{\text{zero}}(a) = 0 \quad \text{for all } a.$$

Identity policy leaves attendance unchanged:

$$d_t^{\text{identity}}(a) = a \quad \text{for all } a.$$

All policies are coupled with a hypothetical no-censoring intervention ($R_t^{\overline{d}} \equiv 1$ for $t = 0, \dots, 4$), defining causal estimands for the full baseline cohort by eliminating loss to follow-up in the counterfactual setting.

Incremental propensity score interventions (probabilistic policies)

Whereas deterministic policies set exposure values with certainty, incremental propensity score interventions are probabilistic regimes that modify the probability of treatment assignment while leaving realised values stochastic. For a multiplicative factor $\delta > 1$, the intervention to increase initiation probability is:

$$d_t^{(\delta)}(a_t, \epsilon_t) = \begin{cases} a_t, & \text{if } \epsilon_t < 1/\delta, \\ 1, & \text{otherwise,} \end{cases}$$

where $\epsilon_t \sim \text{Uniform}(0,1)$ is an independent random draw for each individual at time t. When $\delta = 1$, this reduces to the identity intervention. When $\delta > 1$, each individual has probability $1/\delta$ of maintaining their observed exposure and probability $1 - 1/\delta$ of being assigned to initiate attendance.

For completeness, the intervention to decrease initiation probability (with $\delta \in (0,1]$) is:

$$d_t^{(\delta)}(a_t, \epsilon_t) = \begin{cases} a_t, & \text{if } \epsilon_t < \delta, \\ 0, & \text{otherwise.} \end{cases}$$

We do not consider downshift interventions in this study.

Let $p = \Pr(A_t = 1)$ be the natural marginal initiation probability. The counterfactual probability becomes $p' = 1-(1-p)/\delta$. Based on empirical transition tables (Supplement S4), natural initiation rates are approximately 3.0% at the first exposure wave (845 initiations out of 28,225 non-attenders) and 2.1% at the fourth exposure wave (355 initiations out of 16,986 non-attenders). We report effects for $\delta \in \{2, 5, 10\}$. As with deterministic policies, incremental propensity score interventions are paired with a hypothetical no-censoring intervention ($R_t^{(\delta)} \equiv 1$).

The `lmtp` package implements these interventions via the `ipsi()` function. Because the baseline observation is not intervened upon (it is included for confounding control and to adjust for censoring into the first exposure wave), we required custom code:

```
treatment_ipsi_02 <- local({
  delta_ipsi_02 <- 0.5 # 1/delta for the delta = 2 policy
  d_ipsi_02 <- function(data, trt) {
    eps <- runif(nrow(data), 0, 1)
    ifelse(eps < delta_ipsi_02, data[[trt]], 1)
  }
  shift_fn <- d_ipsi_02
  function(data, trt) {
    if (trt == "t0_religion_church_binary") {
      return(data[[trt]]) # baseline value unchanged
    }
    if (trt == "t1_religion_church_binary") {
      return(shift_fn(data, trt)) # shift at first exposure wave
    }
    if (trt == "t4_religion_church_binary") {
      return(shift_fn(data, trt)) # shift at fourth exposure wave
    }
  }
})
```

Estimands and estimation

Let $Y^{\bar{d}}$ denote the potential outcome under policy $\bar{d}$, defined under the hypothetical no-censoring intervention $R_t^{\bar{d}} \equiv 1$ for $t = 0, \ldots, 4$. Our target marginal means are:

$$\psi_{\text{weekly}} = \mathbb{E}\left[Y^{\bar{d}^{\text{weekly}}}\right], \quad \psi_{\text{monthly}} = \mathbb{E}\left[Y^{\bar{d}^{\text{monthly}}}\right], \quad \psi_{\text{zero}} = \mathbb{E}\left[Y^{\bar{d}^{\text{zero}}}\right], \quad \psi_{\text{identity}} = \mathbb{E}\left[Y^{\bar{d}^{\text{identity}}}\right],$$

with primary contrasts such as $\psi_{\text{identity}} - \psi_{\text{zero}}$ for the deterministic shift-down policy and $\mathbb{E}\left[Y^{(\delta)}\right] - \mathbb{E}\left[Y^{(1)}\right]$ for incremental propensity score interventions. Contrasts involving upward deterministic shifts fail positivity and are reported in Supplement S6 Part B and Supplement S8 for completeness but should not be interpreted as causal estimates because they fail positivity.

Conceptually, this framework emulates a sequential trial in which participants are randomised at baseline to a policy arm applied through the wave prior to the end of study. These are per-protocol (de jure) policy effects for the full baseline cohort, with attrition and non-response handled via inverse probability of censoring weights. To recover the per-protocol effect of a prescribed intervention, experiments must likewise formulate an explicit approach, as randomisation only guarantees the intention-to-treat effect. Such an effect will depart from the per-protocol effect whenever there is attrition or individuals do not follow assigned treatments (Bulbulia 2024d).

Statistical estimator

We estimate causal effects of time-varying treatment regimes using the sequentially doubly robust (SDR) estimator in the `lmtp` package (Díaz *et al.* 2021; Hoffman *et al.* 2024; Williams and Díaz 2021). The SDR estimator belongs to the class of targeted learning methods that combine outcome regression with propensity score models to achieve double robustness (Van Der Laan and Rose 2011, 2018). It proceeds in two main steps. First, we use machine-learning ensembles to flexibly model (i) the conditional density of exposure given history, (ii) the conditional density of censoring given history and exposure, and (iii) the conditional expectation of the outcome given history and exposure at each wave. These nuisance functions are estimated using `SuperLearner` (Polley *et al.* 2023b), which combines multiple candidate algorithms to optimise predictive performance. `SuperLearner` splits the data into training and validation folds, fits each candidate learner on the training data, evaluates predictions on held-out validation data, and selects a weighted combination that minimises cross-validated prediction error. We include three base learners: `SL.ranger` (random forests for non-linear relationships and interactions), `SL.glmnet` (elastic-net regularised regression for high-dimensional covariates), and `SL.xgboost` (gradient boosting for complex patterns) (Chen *et al.* 2023; Polley *et al.* 2023a; Wright and Ziegler 2017). We also include a simple mean learner for validation, under the expectation that the base learners perform better than simply averaging. Supplement S7 reports learner performance and ensemble weights for results that satisfy positivity. Second, SDR targets these initial estimates by constructing a sequence of bias-corrected estimating equations that work backwards in time from the outcome. At each wave t, the estimator updates the outcome regression by incorporating inverse-probability weights derived from the treatment and censoring densities, yielding an efficient, doubly robust estimate of the marginal mean under the policy (Díaz *et al.* 2021).

Cross-validation. We use five-fold cross-fitting throughout the estimation procedure. Cross-fitting ensures that predictions used in the targeting step are generated from models never trained on the same observations, eliminating overfitting bias (Chernozhukov *et al.* 2018). Each observation's predicted values are derived exclusively from models fitted to different data, preserving the integrity of the doubly robust estimating equations. This separation is critical for valid inference when using flexible machine learning (Kennedy 2019; Van Der Laan and Rose 2011).

Double robustness and multiply robustness. The SDR estimator is doubly robust: it remains consistent if either (i) the sequence of outcome regressions is correct, or (ii) both the treatment and censoring density models are correct (Díaz *et al.* 2021). When treatments and censoring are repeated across multiple waves, the estimator achieves *multiply robust* protection: misspecification at one time point can be compensated by correct specification at others (Díaz *et al.* 2023; Hoffman *et al.* 2024). Influence-function-based standard errors account for estimation uncertainty in the nuisance functions (Williams and Díaz 2021).

Attrition. Inverse-probability-of-censoring weights (IPCW) are estimated within the SDR framework using the same machine-learning ensemble. By modelling retention as a time-varying process conditional on observed history, IPCW adjusts for selection bias under the assumption that censoring is conditionally independent of potential outcomes given measured covariates (Hoffman *et al.* 2024; Robins *et al.* 1999).

We batch estimated `lmtp` models, assessed positivity, and generated graphs, tables, and output reports using the `margot` package (Bulbulia 2024a). For comprehensive explanations of targeted learning with longitudinal modified treatment policies, including incremental propensity score interventions, see Hoffman *et al.* (2022); Hoffman *et al.* (2024); Díaz *et al.* (2021).

Missing data

We address missingness at three levels. First, we impute missing baseline covariates using predictive mean matching (mice) with single imputation restricted to baseline variables, following recommendations for outcome-wide designs (Van Buuren 2018; Zhang *et al.* 2023). Baseline missingness rates are low (1.3550332). Because single imputation understates uncertainty, we include missingness indicators so learners can condition on missingness patterns (Díaz *et al.* 2021; Hoffman *et al.* 2024).

Second, for time-varying covariates (but not exposures), we carry forward the last observation when a future value is available, include a missingness indicator, and leave exposure missingness to be handled by censoring weights (Burton and Altman 2004; Hoffman *et al.* 2024). Religious service attendance was not asked in the second exposure wave and was largely unobserved in the third exposure wave. For this reason, we do not estimate effects for shifting attendance at the second or third exposure wave in the six-wave studies. The three-wave studies are not subject to this administrative gap. The six-wave propensity score interventions are restricted to the baseline population of non-attenders. No missing exposure indicator is imputed in these analyses; subsequent missingness is handled entirely via censoring weights, and there are no administrative gaps in the attendance record.

Third, we treat attrition as censoring and estimate inverse-probability-of-censoring weights using non-parametric ensembles within the longitudinal modified treatment policy framework (Williams and Díaz 2023). We target inferences for the baseline cohort had they remained in the study, not for the selected sample that happens to remain at the end.

Data structure

Sensitivity analysis

We performed sensitivity analyses using the E-value (Linden *et al.* 2020; VanderWeele and Ding 2017). The E-value represents the minimum strength of association, on the risk-ratio scale, that an unmeasured confounder would need to induce with *both* the exposure and outcome, after adjusting for measured covariates, to explain away a result (Linden *et al.* 2020; VanderWeele *et al.* 2020). Even when confidence intervals do not cross zero, we consider E-values below 1.10 insufficiently robust to unmeasured confounding. We correct for multiple comparisons using the Bonferroni correction, and E-values are computed after family-wise error correction ($\alpha = 0.05$, following recommendations in VanderWeele *et al.* (2020)).

Table 6: **Data structure for six-wave studies.** W denotes baseline covariates ($n = 61$, i.e. 62 minus the baseline exposure variable). The baseline covariate set includes measures for each of the 24 outcomes. We target the effects of initiation into religious service among baseline non-attenders (hence we do not include the religious service attendance measure at $t = 0$). R_t indicates remaining under observation between wave t and the next fully observed exposure wave (for example, $R_0 = 0$ indicates loss to follow-up at the first exposure wave; $R_1 = 0$ indicates loss to follow-up at the fourth exposure wave (because waves 2 and 3 are not intervention waves, R_1 bridges waves 1 and 4); $R_4 = 0$ denotes loss to follow-up at the final wave). The exposures (and hence censoring) are ignored at the second and third exposure waves, where censoring in the first exposure wave tracks attrition any time up to the fourth exposure wave. All regime targets are defined with $\bar{R} \equiv 1$ and inverse-probability-of-censoring weighting is used to recover causal effects for the baseline cohort population. For individuals observed at A_4 (i.e. if $R_1 = 1$), we include indicators at L_2, L_3 to better adjust for time-varying confounding during the intervening period, in case L_4 indicators do not entirely block all backdoor paths. If a participant was lost to follow-up between A_1 and A_4, all future data were removed at A_1, with inverse-probability-of-censoring weighting used to adjust for selection bias. Interventions cannot be assessed for the second and third exposure waves because the attendance question was not asked.

	**Baseline**	**Period 1**			**Period 2**			**Period 3**			**Period 4**			**End**	**Outcome**
**ID**	**W**	$\mathbf{R_0}$	$\mathbf{L_1}$	$\mathbf{A_1}$	$\mathbf{R_1}$	$\mathbf{L_2}$	$\mathbf{A_2}$	$\mathbf{R_2}$	$\mathbf{L_3}$	$\mathbf{A_3}$	$\mathbf{R_3}$	$\mathbf{L_4}$	$\mathbf{A_4}$	$\mathbf{R_4}$	**Y**
1	0.4, -0.2	1	0.2, 0.1	1.2	1	0.3, -0.2	-1.5	1	0.4, -0.3	0.7	1	0.5, -0.8	0.81	1	-0.88
2	-1.0, -0.9	1	-0.5, 0.3	0.4	1	0.8, 0.1	0.9	1	0.2, -0.8	0.9	1	-0.4, 0.6	0.5	1	0.42
⋮	⋮	⋮	⋮	⋮	⋮	⋮	⋮	⋮	⋮	⋮	⋮	⋮	⋮	⋮	⋮
N	2.4, 0.3	0	–	–	–	–	–	–	–	–	–	–	–	–	–

Supplement S4: Year-on-year transitions in religious service attendance

The following graphs show the magnitude of shifts for the three deterministic shift interventions we consider in this study (the identity (no-shift) policy is omitted because it leaves attendance unchanged.)

These transition matrices capture shifts in states between consecutive waves. Each cell shows the count of individuals transitioning from one state to another. Rows are the initial state (From), columns the subsequent state (To). **Diagonal entries** (in **bold**) mark those who stayed in the same state.

Table 7: Transition Matrix From Wave 2018 to Wave 2019

From / To	State 1	State 2	State 3	State 4	State 5	State 6	State 7	State 8	State 9	Total
State 1	27380	405	174	71	125	26	13	8	23	28225
State 2	658	257	85	44	46	5	2	3	3	1103
State 3	238	104	204	104	94	12	12	2	12	782
State 4	114	54	110	181	172	18	8	4	11	672
State 5	155	71	126	202	902	123	64	16	49	1708
State 6	24	7	17	17	145	65	25	7	9	316
State 7	15	5	13	17	83	22	36	5	19	215
State 8	9	0	6	3	16	6	9	8	8	65
State 9	21	5	9	6	53	14	22	10	95	235

Table 8: Transition Matrix From Wave 2021 to Wave 2022 (recall wave 2020 lacked the attendance measure, and wave 2021 was randomly incomplete (the question was omitted for most of that wave).

From / To	State 1	State 2	State 3	State 4	State 5	State 6	State 7	State 8	State 9	Total
State 1	17190	270	115	56	76	10	13	7	5	17742
State 2	339	120	57	25	28	4	3	0	2	578
State 3	158	77	109	49	59	4	6	3	5	470
State 4	86	43	73	95	91	10	7	1	5	411
State 5	140	41	107	130	490	51	22	8	19	1008
State 6	25	7	6	23	92	33	9	2	7	204
State 7	11	3	4	13	36	19	21	2	8	117
State 8	6	4	1	2	14	4	4	2	2	39
State 9	20	4	5	8	51	13	9	3	42	155

Each New Zealand Attitudes and Values Study wave covers 1 October of the stated year to 30 September of the next (for example, the baseline wave spans 1 Oct 2018-30 Sep 2019). The four transition matrices track movement across nine ordered attendance categories: from State 0 (‘Never’) to State 8, the highest on our monthly attendance scale. Rows give each participant’s category at wave t; columns give their category at wave $t+1$. Diagonal cells show counts that did not change; off-diagonals show shifts. The right-hand ‘Total’ column reports the number of respondents in each category at wave t.

Transitions among baseline non-attenders

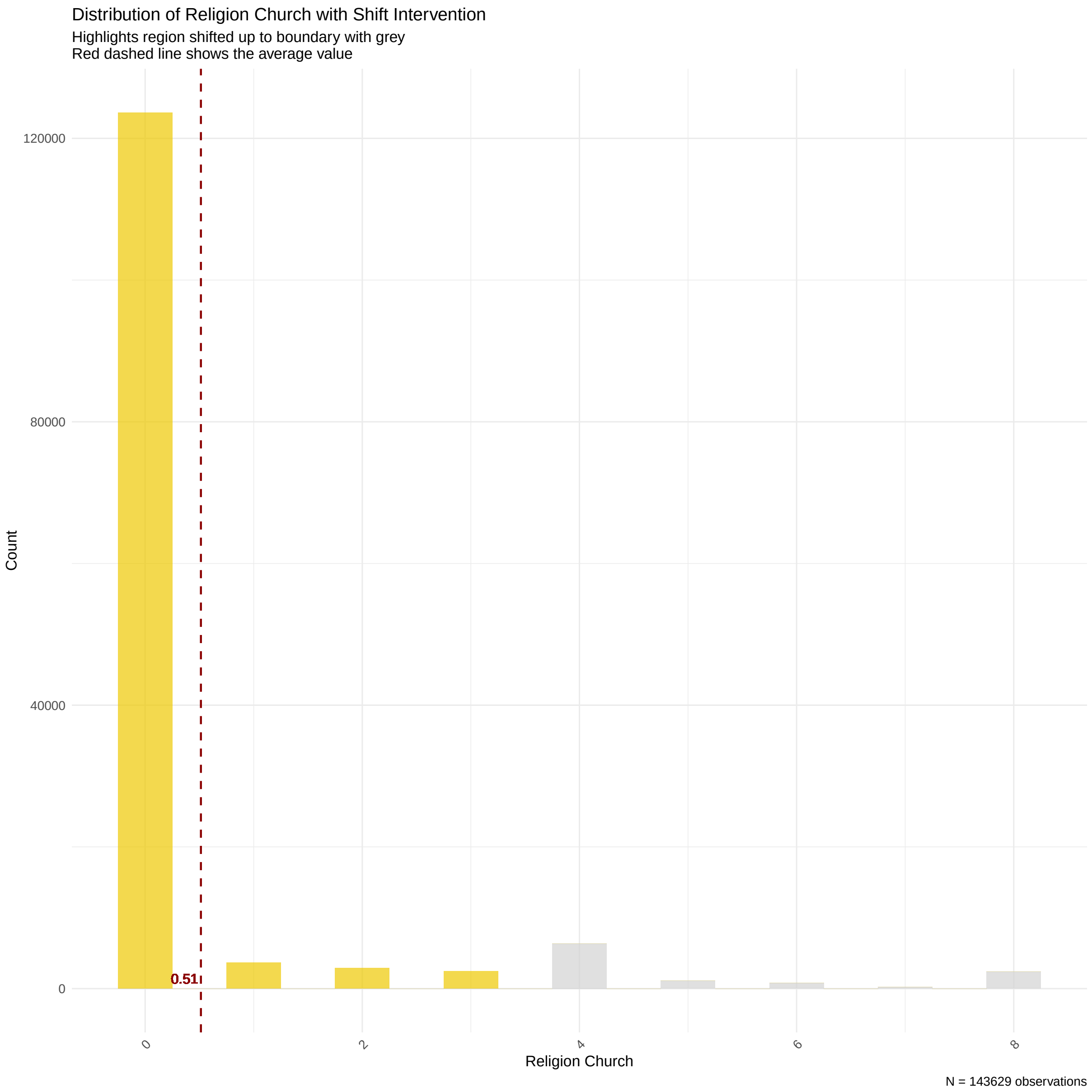

Figure 2: Histogram of Religious Service Attendance (first exposure wave): bars coloured gold are shifted under the shift to weekly exposure. Those without colour are estimated under the identity policy.

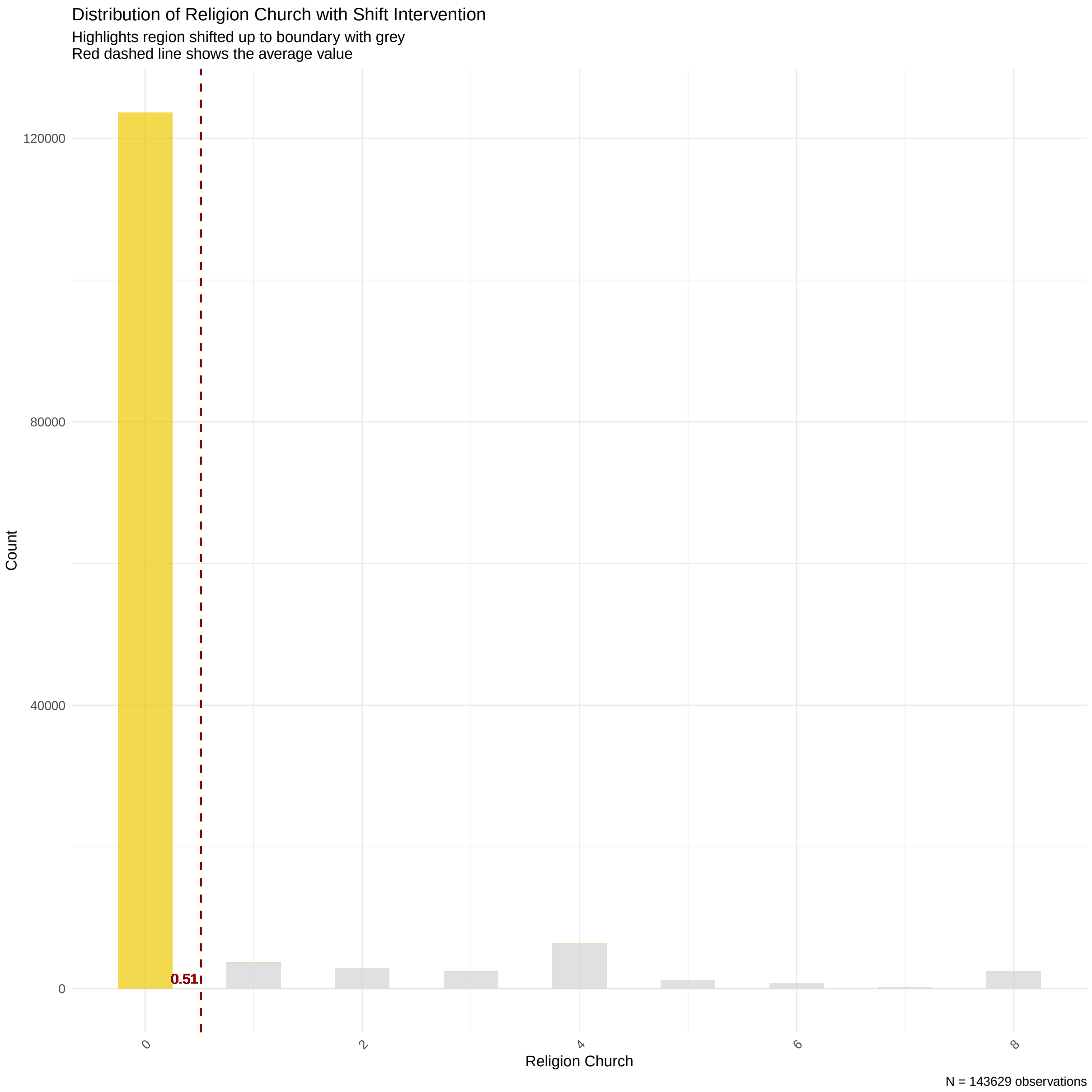

Figure 3: Histogram of Religious Service Attendance (first exposure wave): bars coloured gold are shifted under the shift to monthly exposure. Those without colour are estimated under the identity policy.

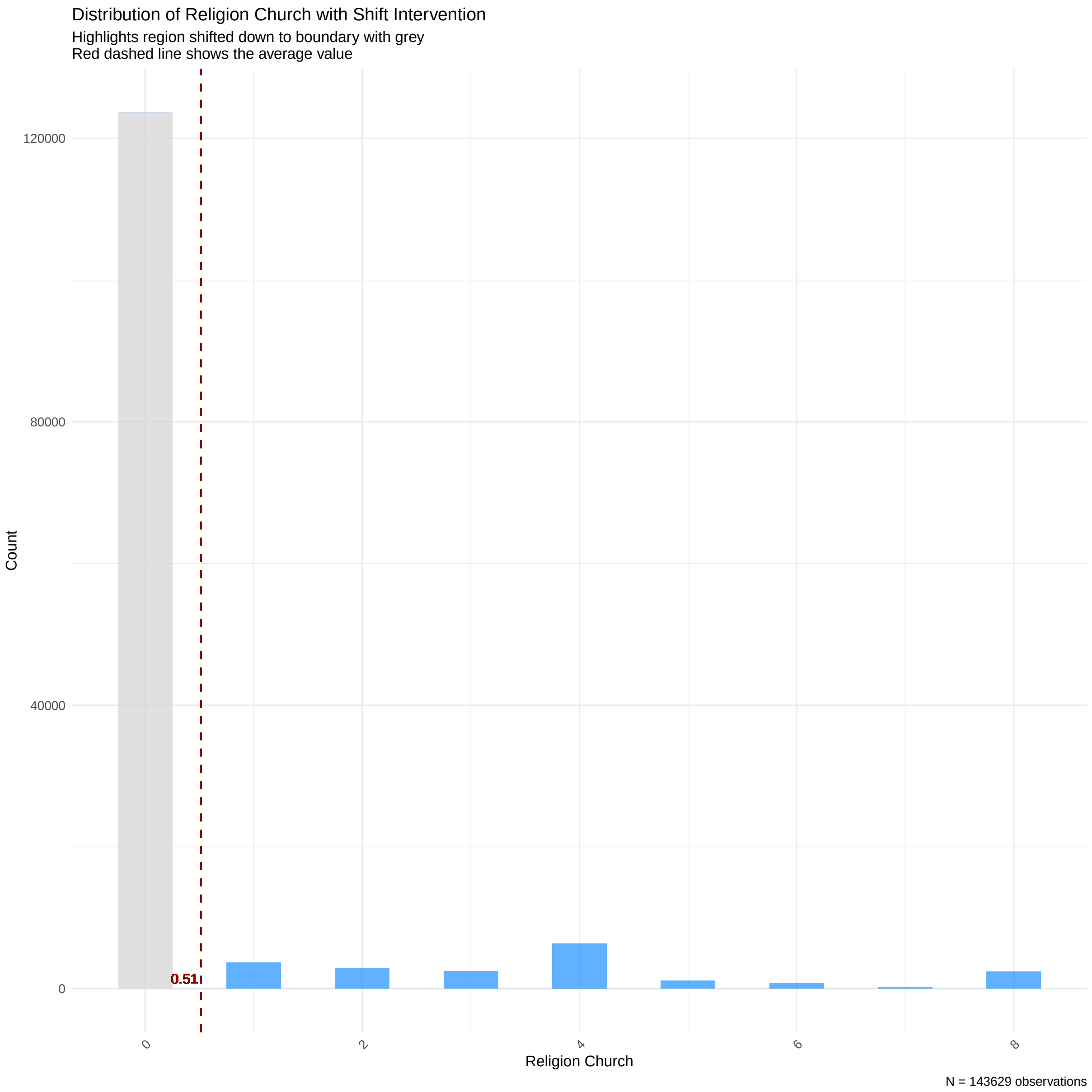

Figure 4: Histogram of Religious Service Attendance (first exposure wave): bars coloured blue are shifted under the shift to zero exposure. Those without colour are estimated under the identity policy.

These transition matrices capture shifts in states between consecutive waves. Each cell shows the count of individuals transitioning from one state to another. Rows are the initial state (From), columns the subsequent state (To). **Diagonal entries** (in **bold**) mark those who stayed in the same state.

Table 9: Transition Matrix From Wave 2018 to Wave 2019 among baseline wave non-attenders

From / To	State 0	State 1	Total
State 0	27380	845	28225

Table 10: Transition Matrix From Wave 2021 to Wave 2022 (among baseline wave non-attenders)

From / To	State 0	State 1	Total
State 0	16631	355	16986
State 1	330	170	500

Table 9 and Table 10 present transitions for the non-attender sample at baseline. Again, religious service attendance was not asked in wave 2020-2021 and was partially missing in wave 2021-2022: our six-wave study encompasses two interventions on attendance.

Supplement S5: Positivity Diagnostics

Overview

Positivity is the assumption that the data contain enough observed variation to estimate what would happen under each hypothetical policy. Positivity asks whether, for the policies we compare, the data provide sufficient empirical support across covariate strata at each time point to identify those policies. In longitudinal modified-treatment-policy analyses, the Sequential Doubly Robust (SDR) estimator recovers causal effects by reweighting the observed data to mimic each policy. The weights are products of per-wave density ratios:

$$w_i = \prod_{t=1}^{T} r_{i,t}(A_{i,t}, H_{i,t}), \quad \text{where} \quad r_{i,t} = \frac{g_t(d_t(A_{i,t}) \mid H_{i,t})}{g_t(A_{i,t} \mid H_{i,t})}.$$

Here, $g_t(\cdot \mid H_{i,t})$ is the conditional density of exposure at wave t given history $H_{i,t}$, and $d_t(\cdot)$ is the policy shift function. Ratios $r_{i,t} > 1$ up-weight observations more typical of the policy; ratios $r_{i,t} < 1$ down-weight others. Over multiple waves, these ratios compound: individuals whose observed trajectories are incompatible with the policy accumulate very small products and contribute minimal information.

We assess positivity using complementary diagnostics that quantify both the prevalence of problematic trajectories and the remaining information after reweighting. Our approach evaluates empirical support without imposing rigid cutoffs, recognising that positivity is a matter of degree rather than a binary pass/fail (Kennedy 2019).

Diagnostic Framework

Censoring versus treatment positivity. In longitudinal modified treatment policy analyses, exact zeros in density ratios ($r_{i,t} = 0$) almost always reflect censoring (dropout) rather than treatment-positivity violations. We therefore report censoring rates separately and compute all positivity diagnostics on uncensored rows only ($r_{i,t} > 0$). Censoring is addressed using inverse probability of censoring weights (IPCW), which upweight individuals who remain under observation to represent those who dropped out.

Product-of-ratios diagnostic. The Sequential Doubly Robust estimator reweights by the product of density ratios along each individual's observed trajectory. We compute this product for each uncensored individual and report the proportion with $\prod_t r_{i,t} < 10^{-1}$. This threshold corresponds to cumulative down-weighting to 10% of baseline; over four intervention waves, this implies an average per-wave ratio of approximately $(0.1)^{1/4} \approx 0.56$. We adopt a screening rule that flags policies where more than 10% of uncensored individuals fall below this threshold, indicating substantial information loss from a non-negligible fraction of the sample. This diagnostic directly captures the cumulative positivity constraint that the estimator faces: if many individuals' observed trajectories are incompatible with the policy, the effective sample size collapses.

Effective sample size (ESS). We summarise how much information remains after reweighting using the effective sample size:

$$\text{ESS} = \frac{(\sum_i w_i)^2}{\sum_i w_i^2},$$

computed on uncensored rows and reported per wave alongside two normalisations: ESS_+/N_+ (relative to uncensored person-rows) and ESS_+/N_{pt} (relative to total person-time rows, including censored). As a rule of thumb, $\text{ESS}_+/N_+ < 0.50$ flags strained positivity, indicating that reweighting has discarded more than half the effective information. The ESS complements the product-of-ratios test: the latter identifies *how many* individuals have problematic trajectories, while ESS quantifies *how much information remains* overall.

Per-wave density-ratio distributions. For each wave and policy, we report the range, mean $\pm$ SD, coefficient of variation, tail mass (proportion above thresholds such as $r > 10$, $r > 25$), and extreme quantiles (0.1%, 1%, 5%, median, 95%, 99.9%). Large tail mass or extreme weights indicate that the estimator is relying heavily on a few observations. However, in our data, positivity violations manifest primarily as severe down-weighting (small ratios) rather than explosive up-weighting (large ratios).

Policy rates (where applicable). For binary exposures, we report the policy-implied $\Pr(A_t = 1)$ by reweighting the observed data at each wave: $\hat{p}_t = \sum_i r_{i,t} A_{i,t} / \sum_i r_{i,t}$. This helps interpret what the policy does in practice.

These diagnostics are computed using `margot` utilities (e.g., `margot_interpret_lmtp_positivity()`), which automate ESS and tail metrics on uncensored rows and report censoring separately.

Three-wave positivity diagnostics

We examined positivity for the three-wave (single-shot) deterministic interventions, which impose a shift at t_1 and measure outcomes at t_2. Both the weekly religious attendance intervention and the zero (secular socialising) intervention fail our positivity criteria.

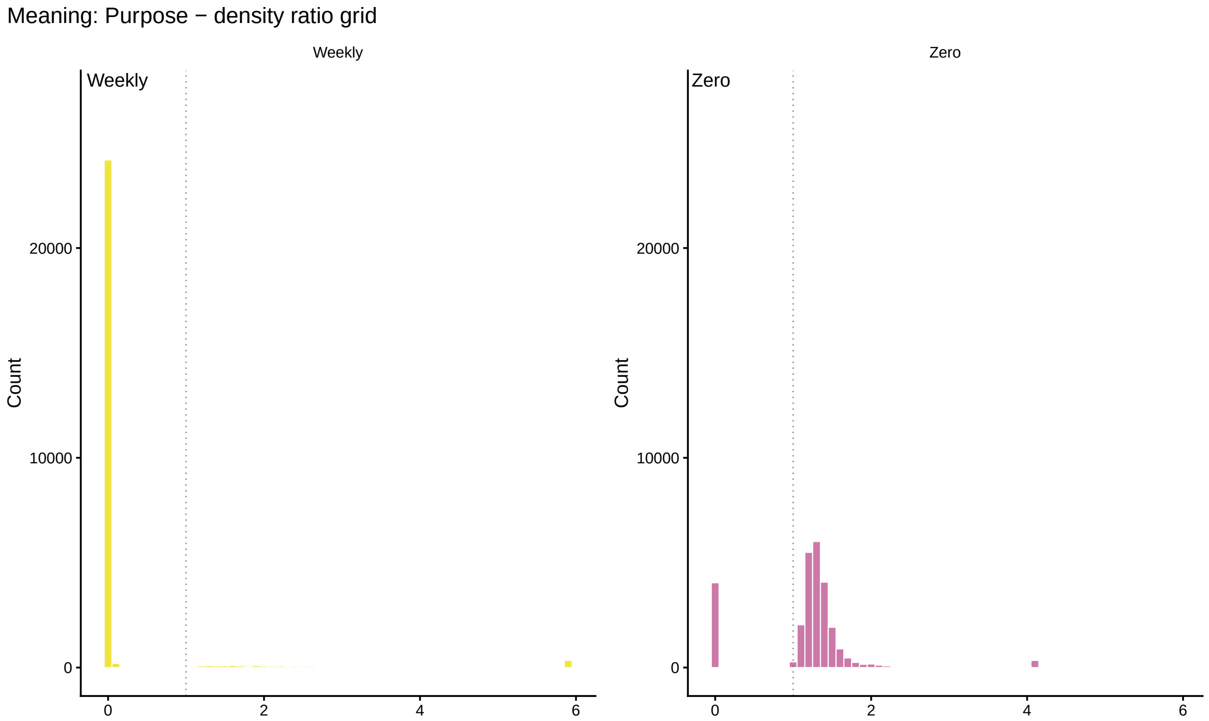

Figure 5: Three-wave positivity diagnostics for deterministic religious attendance and socialising interventions. Both interventions fail the positivity assumption.

Table 11 summarises the positivity diagnostics for the three-wave deterministic interventions.

Table 11: Three-wave positivity diagnostics summary. Both the weekly religious attendance shift and the zero (socialising) shift fail the product-of-ratios screen.

Shift	Support	Zero %	Outside [0.1000, 10.0000]	Prod < 0.1000	Prod > 10.0000	Cum ESS
Religious	Limited	20.839	91.414	91.414	0	1867.917
Secular	Limited	20.839	15.265	15.265	0	20709.562

Three-wave religious attendance

The weekly religious attendance intervention clearly fails the positivity assumption. Among uncensored rows, 72.4% of trajectories have products of density ratios below 10^{-1}, far exceeding the 10% threshold. The effective sample size relative to person-time is only 5.5%, indicating that reweighting discards nearly all effective information.

Wave-specific diagnostics show density ratios ranging from 0.003 to 5.862 at the baseline attendance wave, with a median of only 0.010, indicating that the vast majority of observations are severely down-weighted. These diagnostics indicate that deterministic weekly attendance interventions are not supported by the three-wave data.

Three-wave socialising

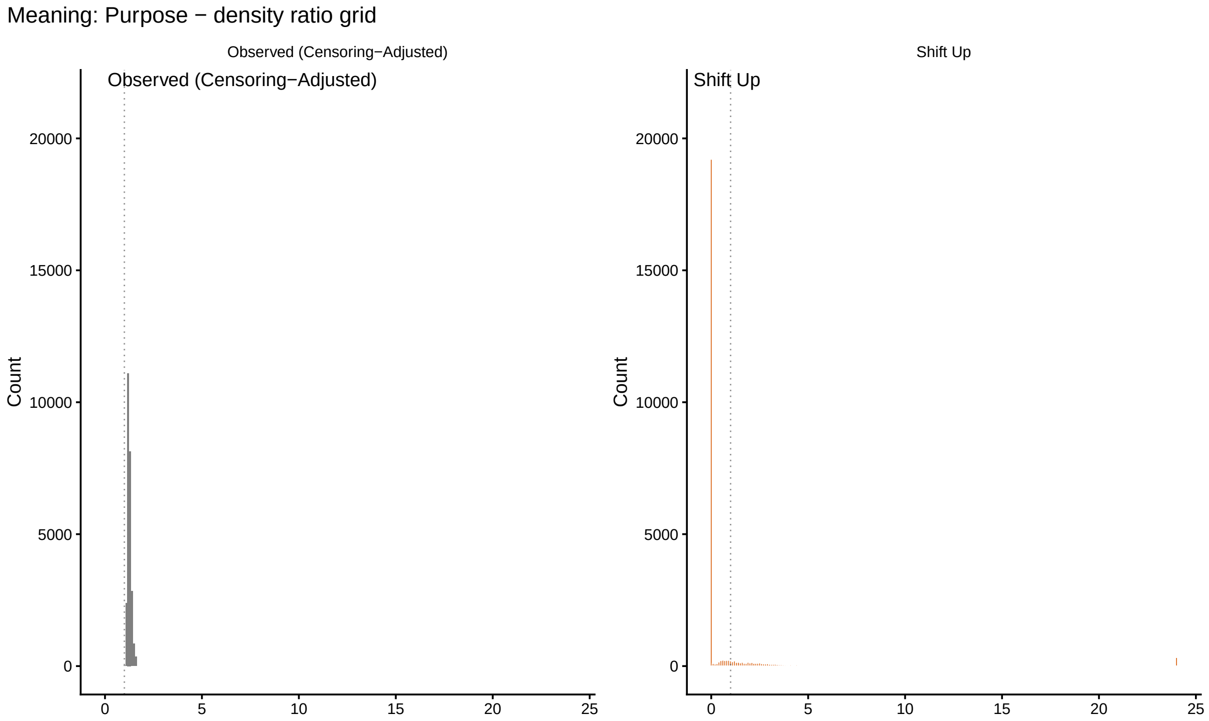

Figure 6: Three-wave positivity diagnostics for the deterministic socialising shift-up intervention (+1 hour/week). The shift-up intervention fails the positivity assumption.

Table 12 summarises the positivity diagnostics for the three-wave socialising intervention.

Table 12: Three-wave positivity diagnostics for socialising interventions. The shift-up intervention fails the product-of-ratios screen; the identity (null) policy passes.

Shift	Support	Zero %	Outside [0.1000, 10.0000]	Prod < 0.1000	Prod > 10.0000	Cum ESS
Observed (Censoring-Adjusted)	Adequate	20.436	0.0	0.000	0.000	25550.041
Shift Up	Limited	20.436	78.3	74.907	3.394	2248.684

The shift-up intervention (+1 hour/week socialising) fails the positivity assumption severely. Among uncensored rows, 59.6% of trajectories have products of density ratios below 10^{-1}, far exceeding the 10% threshold. The effective sample size relative to person-time is only 7.0%, indicating that reweighting discards nearly all effective information. Wave-specific diagnostics show density ratios ranging from 0.001 to 24.021 at the baseline wave, with a median of only 0.002, indicating severe down-weighting for most observations. By contrast, the identity (null) policy passes with 0% of trajectories below the threshold and an effective sample size of 79.1%. These diagnostics indicate that deterministic socialising interventions are not supported by the three-wave data, reinforcing the need to interpret the null results for the socialising control reported in Supplement S6 Part B cautiously.

Detailed wave-by-wave diagnostics: full baseline cohort

As documented in the main text, deterministic interventions that increase attendance (to weekly or monthly levels) exhibit severe positivity violations. Below, we report detailed wave-by-wave diagnostics generated by the `margot_positivity_report()` function in the `margot` package (Bulbulia 2024a).

Interpretation guide. The density ratio r_t reflects how much the intervention shifts the exposure distribution relative to observed behaviour. Values near 1.0 indicate the intervention is well-supported; values near 0 indicate the intervention requires extrapolation beyond observed data. We report the mean, standard deviation, coefficient of variation (CV), and selected quantiles. High CV values (>1.0) signal instability; quantiles near zero (e.g., p5% < 0.01) indicate that substantial portions of the sample lack empirical support for the intervention.

Identity (no intervention)

The identity policy serves as a reference benchmark and shows stable density ratios across all waves.

Wave	Censoring	Mean (SD)	CV	Range	p5%-p95%
Baseline (2018/19)	27.3%	1.38 (0.29)	0.21	0.85-2.93	1.08-1.87
Year 1 intervention	55.8%	1.66 (0.28)	0.17	1.03-2.93	1.30-2.19
Year 4 intervention	75.8%	1.83 (0.50)	0.28	0.76-2.93	1.29-2.93

Note. No extreme weights observed. **Positivity is satisfied.**

Zero (shift to no attendance)

The zero policy also shows stable density ratios, indicating adequate support for estimating population-wide loss of attendance.

Wave	Censoring	Mean (SD)	CV	Range	p5%-p95%
Baseline (2018/19)	27.3%	1.38 (0.31)	0.22	0.83-3.46	1.08-1.87
Year 1 intervention	55.8%	1.54 (0.78)	0.51	0.001-3.46	0.004-2.67
Year 4 intervention	75.8%	1.71 (0.90)	0.53	0.007-3.46	0.01-3.46

Note. Although some density ratios approach zero at intervention waves, the overall distribution remains stable. **Positivity is marginally satisfied.**

Weekly (shift to weekly attendance)

The weekly intervention shows severe violations of positivity, with density ratios collapsing toward zero at intervention waves.

Wave	Censoring	Mean (SD)	CV	Range	p5%-p95%
Baseline (2018/19)	27.3%	1.38 (0.32)	0.23	0.80-3.87	1.08-1.86
Year 1 intervention	55.8%	0.27 (0.88)	**3.29**	0.004-3.87	0.008-2.87

Wave	Censoring	Mean (SD)	CV	Range	p5%-p95%
Year 4 intervention	75.8%	0.24 (0.88)	**3.68**	0.002-3.87	0.005-3.42

Note. The median density ratio at intervention waves is approximately 0.01, indicating that the vast majority of the sample lacks empirical support for weekly attendance. **Positivity fails.**

Monthly (shift to monthly attendance)

The monthly intervention shows similar positivity violations, though less extreme than those observed in the weekly intervention.

Wave	Censoring	Mean (SD)	CV	Range	p5%-p95%
Baseline (2018/19)	27.3%	1.38 (0.33)	0.24	0.80-4.26	1.08-1.86
Year 1 intervention	55.8%	0.41 (1.02)	**2.51**	0.003-4.26	0.009-2.98
Year 4 intervention	75.8%	0.42 (1.13)	**2.70**	0.005-4.26	0.008-4.26

Note. The median density ratio at intervention waves is approximately 0.01-0.02. **Positivity fails.**

Detailed wave-by-wave diagnostics: baseline non-attenders (deterministic)

We next report diagnostics for deterministic interventions restricted to the baseline population of non-attenders ($N = 38{,}477$). Here, we use binary coding of attendance as "at least monthly" versus "zero." Deterministic upward shifts fail to pass the positivity test even in this restricted sample.

Table 13: Summary of positivity diagnostics: deterministic shifts among baseline non-attenders

Shift	Support	Zero %	Outside [0.1000, 10.0000]	Prod < 0.1000	Prod > 10.0000	Cum ESS
Observed (Censoring-Adjusted)	Adequate	75.344	0.551	0.000	0.551	7543.608
Shift Zero	Adequate	75.344	2.968	2.221	0.746	7103.605
Shift Monthly	Limited	75.344	59.517	59.485	0.032	94.786

Table 14: Wave-by-wave positivity diagnostics: deterministic shifts among baseline non-attenders

	ESS/N_pt (precision): Baseline Attendance (2018/19)	ESS/N_pt (precision): Year 1 Intervention	ESS/N_pt (precision): Year 4 Intervention)
Observed (Censoring-Adjusted)	0.704	0.432	0.231
Zero	0.703	0.418	0.224
Monthly	0.707	0.014	0.007

Identity (no intervention)

Wave	Censoring	Mean (SD)	CV	Range	p5%-p95%
Baseline (2018/19)	26.9%	1.37 (0.27)	0.20	0.69-2.85	1.09-1.83
Year 1 intervention	55.6%	1.66 (0.28)	0.17	1.04-2.85	1.30-2.18
Year 4 intervention	75.3%	1.81 (0.47)	0.26	0.97-2.85	1.30-2.85

Note. **Positivity is satisfied.**

Zero (shift to no attendance)

Wave	Censoring	Mean (SD)	CV	Range	p5%-p95%
Baseline (2018/19)	26.9%	1.37 (0.28)	0.20	0.85-2.94	1.09-1.84
Year 1 intervention	55.6%	1.66 (0.42)	0.25	0.006-2.94	1.25-2.26
Year 4 intervention	75.3%	1.80 (0.58)	0.32	0.025-2.94	1.24-2.94

Note. **Positivity is marginally satisfied.**

Monthly (shift to monthly attendance)

Wave	Censoring	Mean (SD)	CV	Range	p5%-p95%
Baseline (2018/19)	26.9%	1.36 (0.25)	0.19	0.83-2.48	1.09-1.83
Year 1 intervention	55.6%	0.08 (0.43)	**5.60**	0.000-2.48	0.000-0.002
Year 4 intervention	75.3%	0.07 (0.41)	**5.84**	0.000-2.48	0.000-0.002

Note. The median density ratio at intervention waves is approximately 0.001, with 95% of observations below 0.002. **Positivity fails severely.**

Detailed wave-by-wave diagnostics: baseline non-attenders (incremental propensity score)

Finally, we report positivity diagnostics for the incremental propensity score interventions, which are the primary analysis in this study.

Identity (reference)

Wave	Censoring	Mean (SD)	CV	Range	p5%-p95%
Baseline (2018/19)	26.9%	1.37 (0.27)	0.20	0.82-2.87	1.09-1.83
Year 1 intervention	55.6%	1.66 (0.28)	0.17	1.00-2.87	1.30-2.18
Year 4 intervention	75.3%	1.81 (0.48)	0.26	1.00-2.87	1.30-2.87

Note. **Positivity is satisfied.**

Zero (reference)

Wave	Censoring	Mean (SD)	CV	Range	p5%-p95%
Baseline (2018/19)	26.9%	1.37 (0.28)	0.20	0.85-2.94	1.09-1.84
Year 1 intervention	55.6%	1.66 (0.42)	0.25	0.006-2.94	1.25-2.26
Year 4 intervention	75.3%	1.80 (0.58)	0.32	0.025-2.94	1.24-2.94

Note. **Positivity is satisfied.**

$\delta = 2$ increase in initiation probability

Wave	Censoring	Mean (SD)	CV	Range	p5%-p95%
Baseline (2018/19)	26.9%	1.37 (0.26)	0.19	0.83-2.53	1.09-1.82
Year 1 intervention	55.6%	0.87 (0.32)	0.37	0.48-2.53	0.66-1.13
Year 4 intervention	75.3%	0.93 (0.40)	0.43	0.36-2.53	0.61-1.79

Note. Density ratios remain well above zero across all waves. **Positivity is satisfied.**

$\delta = 5$ increase in initiation probability

Wave	Censoring	Mean (SD)	CV	Range	p5%-p95%
Baseline (2018/19)	26.9%	1.37 (0.25)	0.19	0.86-2.51	1.09-1.82
Year 1 intervention	55.6%	0.39 (0.38)	0.97	0.17-2.51	0.25-0.48

Wave	Censoring	Mean (SD)	CV	Range	p5%-p95%
Year 4 intervention	75.3%	0.42 (0.37)	0.90	0.10-2.51	0.25-0.71

Note. Although density ratios are lower than for $\delta = 2$, the 5th percentile remains above 0.10 at all waves. **Positivity is satisfied.**

$\delta = 10$ increase in initiation probability

Wave	Censoring	Mean (SD)	CV	Range	p5%-p95%
Baseline (2018/19)	26.9%	1.37 (0.25)	0.19	0.80-2.49	1.09-1.83
Year 1 intervention	55.6%	0.24 (0.40)	**1.69**	0.07-2.49	0.12-0.25
Year 4 intervention	75.3%	0.25 (0.39)	**1.54**	0.04-2.49	0.13-0.37

Note. Density ratios approach the lower threshold at intervention waves. The 5th percentile falls to 0.12-0.13, and CV exceeds 1.5. **Positivity is strained; results should be interpreted with caution.**

Supplement S6: Results

Part A: Cross-sectional Associations (Descriptive)

Table 15 presents the results of the 'naive' cross-sectional model.

Table 15: Model for cross-sectional associations (the naive regression). Results for outcome regression in top half of table. Results for treatment regression on the bottom.

	ATE	0.1 %	99.9 %	E-Value	E-Value Bound
Alcohol Frequency Weekly	-0.406	-0.445	-0.367	2.251	2.140
Meaning: Purpose	0.399	0.361	0.437	2.231	2.123
Alcohol Intensity	-0.348	-0.385	-0.311	2.088	1.986
Meaning: Sense	0.313	0.275	0.351	1.991	1.888
Gratitude	0.247	0.209	0.285	1.814	1.712
Forgiveness	0.193	0.154	0.232	1.670	1.566
Sexual Satisfaction	0.185	0.146	0.224	1.649	1.544
Social Support	0.153	0.115	0.191	1.564	1.459
Neighbourhood Community	0.153	0.114	0.192	1.564	1.457
Life Satisfaction	0.115	0.076	0.154	1.460	1.348
Social Belonging	0.096	0.057	0.135	1.407	1.289
Body Satisfaction	0.095	0.057	0.133	1.404	1.289
Self Esteem	0.090	0.051	0.129	1.390	1.270
Hours of Exercise (log)	-0.086	-0.124	-0.048	1.378	1.259
Personal Well-being Index	0.078	0.039	0.117	1.355	1.229
Anxiety	-0.065	-0.104	-0.026	1.315	1.180
Depression	-0.056	-0.094	-0.018	1.287	1.144
Short Form Health	0.055	0.017	0.093	1.284	1.140
Rumination	-0.046	-0.084	-0.008	1.254	1.091
Sleep	0.040	0.002	0.078	1.233	1.040
BMI	0.038	0.000	0.076	1.226	1.000
Self Control	0.036	-0.003	0.075	1.219	1.000
Fatigue	-0.033	-0.072	0.006	1.208	1.000
Perfectionism	0.015	-0.024	0.054	1.132	1.000

Part B: Deterministic Intervention Results

As documented in the main text and Supplement S5, deterministic interventions that set religious attendance to weekly or monthly levels fail positivity diagnostics. We present these results for transparency but do not emphasise them in the primary inference. The pattern of results is broadly consistent with the probabilistic interventions reported in Part C, showing gains concentrated in meaning, purpose, forgiveness, and sexual satisfaction, but estimates should be interpreted cautiously given the severe positivity violations.

Three-wave deterministic interventions

Table 16 presents the results of the three-wave 'single-shot' intervention contrasting weekly attendance with none. Note that this deterministic intervention fails positivity diagnostics (see Supplement S5).

Table 16: Three-wave deterministic shift (longitudinal modified treatment policy): weekly vs zero attendance; full baseline cohort. Positivity diagnostics indicate this causal estimand is not well supported by the data.

	ATE	0.1 %	99.9 %	E-Value	E-Value Bound
Meaning: Purpose	0.119	0.091	0.147	1.471	1.392
Meaning: Sense	0.099	0.070	0.128	1.415	1.331
Gratitude	0.080	0.051	0.109	1.361	1.271
Forgiveness	0.076	0.048	0.104	1.349	1.260
Alcohol Frequency	-0.073	-0.095	-0.051	1.340	1.271
Alcohol Intensity	-0.048	-0.079	-0.017	1.261	1.143
Social Support	0.041	0.013	0.069	1.237	1.121
Body Satisfaction	0.039	0.012	0.066	1.230	1.118
Social Belonging	0.038	0.012	0.064	1.226	1.117
Personal Well-being Index	0.026	-0.001	0.053	1.181	1.000
Short Form Health	-0.026	-0.055	0.003	1.181	1.000
Neighbourhood Community	0.025	-0.005	0.055	1.176	1.000
Sexual Satisfaction	0.024	-0.006	0.054	1.172	1.000
Self Esteem	0.014	-0.011	0.039	1.127	1.000
Perfectionism	-0.014	-0.041	0.013	1.127	1.000
Hours of Exercise (log)	0.013	-0.022	0.048	1.122	1.000
Depression	-0.011	-0.040	0.018	1.111	1.000
Anxiety	-0.009	-0.037	0.019	1.099	1.000
Life Satisfaction	0.004	-0.022	0.030	1.064	1.000
Rumination	-0.004	-0.034	0.026	1.064	1.000
Self Control	-0.003	-0.027	0.021	1.055	1.000
Fatigue	0.003	-0.026	0.032	1.055	1.000
BMI	0.003	-0.013	0.019	1.055	1.000
Sleep	0.001	-0.033	0.035	1.031	1.000

Table 17 presents the results of shifting socialising up by one hour per week, contrasted with the natural course (identity). We do not find reliable effects on well-being merely by increasing socialising with the community over the course of one year. Note that this does not imply that socialising would not improve well-being; only that socialising a little more than one already does for one year, adjusting for a wide array of baseline confounders, does not produce reliable population-wide benefits as measured by our survey instruments. As noted in Supplement S5, the three-wave socialising intervention also fails positivity diagnostics.

Table 17: Three-wave negative control: +1 hour/week socialising (identity otherwise); full baseline cohort.

	ATE	0.1 %	99.9 %	E-Value	E-Value Bound
Social Support	-0.017	-0.073	0.039	1.141	1
Social Belonging	-0.006	-0.055	0.043	1.080	1
Sleep	0.021	-0.037	0.079	1.160	1
Short Form Health	-0.020	-0.074	0.034	1.155	1
Sexual Satisfaction	0.003	-0.057	0.063	1.055	1
Self Esteem	-0.023	-0.073	0.027	1.168	1
Self Control	0.006	-0.045	0.057	1.080	1
Rumination	0.009	-0.053	0.071	1.099	1
Personal Well-being Index	-0.017	-0.068	0.034	1.141	1
Perfectionism	0.032	-0.025	0.089	1.204	1
Neighbourhood Community	0.058	-0.001	0.117	1.293	1
Meaning: Sense	-0.004	-0.064	0.056	1.064	1
Meaning: Purpose	0.026	-0.027	0.079	1.181	1
Life Satisfaction	-0.005	-0.061	0.051	1.072	1
Hours of Exercise (log)	0.054	-0.017	0.125	1.280	1
Gratitude	-0.029	-0.086	0.028	1.192	1
Forgiveness	-0.003	-0.059	0.053	1.055	1
Fatigue	0.001	-0.056	0.058	1.031	1
Depression	0.049	-0.015	0.113	1.264	1
Body Satisfaction	0.014	-0.043	0.071	1.127	1
BMI	-0.005	-0.030	0.020	1.072	1
Anxiety	0.018	-0.035	0.071	1.146	1
Alcohol Intensity	0.020	-0.040	0.080	1.155	1
Alcohol Frequency	0.022	-0.024	0.068	1.164	1

Similarly, as presented in Table 18, a population-wide reduction in socialising by 1 hour (down to zero) does not generate strong population-wide harm signals using our survey measures.

Table 18: Three-wave negative control: -1 hour/week socialising to minimum zero (identity otherwise); full baseline cohort.

	ATE	0.1 %	99.9 %	E-Value	E-Value Bound
Social Support	-0.003	-0.009	0.003	1.055	1
Social Belonging	-0.003	-0.008	0.002	1.055	1
Sleep	0.002	-0.006	0.010	1.045	1
Short Form Health	-0.002	-0.008	0.004	1.045	1
Sexual Satisfaction	-0.001	-0.008	0.006	1.031	1
Self Esteem	0.000	-0.006	0.006	1.000	1
Self Control	0.000	-0.005	0.005	1.000	1
Rumination	0.001	-0.006	0.008	1.031	1
Personal Well-being Index	-0.002	-0.007	0.003	1.045	1
Perfectionism	0.000	-0.006	0.006	1.000	1
Neighbourhood Community	-0.003	-0.009	0.003	1.055	1
Meaning: Sense	-0.001	-0.007	0.005	1.031	1
Meaning: Purpose	0.000	-0.005	0.005	1.000	1
Life Satisfaction	-0.002	-0.008	0.004	1.045	1
Hours of Exercise (log)	-0.007	-0.015	0.001	1.087	1
Gratitude	-0.001	-0.007	0.005	1.031	1
Forgiveness	-0.002	-0.008	0.004	1.045	1
Fatigue	-0.001	-0.007	0.005	1.031	1
Depression	0.001	-0.006	0.008	1.031	1
Body Satisfaction	-0.005	-0.011	0.001	1.072	1
BMI	0.000	-0.003	0.003	1.000	1
Anxiety	0.000	-0.006	0.006	1.000	1
Alcohol Intensity	0.000	-0.007	0.007	1.000	1
Alcohol Frequency	0.000	-0.005	0.005	1.000	1

Six-wave deterministic interventions

Table 19 presents the results of the six-wave multi-wave intervention, contrasting weekly attendance over this horizon with none.

Table 19: Six-wave deterministic shift: weekly vs zero attendance; full baseline cohort; outcomes ordered by robustness to unmeasured confounding.

	ATE	0.1 %	99.9 %	E-Value	E-Value Bound
Meaning: Purpose	0.106	0.043	0.169	1.435	1.245
Sexual Satisfaction	0.100	0.047	0.153	1.418	1.259
Meaning: Sense	0.069	0.011	0.127	1.327	1.111
Forgiveness	0.065	0.014	0.116	1.315	1.127
Personal Well-being Index	0.060	0.006	0.114	1.300	1.079
Neighbourhood Community	0.053	-0.008	0.114	1.277	1.000
Gratitude	0.051	-0.007	0.109	1.271	1.000
Alcohol Intensity	-0.048	-0.100	0.004	1.261	1.000
Body Satisfaction	0.045	-0.012	0.102	1.250	1.000
Alcohol Frequency Weekly	-0.037	-0.072	-0.002	1.222	1.041
Fatigue	-0.035	-0.096	0.026	1.215	1.000
Life Satisfaction	0.026	-0.026	0.078	1.181	1.000
Depression	-0.022	-0.085	0.041	1.164	1.000
Social Support	0.021	-0.029	0.071	1.160	1.000
Self Control	-0.018	-0.066	0.030	1.146	1.000
BMI	0.017	-0.022	0.056	1.141	1.000
Rumination	0.014	-0.044	0.072	1.127	1.000
Sleep	-0.012	-0.098	0.074	1.116	1.000
Self Esteem	0.011	-0.040	0.062	1.111	1.000
Social Belonging	0.011	-0.042	0.064	1.111	1.000
Short Form Health	-0.010	-0.061	0.041	1.105	1.000
Anxiety	-0.007	-0.063	0.049	1.087	1.000
Perfectionism	0.006	-0.042	0.054	1.080	1.000
Hours of Exercise (log)	-0.002	-0.066	0.062	1.045	1.000

Table 20 presents the results of the six-wave multi-wave intervention, contrasting weekly attendance over this horizon with the identity policy.

Table 20: Six-wave deterministic shift: weekly attendance versus identity (no change); full baseline cohort.

	ATE	0.1 %	99.9 %	E-Value	E-Value Bound
Meaning: Purpose	0.083	0.037	0.129	1.369	1.224
Sexual Satisfaction	0.064	0.021	0.107	1.312	1.159
Meaning: Sense	0.062	0.016	0.108	1.306	1.136
Forgiveness	0.049	0.007	0.091	1.264	1.084
Personal Well-being Index	0.045	0.005	0.085	1.250	1.072
Gratitude	0.037	-0.009	0.083	1.222	1.000
Neighbourhood Community	0.035	-0.012	0.082	1.215	1.000
Fatigue	-0.035	-0.081	0.011	1.215	1.000
Body Satisfaction	0.035	-0.007	0.077	1.215	1.000
Alcohol Intensity	-0.028	-0.073	0.017	1.189	1.000
Life Satisfaction	0.027	-0.016	0.070	1.185	1.000
Depression	-0.023	-0.072	0.026	1.168	1.000
Alcohol Frequency (weekly)	-0.021	-0.051	0.009	1.160	1.000
Social Support	0.019	-0.021	0.059	1.151	1.000
Social Belonging	0.013	-0.030	0.056	1.122	1.000
Self Esteem	0.012	-0.028	0.052	1.116	1.000
Self Control	-0.011	-0.048	0.026	1.111	1.000
BMI	0.009	-0.023	0.041	1.099	1.000
Anxiety	-0.007	-0.049	0.035	1.087	1.000
Short Form Health	-0.006	-0.048	0.036	1.080	1.000
Rumination	0.004	-0.043	0.051	1.064	1.000
Hours of Exercise (log)	-0.004	-0.054	0.046	1.064	1.000
Sleep	-0.003	-0.067	0.061	1.055	1.000
Perfectionism	0.002	-0.036	0.040	1.045	1.000

Table 21 presents the results of the six-wave multi-wave intervention, contrasting zero attendance over this horizon with the identity policy. Unlike shift-up interventions, this shift-down causal estimand satisfies the positivity condition. Population-wide loss of attendance yields one reliable effect: sexual satisfaction declines by -0.036 SD (E-value bound = 1.12). Point estimates for meaning-purpose, neighbourhood community, forgiveness, and gratitude trend negatively but do not meet the robustness threshold. Note that direct comparison with shift-up results (e.g., Table 20) is not warranted because shift-up interventions fail positivity diagnostics.

Table 21: Six-wave deterministic shift: population-wide loss of attendance versus identity (no change); full baseline cohort.

	ATE	0.1 %	99.9 %	E-Value	E-Value Bound
Sexual Satisfaction	-0.036	-0.060	-0.012	1.219	1.119
Meaning: Purpose	-0.023	-0.050	0.004	1.168	1.000
Alcohol Intensity	0.020	-0.007	0.047	1.155	1.000
Neighbourhood Community	-0.018	-0.046	0.010	1.146	1.000
Personal Well-being Index	-0.016	-0.040	0.008	1.137	1.000
Forgiveness	-0.016	-0.036	0.004	1.137	1.000
Alcohol Frequency (weekly)	0.016	-0.001	0.033	1.137	1.000
Gratitude	-0.014	-0.039	0.011	1.127	1.000
Rumination	-0.010	-0.035	0.015	1.105	1.000
Body Satisfaction	-0.010	-0.033	0.013	1.105	1.000
Sleep	0.009	-0.026	0.044	1.099	1.000
BMI	-0.008	-0.024	0.008	1.093	1.000
Self Control	0.007	-0.014	0.028	1.087	1.000
Meaning: Sense	-0.007	-0.033	0.019	1.087	1.000
Perfectionism	-0.004	-0.024	0.016	1.064	1.000
Short Form Health	0.003	-0.021	0.027	1.055	1.000
Social Support	-0.002	-0.024	0.020	1.045	1.000
Hours of Exercise (log)	-0.002	-0.033	0.029	1.045	1.000
Social Belonging	0.002	-0.022	0.026	1.045	1.000
Self Esteem	0.001	-0.021	0.023	1.031	1.000
Life Satisfaction	0.001	-0.021	0.023	1.031	1.000
Depression	-0.001	-0.029	0.027	1.031	1.000
Anxiety	0.000	-0.025	0.025	1.000	1.000
Fatigue	0.000	-0.027	0.027	1.000	1.000

Table 22 presents the results of the six-wave multi-wave intervention, contrasting monthly attendance over this horizon with none. This is the least behaviourally restrictive causal estimand. Expected gains from monthly attendance contrasted with zero attendance are evident for meaning, gratitude, and sexual satisfaction. Similar gains are evident in the next table, which contrasts monthly attendance and weekly attendance with the status quo.

Table 22: Six-wave deterministic shift: monthly-or-more vs zero attendance; full baseline cohort.

	ATE	0.1 %	99.9 %	E-Value	E-Value Bound
Sexual Satisfaction	0.083	0.024	0.142	1.369	1.173
Meaning: Purpose	0.047	-0.020	0.114	1.257	1.000
Body Satisfaction	0.040	-0.019	0.099	1.233	1.000
Gratitude	0.037	-0.024	0.098	1.222	1.000
Personal Well-being Index	0.036	-0.021	0.093	1.219	1.000
Neighbourhood Community	0.034	-0.032	0.100	1.211	1.000
Self Control	-0.032	-0.086	0.022	1.204	1.000
Sleep	-0.030	-0.127	0.067	1.196	1.000
Alcohol Intensity	-0.029	-0.083	0.025	1.192	1.000
Meaning: Sense	0.022	-0.042	0.086	1.164	1.000
BMI	0.019	-0.023	0.061	1.151	1.000
Forgiveness	0.018	-0.038	0.074	1.146	1.000
Alcohol Frequency Weekly	-0.013	-0.052	0.026	1.122	1.000
Social Support	0.011	-0.042	0.064	1.111	1.000
Short Form Health	0.011	-0.045	0.067	1.111	1.000
Rumination	0.011	-0.057	0.079	1.111	1.000
Hours of Exercise (log)	-0.009	-0.084	0.066	1.099	1.000
Anxiety	-0.008	-0.072	0.056	1.093	1.000
Fatigue	0.008	-0.062	0.078	1.093	1.000
Social Belonging	-0.006	-0.061	0.049	1.080	1.000
Self Esteem	-0.005	-0.063	0.053	1.072	1.000
Perfectionism	-0.005	-0.057	0.047	1.072	1.000
Depression	-0.005	-0.073	0.063	1.072	1.000
Life Satisfaction	0.000	-0.063	0.063	1.000	1.000

Table 23: Six-wave deterministic shifts: weekly and monthly attendance versus identity (no change); full baseline cohort.

	ATE	0.1 %	99.9 %	E-Value	E-Value Bound
Sexual Satisfaction	0.047	0.002	0.092	1.257	1.048
Body Satisfaction	0.030	-0.013	0.073	1.196	1.000
Self Control	-0.025	-0.064	0.014	1.176	1.000
Meaning: Purpose	0.024	-0.023	0.071	1.172	1.000
Gratitude	0.023	-0.023	0.069	1.168	1.000
Sleep	-0.021	-0.091	0.049	1.160	1.000
Personal Well-being Index	0.020	-0.021	0.061	1.155	1.000
Neighbourhood Community	0.016	-0.033	0.065	1.137	1.000
Meaning: Sense	0.015	-0.032	0.062	1.132	1.000
Short Form Health	0.014	-0.029	0.057	1.127	1.000
BMI	0.012	-0.020	0.044	1.116	1.000
Hours of Exercise (log)	-0.011	-0.067	0.045	1.111	1.000
Social Support	0.009	-0.031	0.049	1.099	1.000
Perfectionism	-0.009	-0.048	0.030	1.099	1.000

	ATE	0.1 %	99.9 %	E-Value	E-Value Bound
Anxiety	-0.008	-0.055	0.039	1.093	1.000
Fatigue	0.008	-0.042	0.058	1.093	1.000
Alcohol Intensity	-0.008	-0.052	0.036	1.093	1.000
Depression	-0.005	-0.057	0.047	1.072	1.000
Self Esteem	-0.004	-0.048	0.040	1.064	1.000
Social Belonging	-0.004	-0.046	0.038	1.064	1.000
Alcohol Frequency Weekly	0.003	-0.027	0.033	1.055	1.000
Forgiveness	0.002	-0.041	0.045	1.045	1.000
Rumination	0.001	-0.052	0.054	1.031	1.000
Life Satisfaction	0.001	-0.048	0.050	1.031	1.000

Part C: Probabilistic Intervention Results (Primary Analysis)

The following tables present results from incremental propensity score interventions, which are the primary analysis in this study. Unlike deterministic interventions, these probabilistic shifts preserve positivity (see Supplement S5 for diagnostics).

Figure 7 and the three tables that follow report the $\delta = \{2, 5, 10\}$ incremental propensity score interventions for the **baseline non-attending cohort**, a different target population and set of causal estimands from the deterministic analyses. Increasing the probability of initiating attendance is associated with corresponding increases in social-functional outcomes. In the $\delta = 10$ analysis, after Bonferroni adjustment ($\alpha = 0.05$) and using our E-value lower-bound threshold of 1.10, six outcomes meet the reliability criterion: **meaning and purpose** (average treatment effect [ATE] = 0.101, 95% CI: 0.059–0.143; E-value bound = 1.29), **sexual satisfaction** (ATE = 0.089, 95% CI: 0.047–0.131; E-value bound = 1.26), **forgiveness** (ATE = 0.081, 95% CI: 0.039–0.123; E-value bound = 1.23), **meaning and sense** (ATE = 0.080, 95% CI: 0.032–0.128; E-value bound = 1.21), **short-form health** (ATE = 0.076, 95% CI: 0.034–0.118; E-value bound = 1.21), and **body satisfaction** (ATE = 0.072, 95% CI: 0.033–0.111; E-value bound = 1.21). This profile matches the $\delta = 5$ results with larger magnitudes, as expected. As documented in the main text, the $\delta = 10$ intervention strains positivity support, so these estimates should be interpreted cautiously.

Table 24: Six-wave incremental propensity intervention: $\delta = 2$ increase in monthly-or-more attendance probability vs null; baseline non-attenders.

	ATT	0.1 %	99.9 %	E-Value	E-Value bound
Sexual Satisfaction	0.054	0.023	0.085	1.280	1.167
Meaning: Purpose	0.050	0.018	0.082	1.267	1.145
Forgiveness	0.046	0.014	0.078	1.254	1.126
Short Form Health	0.040	0.008	0.072	1.233	1.092
Meaning: Sense	0.040	0.004	0.076	1.233	1.064
Body Satisfaction	0.031	0.002	0.060	1.200	1.045
Rumination	0.024	-0.009	0.057	1.172	1.000
BMI	-0.022	-0.045	0.001	1.164	1.000
Personal Well-being Index	0.020	-0.011	0.051	1.155	1.000
Gratitude	0.019	-0.014	0.052	1.151	1.000
Self Control	-0.017	-0.044	0.010	1.141	1.000
Hours of Exercise (log)	0.016	-0.022	0.054	1.137	1.000
Alcohol Frequency (weekly)	-0.016	-0.038	0.006	1.137	1.000
Social Support	0.015	-0.014	0.044	1.132	1.000
Neighbourhood Community	-0.014	-0.049	0.021	1.127	1.000
Fatigue	-0.013	-0.046	0.020	1.122	1.000
Sleep	-0.012	-0.066	0.042	1.116	1.000
Self Esteem	0.010	-0.020	0.040	1.105	1.000
Life Satisfaction	0.006	-0.025	0.037	1.080	1.000
Alcohol Intensity	-0.006	-0.041	0.029	1.080	1.000
Anxiety	-0.005	-0.037	0.027	1.072	1.000
Social Belonging	-0.005	-0.036	0.026	1.072	1.000
Perfectionism	-0.003	-0.029	0.023	1.055	1.000
Depression	-0.001	-0.036	0.034	1.031	1.000

Table 25: Six-wave incremental propensity intervention: $\delta = 5$ increase in monthly-or-more attendance probability vs null; baseline non-attenders.

	ATT	0.1 %	99.9 %	E-Value	E-Value bound
Sexual Satisfaction	0.086	0.046	0.126	1.378	1.254

	ATT	0.1 %	99.9 %	E-Value	E-Value bound
Meaning: Purpose	0.085	0.044	0.126	1.375	1.248
Forgiveness	0.075	0.034	0.116	1.346	1.212
Meaning: Sense	0.071	0.025	0.117	1.334	1.179
Short Form Health	0.061	0.022	0.100	1.303	1.163
Body Satisfaction	0.058	0.021	0.095	1.293	1.160
Gratitude	0.046	0.002	0.090	1.254	1.046
Rumination	0.031	-0.012	0.074	1.200	1.000
BMI	-0.031	-0.060	-0.002	1.200	1.045
Personal Well-being Index	0.028	-0.010	0.066	1.189	1.000
Social Support	0.025	-0.013	0.063	1.176	1.000
Self Control	-0.025	-0.060	0.010	1.176	1.000
Neighbourhood Community	-0.025	-0.070	0.020	1.176	1.000
Hours of Exercise (log)	0.024	-0.024	0.072	1.172	1.000
Alcohol Frequency (weekly)	-0.023	-0.051	0.005	1.168	1.000
Self Esteem	0.020	-0.018	0.058	1.155	1.000
Fatigue	-0.016	-0.058	0.026	1.137	1.000
Sleep	-0.014	-0.082	0.054	1.127	1.000
Life Satisfaction	0.013	-0.027	0.053	1.122	1.000
Alcohol Intensity	-0.013	-0.059	0.033	1.122	1.000
Anxiety	-0.008	-0.049	0.033	1.093	1.000
Depression	0.005	-0.041	0.051	1.072	1.000
Perfectionism	-0.004	-0.038	0.030	1.064	1.000
Social Belonging	0.000	-0.040	0.040	1.000	1.000

Table 26: Six-wave incremental propensity intervention: $\delta = 10$ increase in monthly-or-more attendance probability vs null; baseline non-attenders.

	ATT	0.1 %	99.9 %	E-Value	E-Value bound
Meaning: Purpose	0.101	0.059	0.143	1.421	1.295
Sexual Satisfaction	0.089	0.047	0.131	1.387	1.259
Forgiveness	0.081	0.039	0.123	1.363	1.228
Meaning: Sense	0.080	0.032	0.128	1.361	1.205
Short Form Health	0.076	0.034	0.118	1.349	1.210
Body Satisfaction	0.072	0.033	0.111	1.337	1.207
Gratitude	0.048	0.002	0.094	1.261	1.042
Social Support	0.038	-0.001	0.077	1.226	1.000
Personal Well-being Index	0.035	-0.005	0.075	1.215	1.000
BMI	-0.034	-0.065	-0.003	1.211	1.059
Rumination	0.032	-0.013	0.077	1.204	1.000
Alcohol Frequency (weekly)	-0.032	-0.062	-0.002	1.204	1.047
Self Control	-0.030	-0.066	0.006	1.196	1.000
Hours of Exercise (log)	0.023	-0.026	0.072	1.168	1.000
Self Esteem	0.022	-0.018	0.062	1.164	1.000
Fatigue	-0.019	-0.062	0.024	1.151	1.000
Life Satisfaction	0.014	-0.028	0.056	1.127	1.000
Neighbourhood Community	-0.013	-0.059	0.033	1.122	1.000
Sleep	-0.013	-0.083	0.057	1.122	1.000
Alcohol Intensity	-0.013	-0.062	0.036	1.122	1.000
Perfectionism	-0.011	-0.046	0.024	1.111	1.000
Depression	0.006	-0.041	0.053	1.080	1.000
Anxiety	-0.006	-0.048	0.036	1.080	1.000

	ATT	0.1 %	99.9 %	E-Value	E-Value bound
Social Belonging	-0.006	-0.048	0.036	1.080	1.000

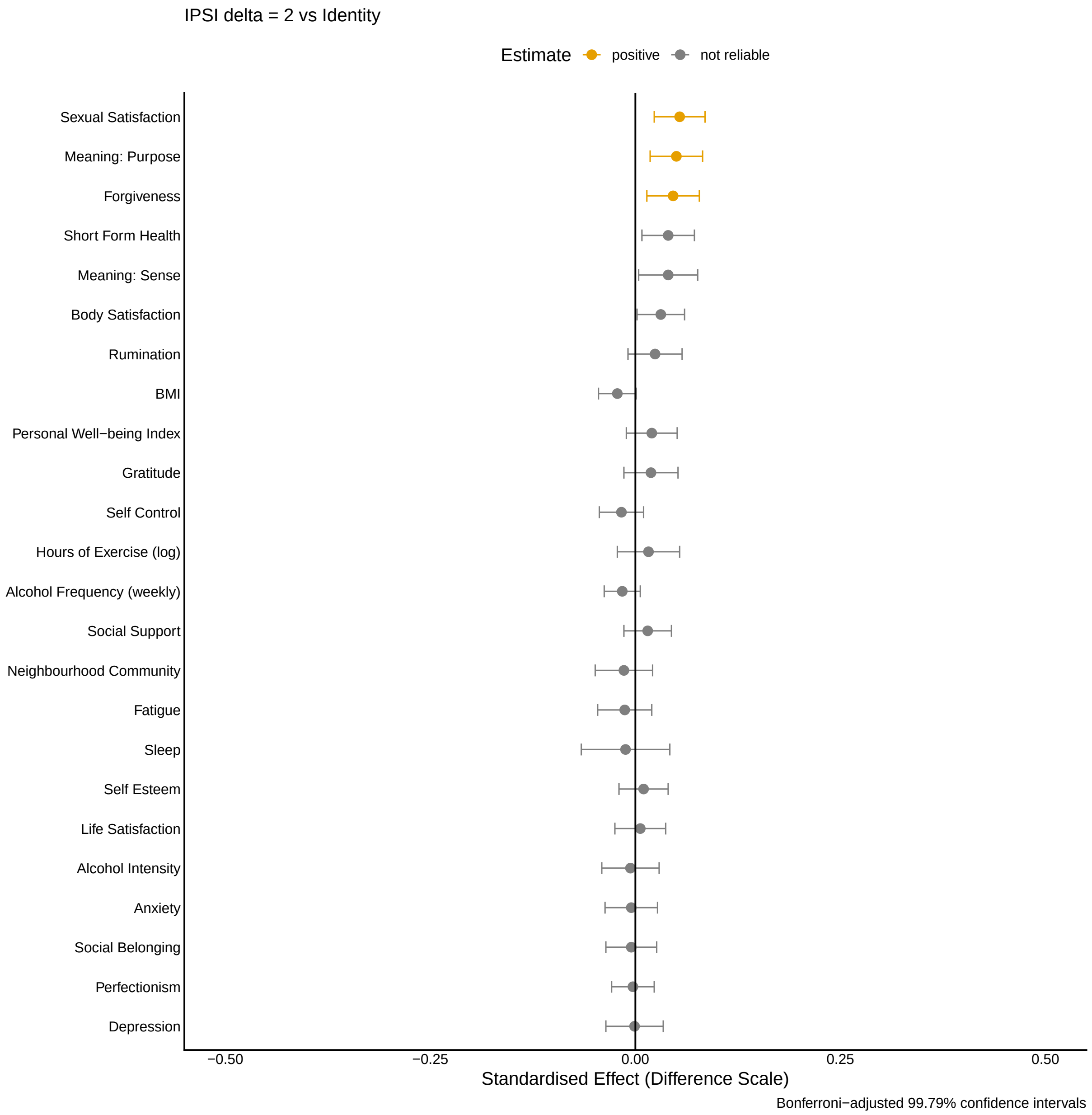

Figure 7: Causal effects of the two-fold incremental propensity score intervention ($\delta = 2$) among baseline non-attenders. Each row is one outcome. Points show estimated effects in standardised units; orange points have confidence intervals excluding zero and E-value lower bounds exceeding 1.10.

Supplement S7: Machine Learner Performance

Deterministic shifts: identity and zero

Figure 8 presents learner performance for the deterministic interventions, examining the identity (null) and zero shifts in religious attendance. These reference interventions pass positivity diagnostics and serve as benchmarks for the incremental propensity score interventions.

For the deterministic identity and zero interventions, the super learner ensemble showed a strong preference for regularised regression (glmnet) in both outcome and treatment models. For outcome regression, glmnet received 71-80% of the ensemble weight across waves, with ranger occasionally serving as runner-up (16-18%). Treatment regression showed a similar pattern, with glmnet receiving 59-67% of weight and xgboost contributing 30-36%. The consistency of learner selection across waves indicates stable model fitting for these reference interventions.

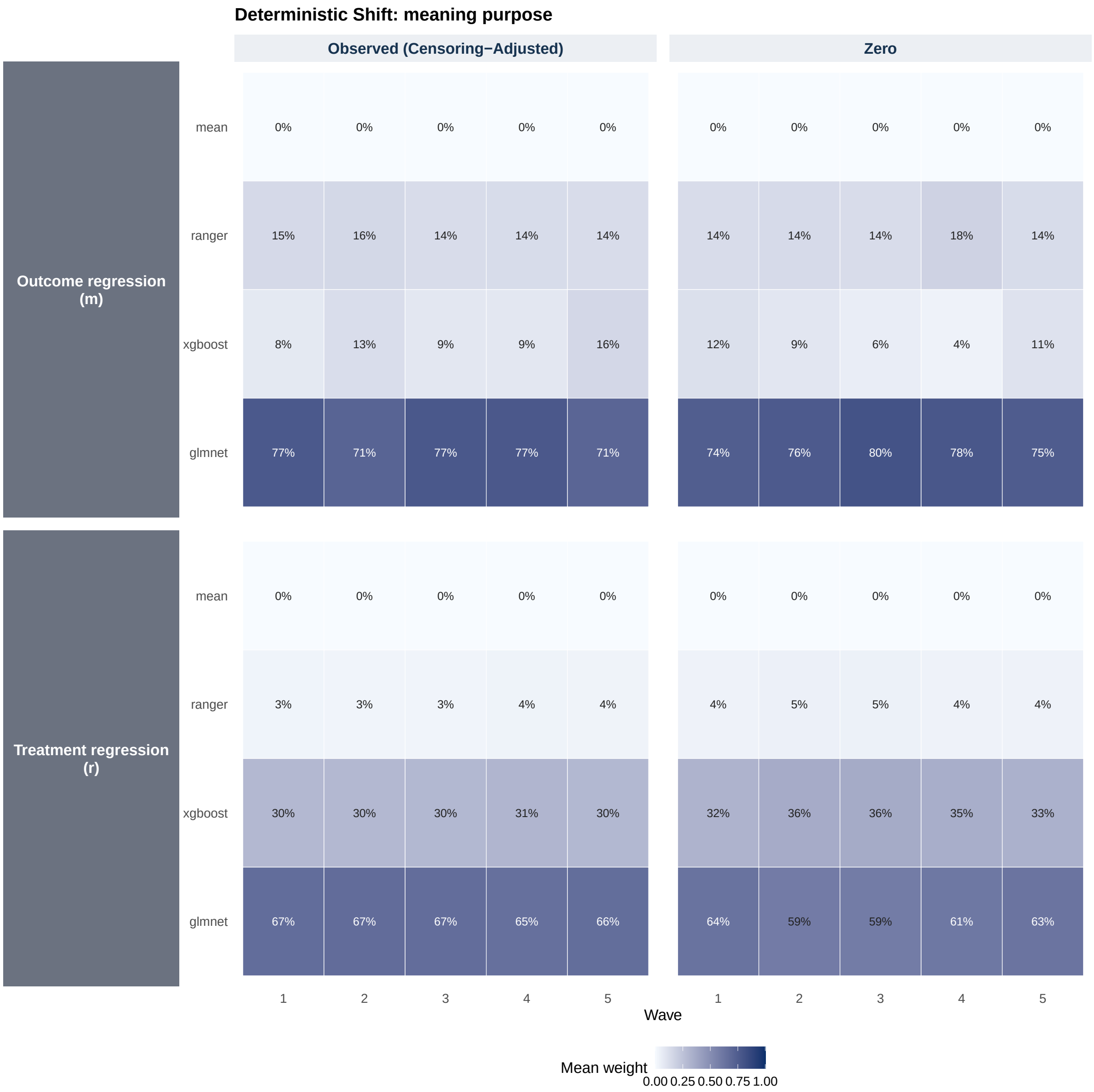

Figure 8: Learners: deterministic religious attendance interventions (identity and zero shifts). Results for outcome regression are in the top half of the figure. Results for treatment regression on the bottom.

Incremental propensity score interventions

Figure 9 presents learner performance for the incremental propensity-score interventions, examining the null (identity), zero, $\delta = 2$, and $\delta = 5$ probabilistic shifts in monthly attendance propensity among baseline non-attenders.

Across all incremental propensity score intervention shifts, the super learner ensemble showed a strong preference for regularised regression (glmnet) in both outcome and treatment models. For outcome regression, glmnet received 74-89% of the ensemble weight across waves and shifts, with xgboost as a distant runner-up (16-19%). This strong preference for glmnet indicates that the outcome-confounder relationships are well-captured by linear regularised models. Treatment regression showed a similar pattern, with glmnet accounting for 66-73% of the weight and xgboost for 20-28%. The consistency of learner selection across waves and intervention types provides evidence that the super learner ensemble converged to stable model weights, supporting the reliability of the doubly robust estimates.

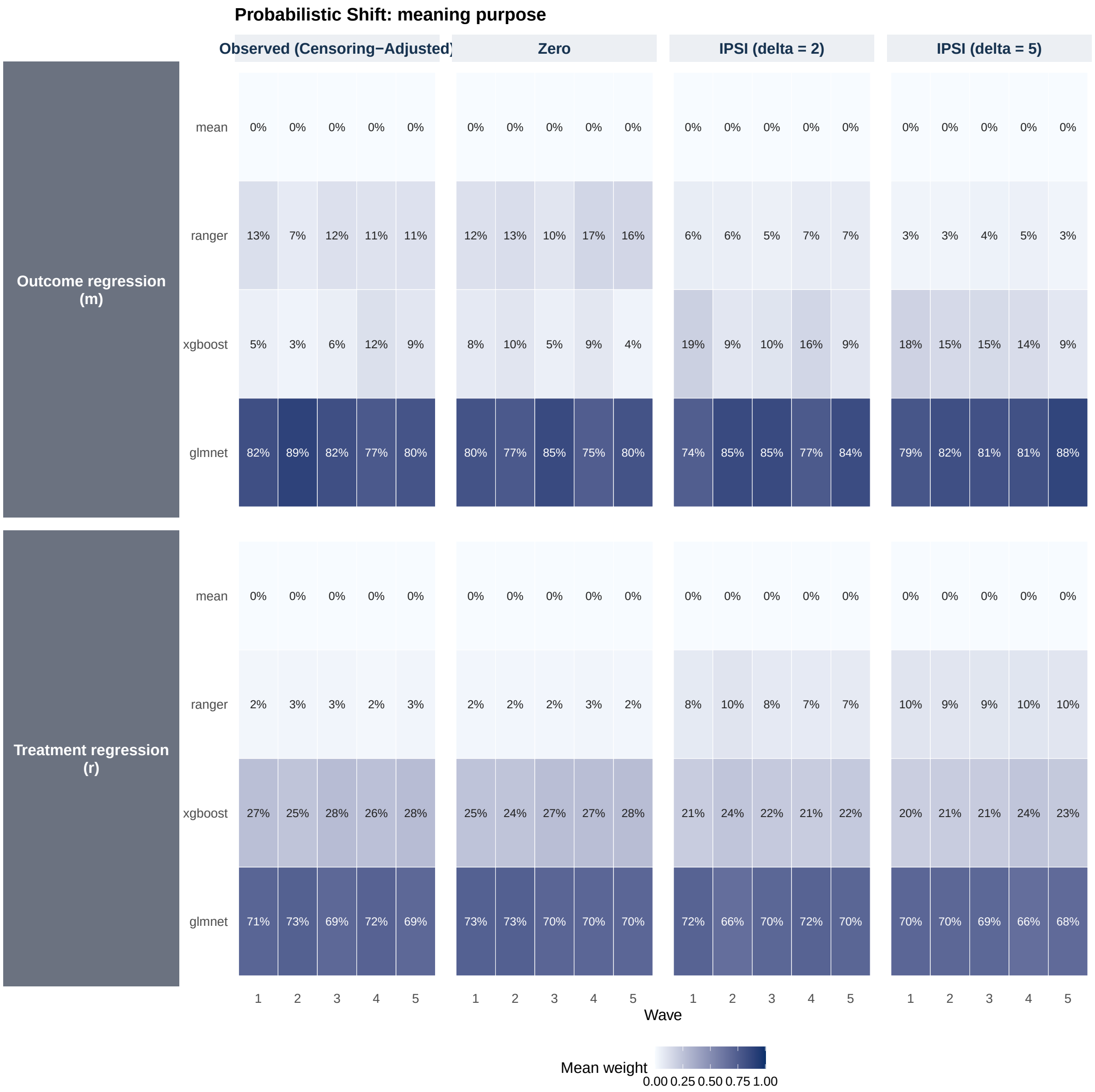

Figure 9: Learners: Incremental propensity score interventions on monthly religious attendance. Results for outcome regression in the top half of the figure. Results for treatment regression on the bottom.

Supplement S8: Deterministic Intervention Figures

This supplement presents figures and interpretations for the deterministic intervention results described in *Supplement S6 Part B*. As documented in *Supplement S5*, these interventions fail positivity diagnostics; estimates should be interpreted cautiously.

Target-trial 1: three-wave one-shot weekly attendance versus zero

The three-wave design imposes a single shift to weekly attendance at t_1 and evaluates outcomes at t_2, adjusting for baseline confounders at t_0. Confidence intervals and E-values were adjusted for multiple comparisons using Bonferroni correction ($\alpha = 0.05$). Weekly attendance yields reliable improvements across several outcomes with E-value lower bounds exceeding 1.10. Meaning increases for both purpose (0.119 [0.091, 0.147], 0.172 [0.131, 0.212], E-value 1.39) and sense (0.099 [0.070, 0.128], 0.118 [0.083, 0.153], E-value 1.33). Gratitude also rises (0.080 [0.051, 0.109], 0.069 [0.044, 0.095], E-value 1.27), as does forgiveness (0.076 [0.048, 0.104], 0.095 [0.060, 0.130], E-value 1.26). Alcohol use declines in both frequency (-0.073 [-0.095, -0.051], -0.099 [-0.129, -0.069], E-value 1.27) and intensity (-0.048 [-0.079, -0.017], -0.095 [-0.157, -0.034], E-value 1.14). Modest gains are observed for social support (0.041 [0.013, 0.069], 0.046 [0.015, 0.077], E-value 1.12), body satisfaction (0.039 [0.012, 0.066], 0.065 [0.020, 0.110], E-value 1.12), and belonging (0.038 [0.012, 0.064], 0.041 [0.013, 0.070], E-value 1.12). A causal design with temporal lag and rich adjustment yields effects substantially smaller than cross-sectional correlations, and the smaller effects concentrate in social-functional domains. However, as documented in Supplement S5, positivity diagnostics indicate that this causal estimand is not well supported by the data.

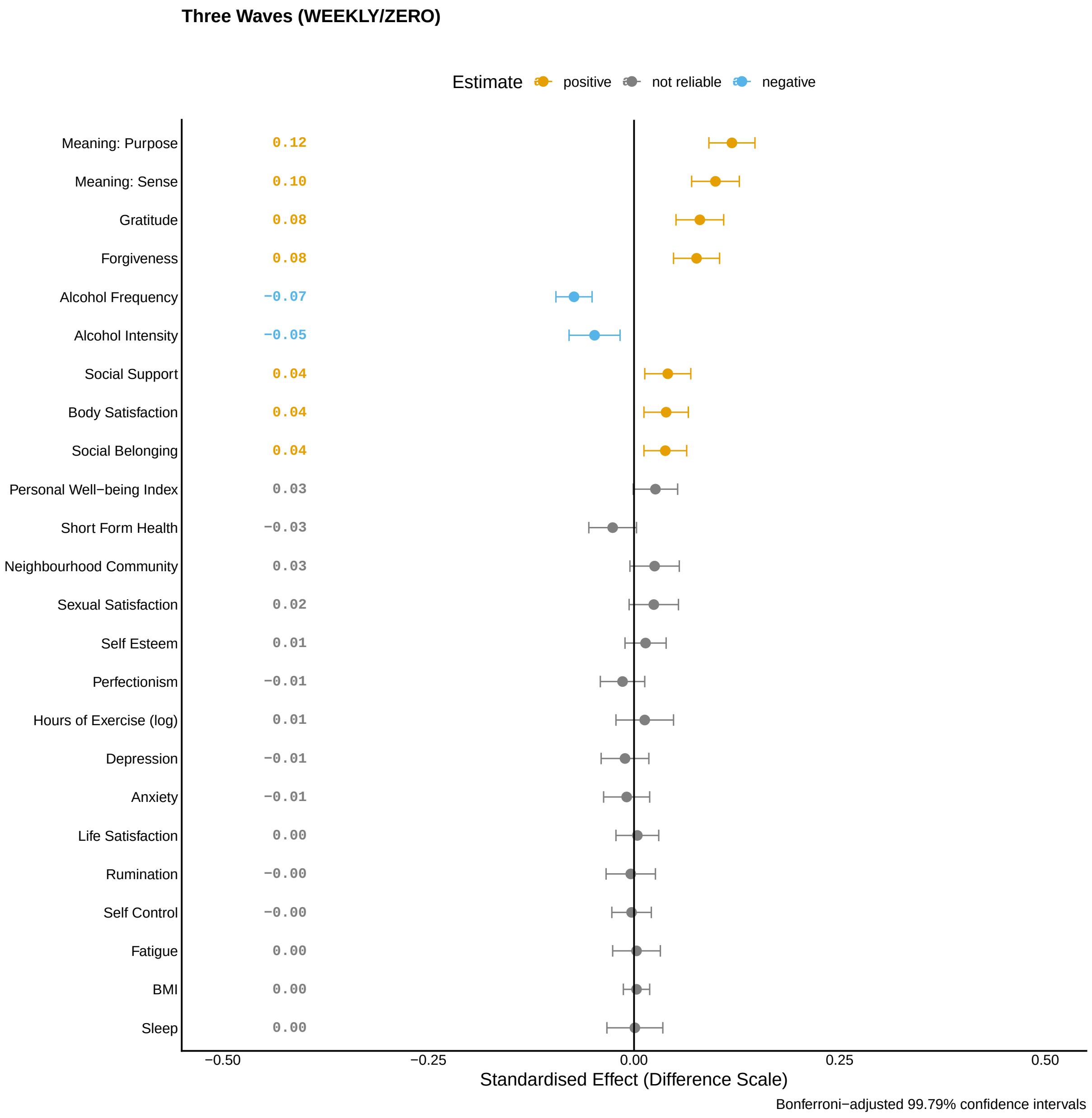

Figure 10: Three-wave deterministic shift (longitudinal modified treatment policy): weekly vs zero attendance in the full baseline cohort; outcomes are standardised (z).

Negative-control validation: three-wave increase in hours socialising

We next asked whether comparable gains could be produced by a secular increase in social time. The three-wave study that increases socialising by 1 hour, compared with the observed rate, hints at greater neighbourhood belonging but yields no reliable effects at the 1.10 E-value threshold. These results suggest that the attendance pattern does not arise solely from time spent with others. However, as documented in Supplement S5, the deterministic socialising intervention strains positivity; these results should be interpreted cautiously. (Losses of one hour per week socialising are similarly inconclusive: results reported in Supplement S6 Part B.)

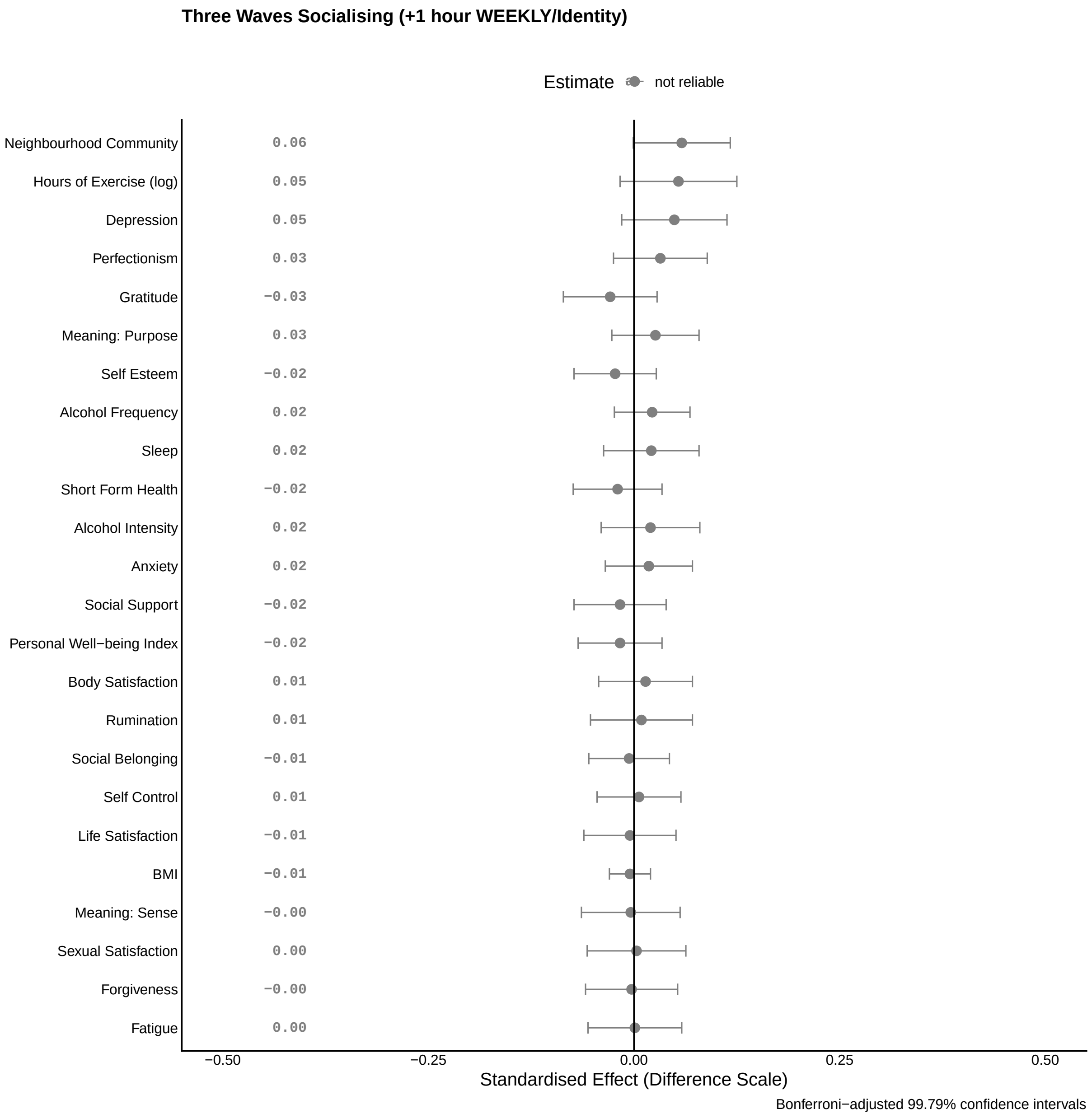

Figure 11: Three-wave negative control: +1 hour/week socialising (identity policy otherwise) in the full baseline cohort; reliable causal effects are not observed.

Target-trial 2: six-wave sustained weekly attendance versus zero

Sustained shifts to weekly attendance over the four exposure waves, implemented at the first and fourth waves, produce a profile of effects that is broadly consistent with the three-wave design but with modest amplification in a smaller set of outcomes (panel B). Reliable improvements are observed for meaning and purpose (0.106 [0.043, 0.169], 0.150 [0.061, 0.239], E-value 1.25) and sense (0.069 [0.011, 0.127], 0.083 [0.013, 0.152], E-value 1.11)-as well as for sexual satisfaction (0.100 [0.047, 0.153], 0.176 [0.083, 0.269], E-value 1.26) and forgiveness (0.065 [0.014, 0.116], 0.082 [0.018, 0.146], E-value 1.13). Alcohol frequency again decreases slightly (-0.037 [-0.072, -0.002], -0.062 [-0.108, -0.016], E-value 1.04). Other outcomes, including gratitude, the personal well-being index, neighbourhood community, and body satisfaction, show favourable effects but do not meet the E-value threshold for causal robustness after Bonferroni correction. Comparing the three-wave intervention (panel A) with this sustained four-exposure design shows similar qualitative signs, with a narrower set of reliable effects under sustained exposure. Social belonging and social acceptance do not reach reliability in this specification.

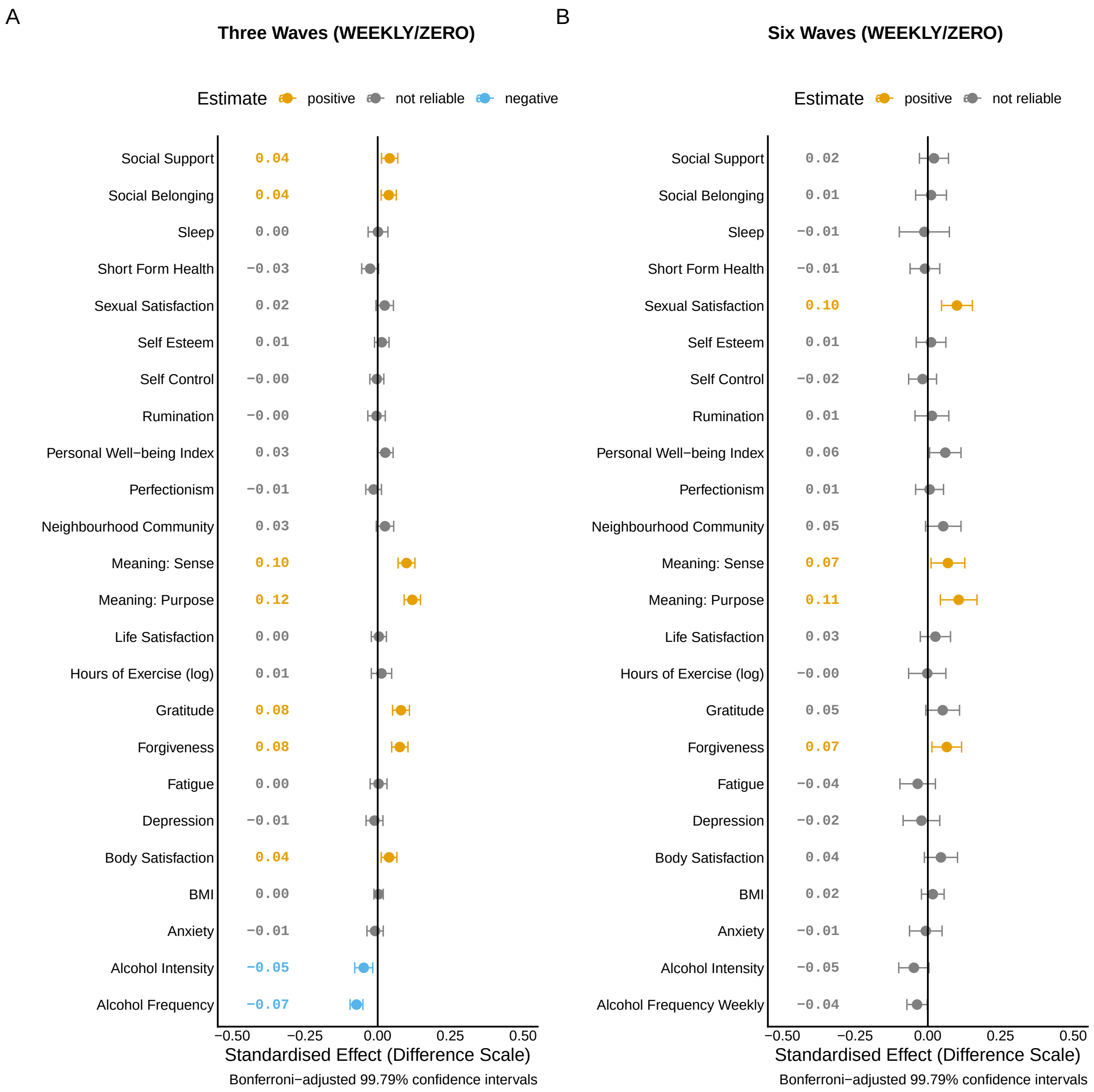

Figure 12: Six-wave deterministic shifts (longitudinal modified treatment policies): weekly vs zero attendance, each contrasted with the identity (no-change) policy in the full baseline cohort.

Target-trial 3 and Target-trial 4: six-wave 'all gain' sustained weekly attendance and 'all lose' attendance, each contrasted with the identity policy

Contrasting sustained weekly attendance with the identity policy clarifies how population-level changes in exposure compare against the status quo. Shifting everyone to weekly attendance, relative to no intervention (panel A), produces a pattern similar to that observed in the weekly-versus-zero comparisons but with smaller magnitudes. Reliable gains emerge for meaning-purpose (0.083 [0.037, 0.129], 0.117 [0.052, 0.183], E-value 1.22) and sense (0.062 [0.016, 0.108], 0.074 [0.019, 0.129], E-value 1.14)-as well as for sexual satisfaction (0.064 [0.021, 0.107], 0.112 [0.037, 0.188], E-value 1.16). Forgiveness also improves (0.049 [0.007, 0.091], 0.082 [0.018, 0.146], E-value 1.08), and a small positive effect is detectable for the personal well-being index (0.045 [0.005, 0.085], 0.072 [0.008, 0.136], E-value 1.07). Other outcomes-including gratitude, neighbourhood community, body satisfaction, and alcohol use-show effects in expected directions but do not retain robustness once multiplicity-adjusted E-values are applied. This target trial, therefore, shows that if everyone were shifted to weekly attendance, modest improvements would emerge in a concentrated set of well-being domains.

By contrast, shifting everyone to zero attendance under the identity policy (panel B) yields no reliable evidence of differences across outcomes. The only estimate meeting the robustness threshold is a small decline in sexual satisfaction (-0.036 [-0.060, -0.012], E-value 1.12). All other confidence intervals span zero and show attenuated E-value bounds. In a population where non-attendance is already common, this result implies that a universal loss of attendance would not detectably change population-level well-being.

Taken together, these two target trials illustrate that different causal questions can lead to different answers. Gains from increased attendance and losses from reduced attendance do not simply mirror one another, because the effects are evaluated relative to the empirical distribution of exposure in the population. Analyses that focus solely on coefficients obscure this asymmetry. Target-trial contrasts make clear that the practical consequences of 'everyone attending weekly' differ from those of 'everyone ceasing attendance', and that these distinctions matter for interpretation and policy relevance.

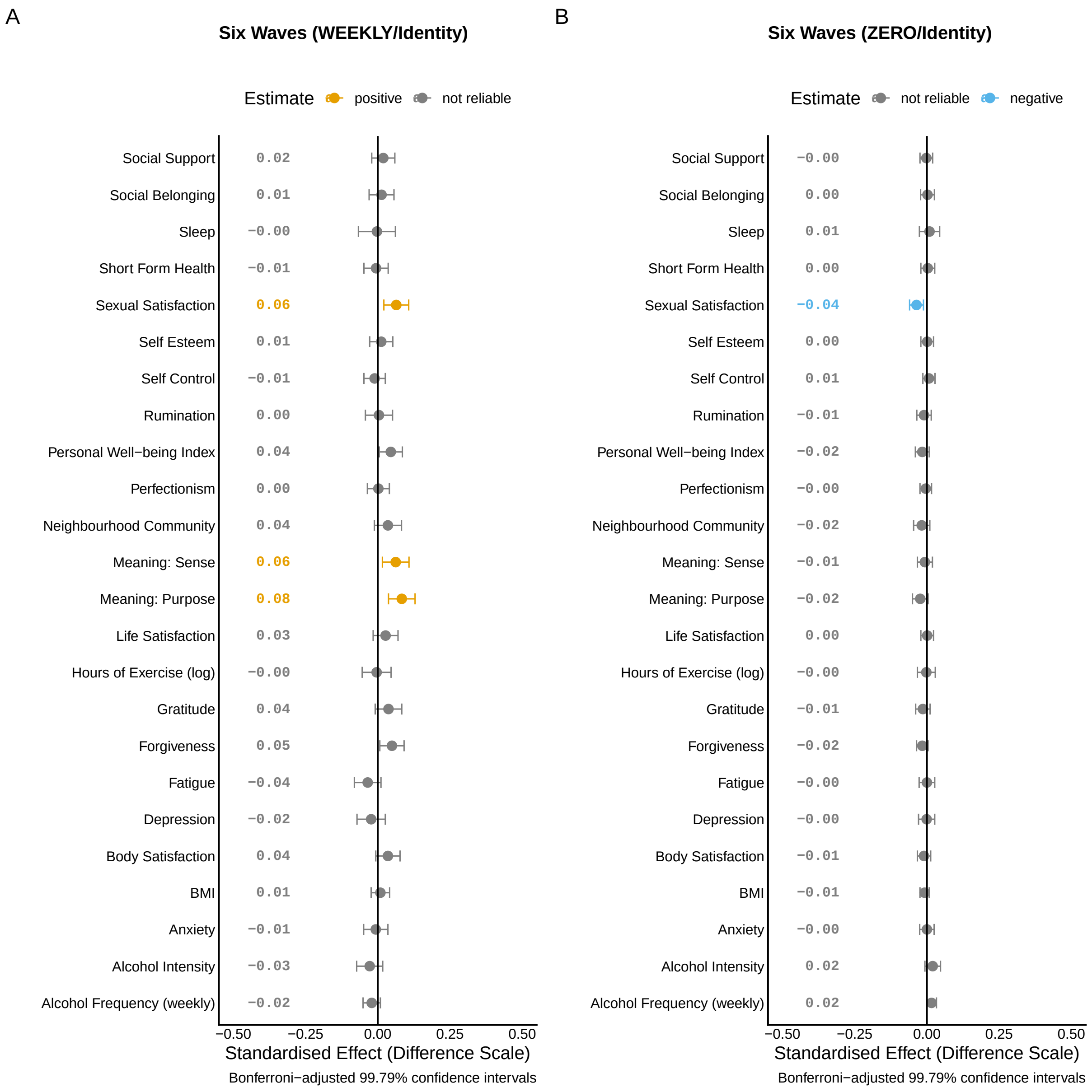

Figure 13: Six-wave deterministic shifts: weekly vs zero attendance, each contrasted with identity (no-change), full baseline cohort; loss-of-attendance effects differ from gains.

Target-trial 5 (behaviourally modest): six-wave monthly-or-more attendance versus zero

Sustained shifts to monthly attendance over the six exposure waves yield a narrower pattern of reliable effects than the weekly regime. Confidence intervals and E-values are adjusted for multiple comparisons using Bonferroni correction ($\alpha = 0.05$). The only outcome meeting the E-value threshold for causal robustness (lower bound > 1.10) is sexual satisfaction, which shows a modest but reliable gain (0.083 [0.024, 0.142], 0.146 [0.042, 0.249], E-value 1.17). Point estimates for other outcomes, including purpose in life (0.047 [-0.020, 0.114]), body satisfaction (0.040 [-0.019, 0.099]), gratitude (0.037 [-0.024, 0.098]), the personal well-being index (0.036 [-0.021, 0.093]), and neighbourhood community (0.034 [-0.032, 0.100]), tend to favour monthly attendance but remain small and imprecise, with confidence intervals spanning zero and E-value bounds at or below 1.00. Effects on alcohol use, mood, sleep, and other psychosocial outcomes are similarly inconclusive. These results suggest that sustained monthly attendance may yield modest benefits, particularly for sexual satisfaction, but does not reproduce the broader pattern of robust gains observed under sustained weekly attendance.

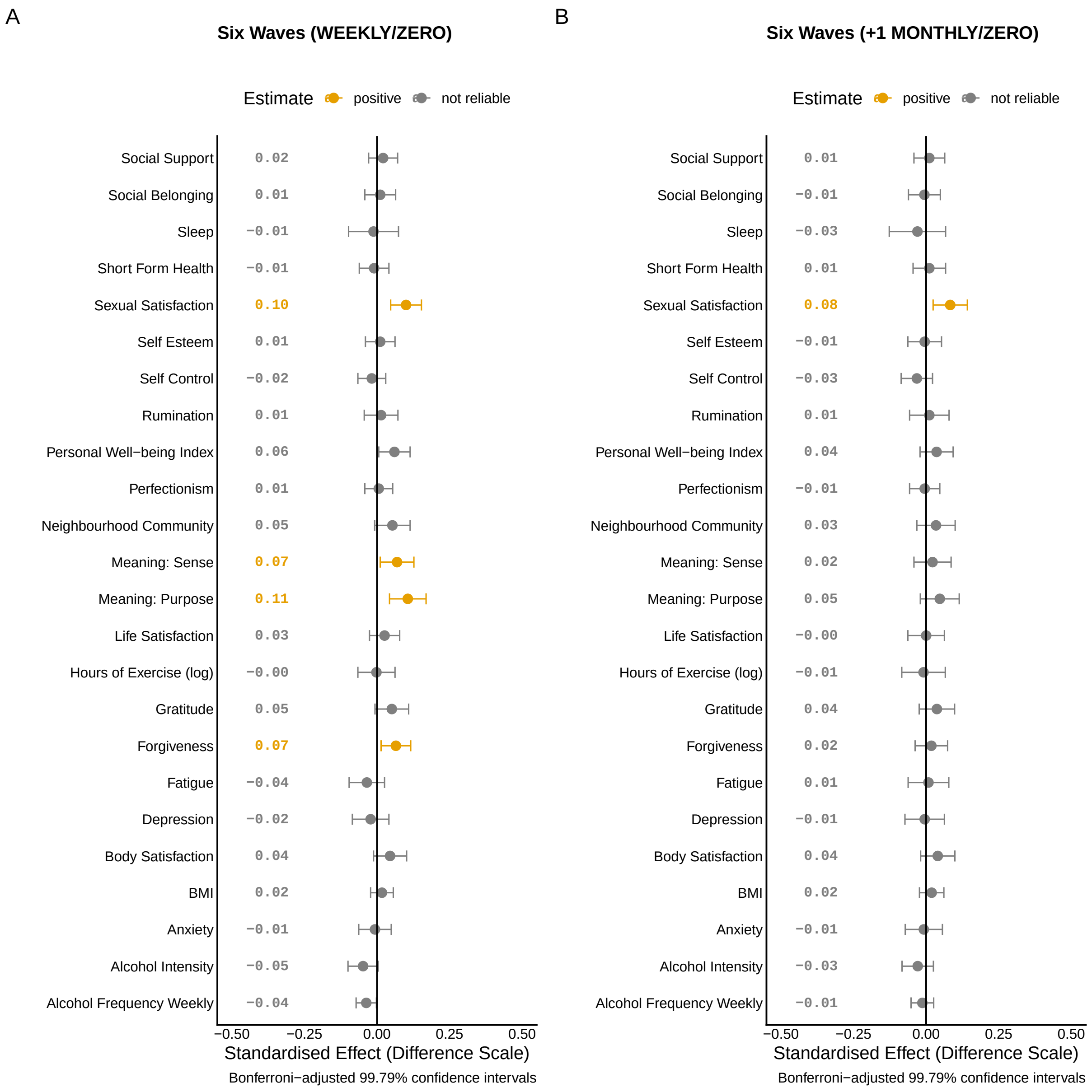

Figure 14: Six-wave deterministic shifts: weekly vs monthly attendance, each contrasted with zero; full baseline cohort.

Incremental propensity score intervention $\delta = 10$ (positivity-strained)

Although incremental propensity score interventions preserve theoretical positivity by construction, our empirical diagnostics indicate that the $\delta = 10$ regime strains practical support in this cohort, with 22.9% of uncensored trajectories having density-ratio products below 10^{-1}. We therefore present these results for transparency but do not treat them as central to our conclusions.

Figure 15 presents the results. For the $\delta = 10$ intervention, which applies a ten-fold increase in the conditional probability of initiating monthly attendance among non-attenders, reliable gains are observed for meaning-purpose (0.101 [0.059, 0.143]; 0.144 [0.084, 0.203]; E-value bound 1.29), sexual satisfaction (0.089 [0.047, 0.131]; 0.157 [0.083, 0.231]; E-value bound 1.26), forgiveness (0.081 [0.039, 0.123]; 0.102 [0.049, 0.156]; E-value bound 1.23), and meaning-sense (0.080 [0.032, 0.128]; 0.097 [0.039, 0.155]; E-value bound 1.21). short-form health (0.076 [0.034, 0.118]; 0.088 [0.039, 0.137]; E-value bound 1.21) and body satisfaction (0.072 [0.033, 0.111]; 0.122 [0.056, 0.187]; E-value bound 1.21) also meet the robustness criterion. A small reduction in body mass index (-0.034 [-0.065, -0.003]) and in weekly alcohol frequency (-0.032 [-0.062, -0.002]) emerges, but their E-value bounds (1.06 and 1.05) fall below 1.10, and other outcomes remain small or imprecise.

The pattern is consistent with, but amplified relative to, the $\delta = 5$ intervention, as expected given the more aggressive shift. However, given the concerns about positivity, these estimates should be interpreted cautiously.

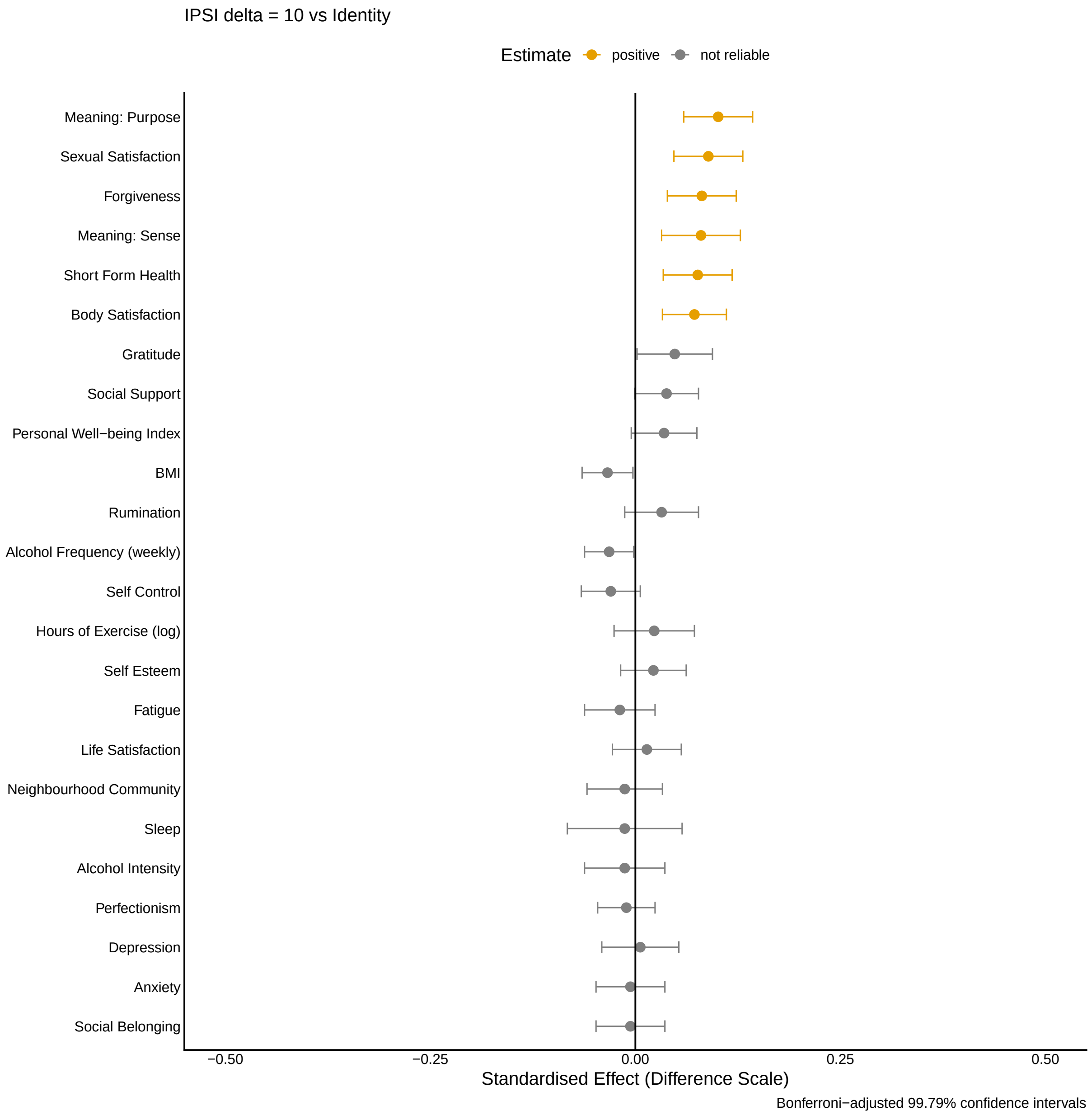

Figure 15: Incremental propensity score intervention $\delta = 10$ (positivity-strained): effects of a ten-fold increase in the conditional probability of initiating monthly attendance among baseline non-attenders, contrasted with the identity policy.

References

Altemeyer, B (1996) *The authoritarian spectre*, London: Harvard University Press.

Atkinson, J, Salmond, C, and Crampton, P (2019) *NZDep2018 index of deprivation, user's manual.*, Wellington.

Berry, JW, Worthington Jr., EL, O'Connor, LE, Parrott III, L, and Wade, NG (2005) Forgivingness, vengeful rumination, and affective traits. *Journal of Personality*, **73**(1), 183–226. doi:10.1111/j.1467-6494.2004.00308.x.

Bulbulia, JA (2024a) *Margot: MARGinal observational treatment-effects*. doi:10.5281/zenodo.10907724.

Bulbulia, JA (2024b) Methods in causal inference part 1: Causal diagrams and confounding. *Evolutionary Human Sciences*, **6**, e40. doi:10.1017/ehs.2024.35.

Bulbulia, JA (2024c) Methods in causal inference part 2: Interaction, mediation, and time-varying treatments. *Evolutionary Human Sciences*, **6**, e41. doi:10.1017/ehs.2024.32.

Bulbulia, JA (2024d) Methods in causal inference part 4: Confounding in experiments. *Evolutionary Human Sciences*, **6**, e43. doi:10.1017/ehs.2024.34.

Bulbulia, JA, Davis, DE, Park, CL, … Sibley, CG (2026) Target trial emulation shows that supported causal effects of religious attendance on well-being are selective. *Evolutionary Human Sciences*, **8**. doi:10.1017/ehs.2026.10043.

Burton, A, and Altman, DG (2004) Missing covariate data within cancer prognostic studies: A review of current reporting and proposed guidelines. *British Journal of Cancer*, **91**(1), 4–8. doi:10.1038/sj.bjc.6601907.

Buysse, DJ, Reynolds III, CF, Monk, TH, Berman, SR, and Kupfer, DJ (1989) The pittsburgh sleep quality index: A new instrument for psychiatric practice and research. *Psychiatry Research*, **28**(2), 193–213.

Caprara, GV (1986) Indicators of aggression: The dissipation-rumination scale. *Personality and Individual Differences*, **7**(6), 763–769. doi:10.1016/0191-8869(86)90074-7.

Chen, T, He, T, Benesty, M, … Yuan, J (2023) *Xgboost: Extreme gradient boosting*. Retrieved from https://CRAN.R-project.org/package=xgboost

Chernozhukov, V, Chetverikov, D, Demirer, M, … Robins, J (2018) Double/debiased machine learning for treatment and structural parameters., Oxford University Press Oxford, UK.

Díaz, I, Williams, N, Hoffman, KL, and Schenck, EJ (2021) Non-parametric causal effects based on longitudinal modified treatment policies. *Journal of the American Statistical Association*. doi:10.1080/01621459.2021.1955691.

Díaz, I, Williams, N, Hoffman, KL, and Schenck, EJ (2023) Nonparametric causal effects based on longitudinal modified treatment policies. *Journal of the American Statistical Association*, **118**(542), 846–857. doi:10.1080/01621459.2021.1955691.

Diener, E, Emmons, RA, Larsen, RJ, and Griffin, S (1985) The satisfaction with life scale. *Journal of Personality Assessment*, **49**(1), 71–75.

Fahy, KM, Lee, A, and Milne, BJ (2017) *New Zealand socio-economic index 2013*, Wellington, New Zealand: Statistics New Zealand-Tatauranga Aotearoa.

Fraser, G, Bulbulia, J, Greaves, LM, Wilson, MS, and Sibley, CG (2020) Coding responses to an open-ended gender measure in a New Zealand national sample. *The Journal of Sex Research*, **57**(8), 979–986. doi:10.1080/00224499.2019.1687640.

Greaves, LM, Barlow, FK, Lee, CH, et al.others (2017) The diversity and prevalence of sexual orientation self-labels in a New Zealand national sample. *Archives of Sexual Behavior*, **46**, 1325–1336.

Hagerty, BMK, and Patusky, K (1995) Developing a measure of sense of belonging: *Nursing Research*, **44**(1), 9–13. doi:10.1097/00006199-199501000-00003.

Health, Ministry of (2013) *The New Zealand Health Survey: Content guide 2012-2013*, Princeton University Press.

Hernan, MA, and Robins, JM (2020) *Causal inference*, Taylor & Francis. Retrieved from https://books.google.co.nz/books?id=/_KnHIAAACAAJ

Hoffman, KL, Salazar-Barreto, D, Williams, NT, Rudolph, KE, and Díaz, I (2024) Studying continuous, time-varying, and/or complex exposures using longitudinal modified treatment policies. *Epidemiology*, **35**(5), 667–675. doi:10.1097/EDE.0000000001764.

Hoffman, KL, Schenck, EJ, Satlin, MJ, … Díaz, I (2022) Comparison of a target trial emulation framework vs cox regression to estimate the association of corticosteroids with COVID-19 mortality. *JAMA Network Open*, **5**(10), e2234425. doi:10.1001/jamanetworkopen.2022.34425.

Kennedy, EH (2019) Nonparametric causal effects based on incremental propensity score interventions. *Journal of the American Statistical Association*, **114**(526), 645–656.

Kessler, RC, Andrews, G, Colpe, LJ, … Zaslavsky, AM (2002) Short screening scales to monitor population prevalences and trends in non-specific psychological distress. *Psychological Medicine*, **32**(6), 959–976.

doi:10.1017/S0033291702006074.
Linden, A, Mathur, MB, and VanderWeele, TJ (2020) Conducting sensitivity analysis for unmeasured confounding in observational studies using e-values: The evalue package. *The Stata Journal*, **20**(1), 162–175.
Lu, H, Cole, SR, Howe, CJ, and Westreich, D (2022) Toward a Clearer Definition of Selection Bias When Estimating Causal Effects. *Epidemiology (Cambridge, Mass.)*, **33**(5), 699–706. doi:10.1097/EDE.0000000000001516.
McCullough, ME, Emmons, RA, and Tsang, J-A (2002) The grateful disposition: A conceptual and empirical topography. *Journal of Personality and Social Psychology*, **82**(1), 112–127. doi:10.1037/0022-3514.82.1.112.
McElreath, R (2020) *Statistical rethinking: A Bayesian course with examples in R and Stan*, 2nd edn, Boca Raton, FL: CRC Press, 612. doi:10.1201/9780429029608.
Montgomery, JM, Nyhan, B, and Torres, M (2018) How conditioning on posttreatment variables can ruin your experiment and what to do about it. *American Journal of Political Science*, **62**(3), 760–775. doi:10.1111/ajps.12357.
Morgan, SL, and Winship, C (2014) *Counterfactuals and causal inference: Methods and principles for social research*, 2nd edn, Cambridge: Cambridge University Press. doi:10.1017/CBO9781107587991.
Nolen-hoeksema, S, and Morrow, J (1993) Effects of rumination and distraction on naturally occurring depressed mood. *Cognition and Emotion*, **7**(6), 561–570. doi:10.1080/02699939308409206.
Polley, E, LeDell, E, Kennedy, C, and Laan, M van der L (2023a) *SuperLearner: Super learner prediction*. Retrieved from https://CRAN.R-project.org/package=SuperLearner
Polley, E, LeDell, E, Kennedy, C, and van der Laan, M (2023b) *SuperLearner: Super learner prediction*. Retrieved from https://github.com/ecpolley/SuperLearner
Rice, KG, Richardson, CME, and Tueller, S (2014) The short form of the revised almost perfect scale. *Journal of Personality Assessment*, **96**(3), 368–379. doi:10.1080/00223891.2013.838172.
Richardson, TS, and Robins, JM (2013) Single world intervention graphs: A primer. In, Citeseer. Retrieved from https://core.ac.uk/display/102673558
Robins, J, and Hernan, M (2008) Estimation of the causal effects of time-varying exposures. *Chapman & Hall/CRC Handbooks of Modern Statistical Methods*, 553–599.
Robins, JM, Greenland, S, and Hu, F-C (1999) Estimation of the causal effect of a time-varying exposure on the marginal mean of a repeated binary outcome. *Journal of the American Statistical Association*, **94**(447), 687–700. doi:10.1080/01621459.1999.10474168.
Rosa, PA de la, Cowden, RG, Bulbulia, JA, Sibley, CG, and VanderWeele, TJ (2024) Effects of screen-based leisure time on 24 subsequent health and wellbeing outcomes: A longitudinal outcome-wide analysis. *International Journal of Behavioral Medicine*, 1–20. doi:https://doi.org/10.1007/s12529-024-10307-0.
Rosenberg, M (1965) Rosenberg self-esteem scale (RSE). *Acceptance and Commitment Therapy. Measures Package*, **61**(52), 18.
Sengupta, NK, Luyten, N, Greaves, LM, … Sibley, CG (2013) Sense of community in New Zealand neighbourhoods: A multi-level model predicting social capital. *New Zealand Journal of Psychology*, **42**(1), 36–45.
Sibley, CG (2021) *Sampling procedure and sample details for the New Zealand Attitudes and Values Study*.
Sibley, CG, Afzali, MU, Satherley, N, … others (2020) Prejudice toward muslims in New Zealand: Insights from the New Zealand Attitudes and Values Study. *New Zealand Journal of Psychology*, **49**(1).
Sibley, CG, Luyten, N, Purnomo, M, … Robertson, A (2011) The mini-IPIP6: Validation and extension of a short measure of the big-six factors of personality in New Zealand. *New Zealand Journal of Psychology*, **40**(3), 142–159.
Sidanius, J, and Pratto, F (1999) *Social dominance: An intergroup theory of social hierarchy and oppression*, Cambridge: Cambridge University Press.
Statistics New Zealand (2017) *Statistical standard for geographic areas 2018 (SSGA18)*, Wellington, New Zealand: Statistics New Zealand. Retrieved from https://www.stats.govt.nz/methods/statistical-standard-for-geographic-areas-2018/
Steger, MF, Frazier, P, Oishi, S, and Kaler, M (2006) The meaning in life questionnaire: Assessing the presence of and search for meaning in life. *Journal of Counseling Psychology*, **53**(1), 80–93. doi:10.1037/0022-0167.53.1.80.
Stronge, S, Greaves, LM, Milojev, P, West-Newman, T, Barlow, FK, and Sibley, CG (2015) Facebook is linked to body dissatisfaction: Comparing users and non-users. *Sex Roles*, **73**, 200–213.
Van Buuren, S (2018) *Flexible imputation of missing data*, CRC press.
Van Der Laan, MJ, and Rose, S (2011) *Targeted learning: Causal inference for observational and experimental data*, New York, NY: Springer. Retrieved from https://link.springer.com/10.1007/978-1-4419-9782-1
Van Der Laan, MJ, and Rose, S (2018) *Targeted learning in data science: Causal inference for complex longitudinal*

studies, Cham: Springer International Publishing. Retrieved from http://link.springer.com/10.1007/978-3-319-65304-4

VanderWeele, TJ (2009) Concerning the consistency assumption in causal inference. *Epidemiology*, **20**(6), 880. doi:10.1097/EDE.0b013e3181bd5638.

VanderWeele, TJ (2012) Confounding and effect modification: Distribution and measure. *Epidemiologic Methods*, **1**(1), 55–82. doi:10.1515/2161-962X.1004.

VanderWeele, TJ (2015) *Explanation in causal inference: Methods for mediation and interaction*, Oxford University Press.

VanderWeele, TJ (2019) Principles of confounder selection. *European Journal of Epidemiology*, **34**(3), 211–219.

VanderWeele, TJ, and Ding, P (2017) Sensitivity analysis in observational research: Introducing the E-value. *Annals of Internal Medicine*, **167**(4), 268–274. doi:10.7326/M16-2607.

VanderWeele, TJ, Mathur, MB, and Chen, Y (2020) Outcome-wide longitudinal designs for causal inference: A new template for empirical studies. *Statistical Science*, **35**(3), 437–466.

Verbrugge, LM (1997) A global disability indicator. *Journal of Aging Studies*, **11**(4), 337–362. doi:10.1016/S0890-4065(97)90026-8.

Whitehead, J, Davie, G, Graaf, B de, … Nixon, G (2023) Unmasking hidden disparities: A comparative observational study examining the impact of different rurality classifications for health research in Aotearoa New Zealand. *BMJ Open*, **13**(4), e067927.

Williams, N, and Díaz, I (2023) lmtp: An R package for estimating the causal effects of modified treatment policies. *Observational Studies*, **9**(2), 103–122.

Williams, NT, and Díaz, I (2021) *lmtp: Non-parametric causal effects of feasible interventions based on modified treatment policies*. doi:10.5281/zenodo.3874931.

Wright, MN, and Ziegler, A (2017) ranger: A fast implementation of random forests for high dimensional data in C++ and R. *Journal of Statistical Software*, **77**(1), 1–17. doi:10.18637/jss.v077.i01.

Zhang, J, Dashti, SG, Carlin, JB, Lee, KJ, and Moreno-Betancur, M (2023) Should multiple imputation be stratified by exposure group when estimating causal effects via outcome regression in observational studies? *BMC Medical Research Methodology*, **23**(1), 42.
